# Supplementary material for: Associations of inflammation-related proteome with demographic and clinical characteristics of people with HIV in South Africa
Source: Proteomics Clin Appl. Author manuscript; Available in PMC 2026 Aug 3. (PMC13432066; doi:10.1002/prca.202300015)
Supplement: SupplementaryFigure [file NIHMS2196073-supplement-SupplementaryFigure.pdf]

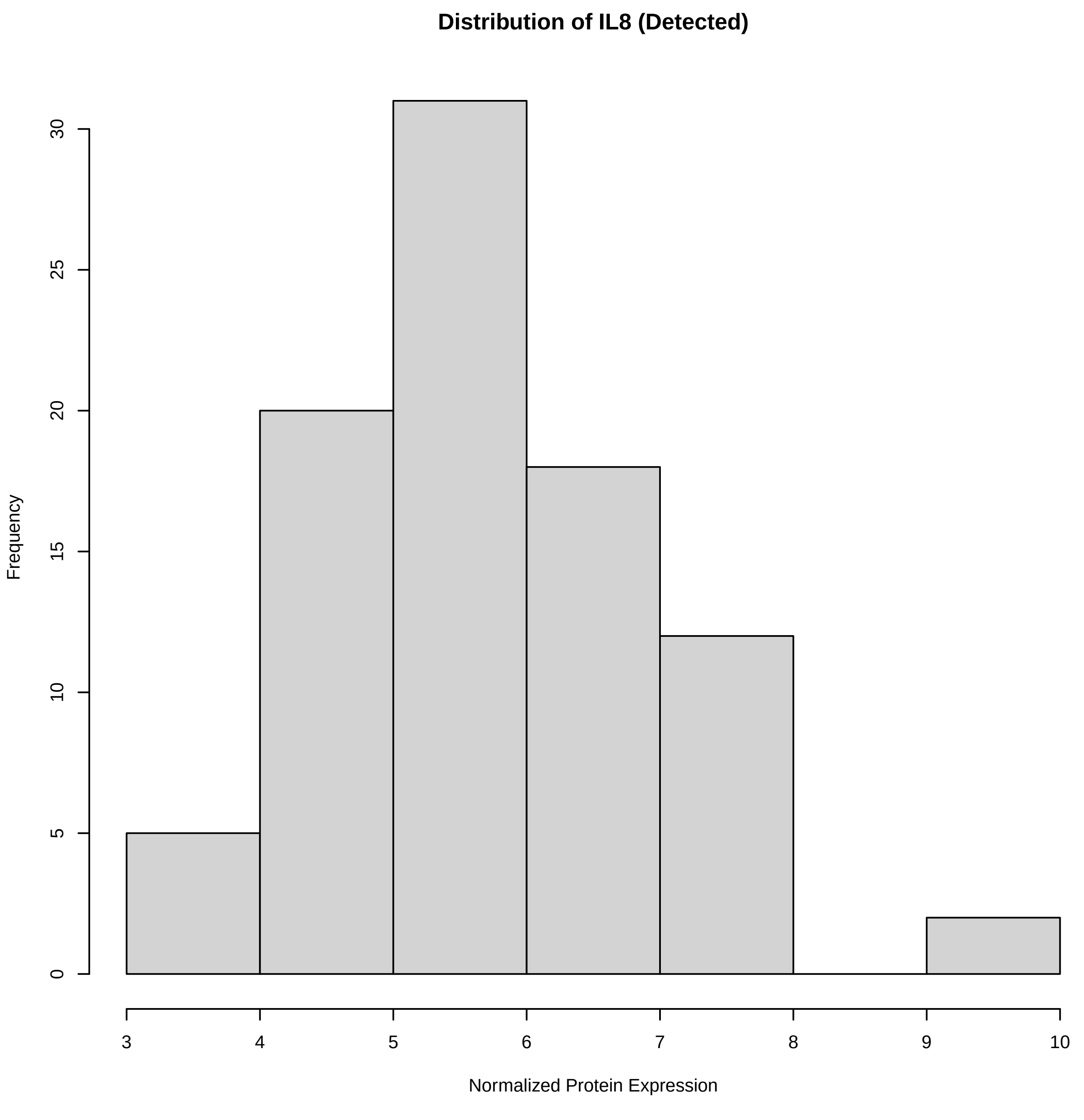

Distribution of IL8 (Detected)
Frequency
0
5
10
15
20
25
30
3
4
5
6
7
8
9
10
Normalized Protein Expression

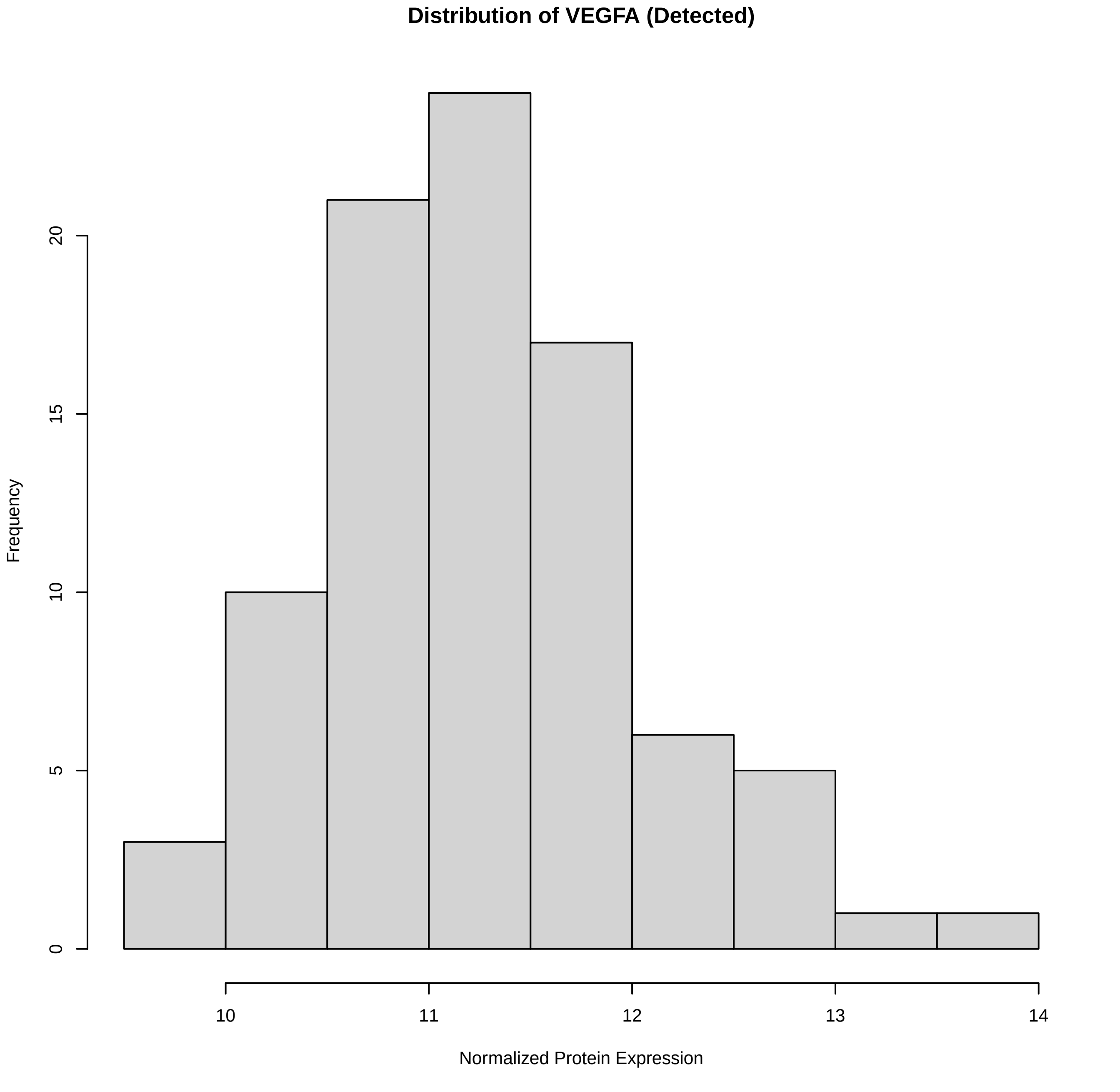
Distribution of VEGFA (Detected)
Frequency
0
5
10
15
20
10
11
12
13
14
Normalized Protein Expression

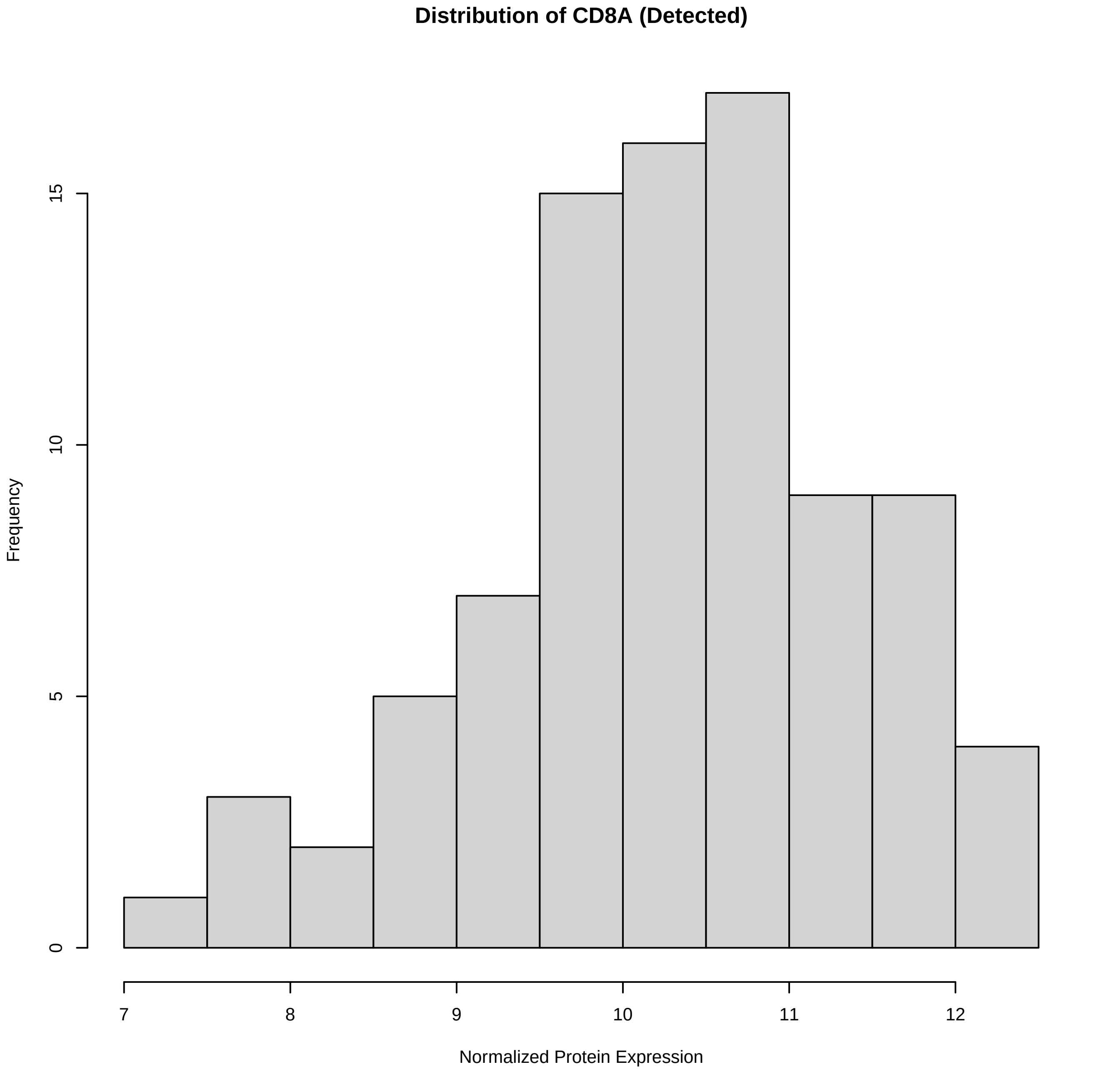
Distribution of CD8A (Detected)
Frequency
0
5
10
15
7
8
9
10
11
12
Normalized Protein Expression

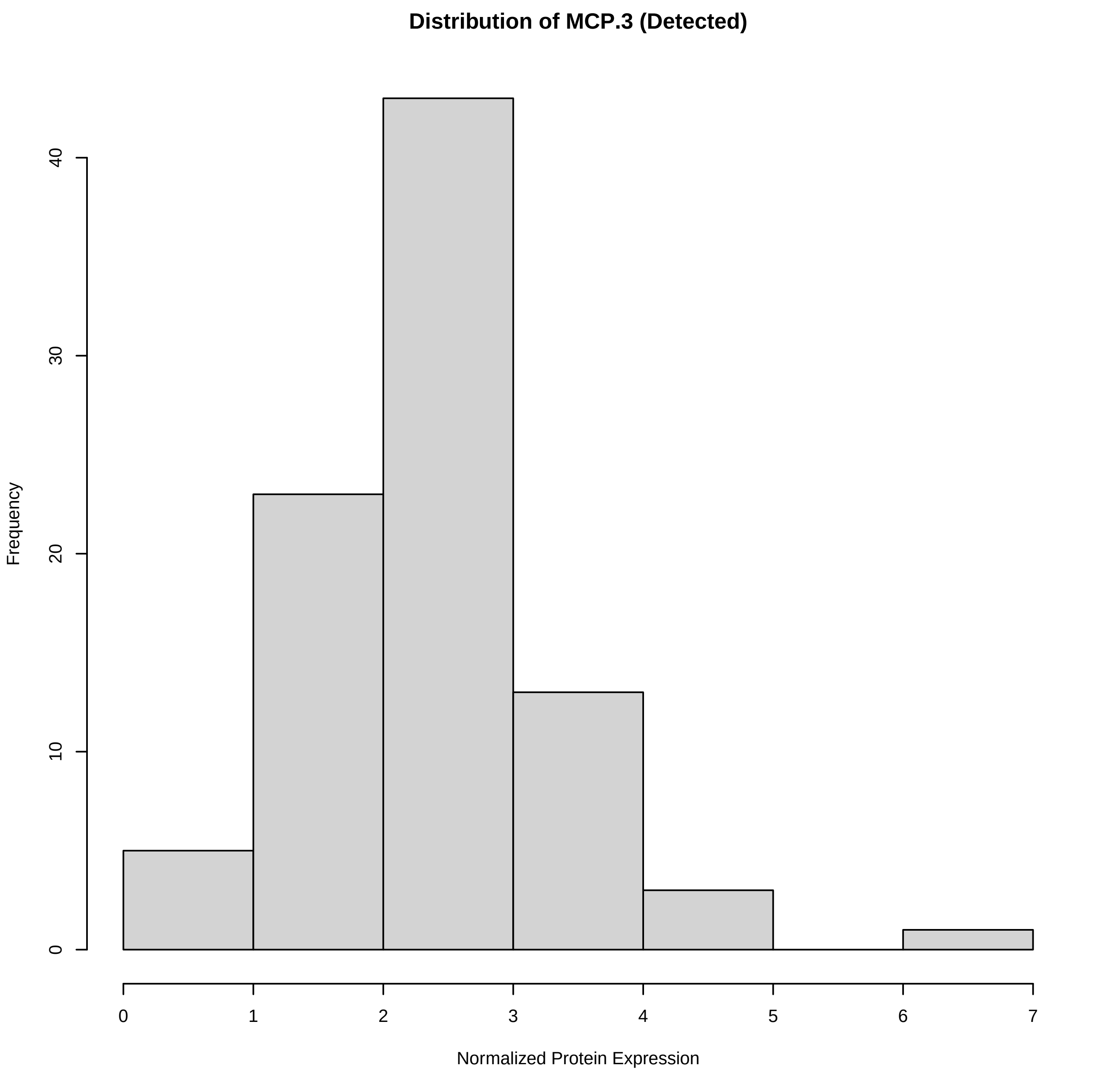

Distribution of MCP.3 (Detected)
Frequency
0
10
20
30
40
0
1
2
3
4
5
6
7
Normalized Protein Expression

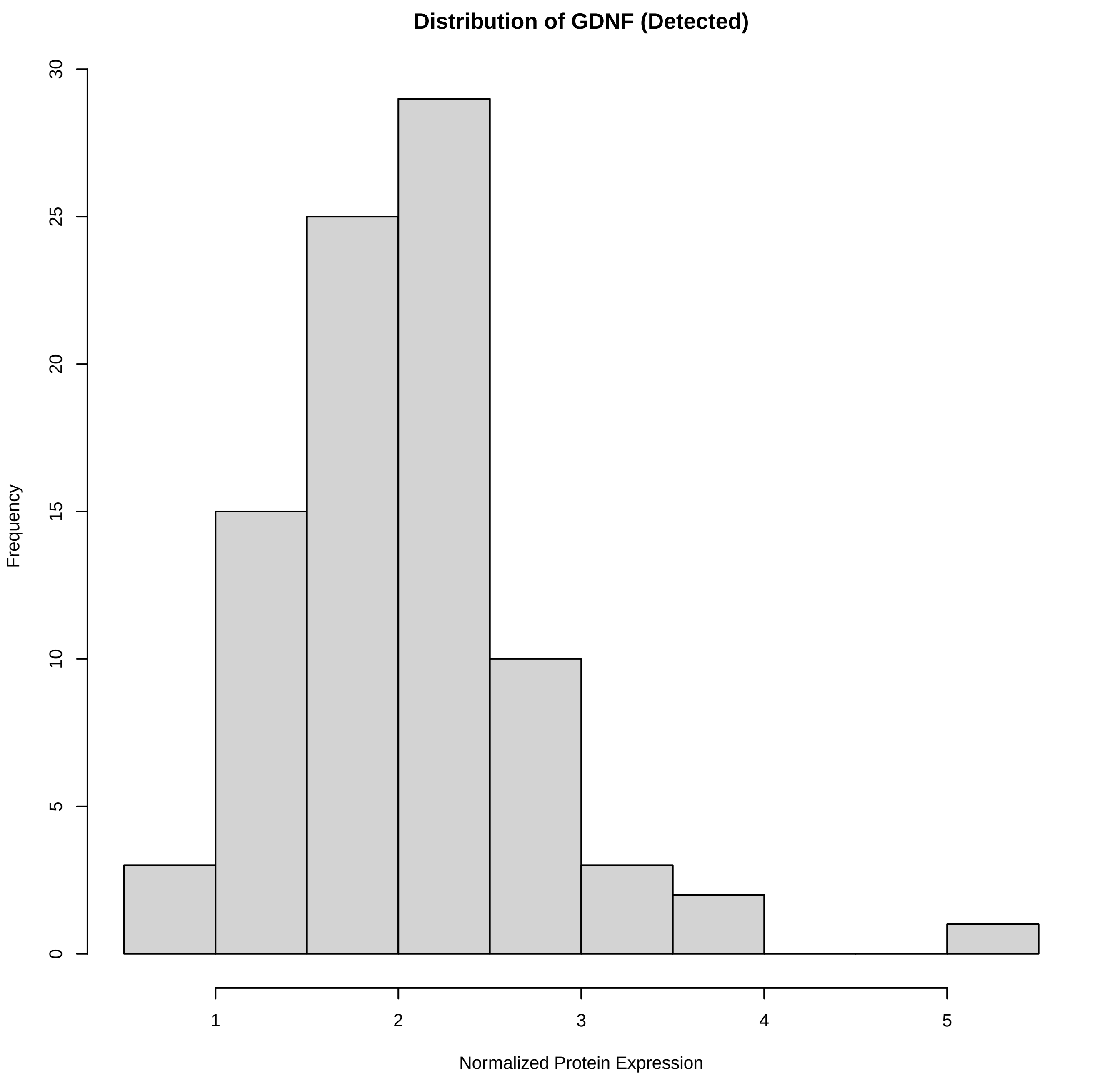
Distribution of GDNF (Detected)
Frequency
0
5
10
15
20
25
30
1
2
3
4
5
Normalized Protein Expression

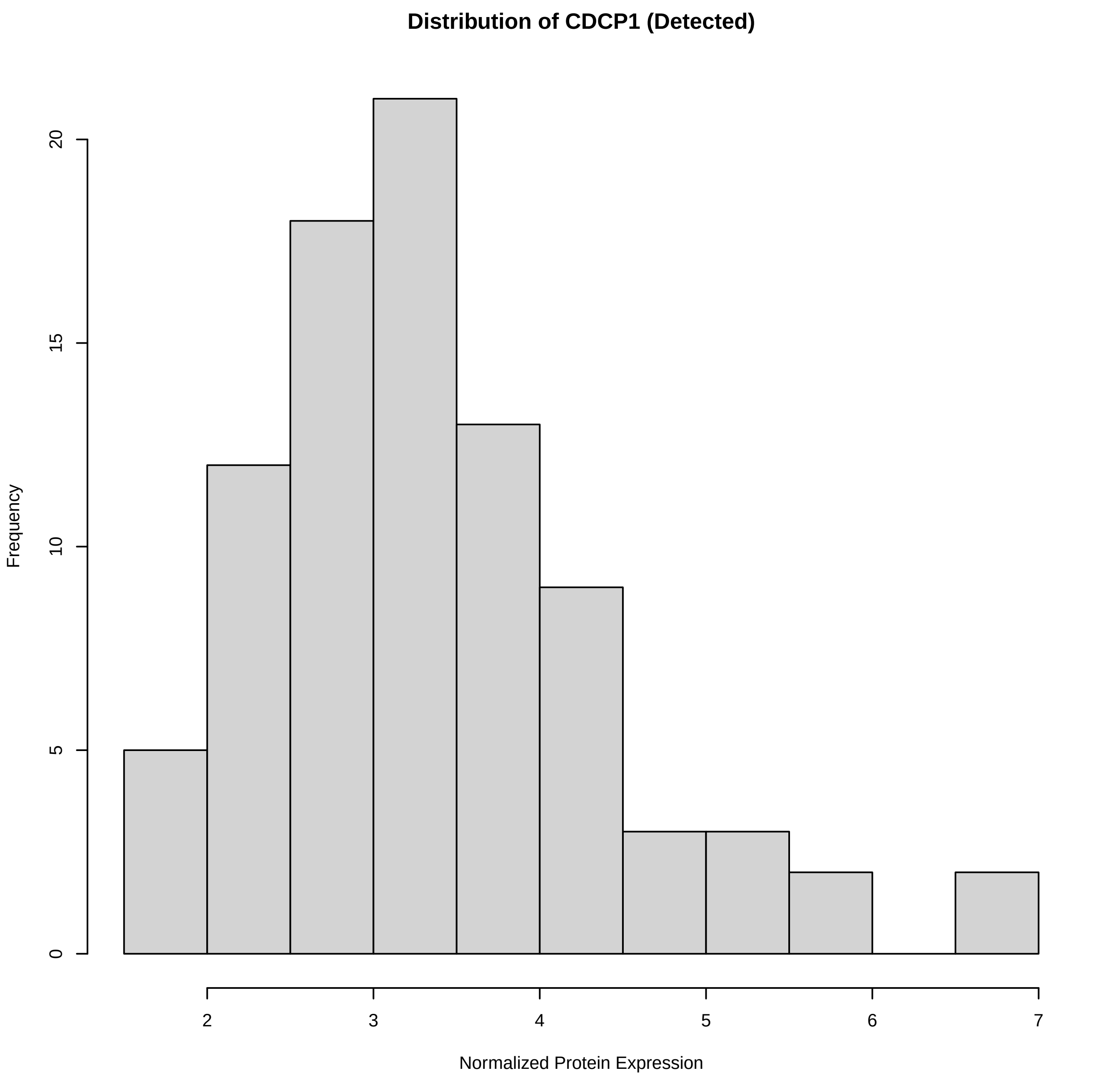
Distribution of CDCP1 (Detected)
Frequency
0
5
10
15
20
2
3
4
5
6
7
Normalized Protein Expression

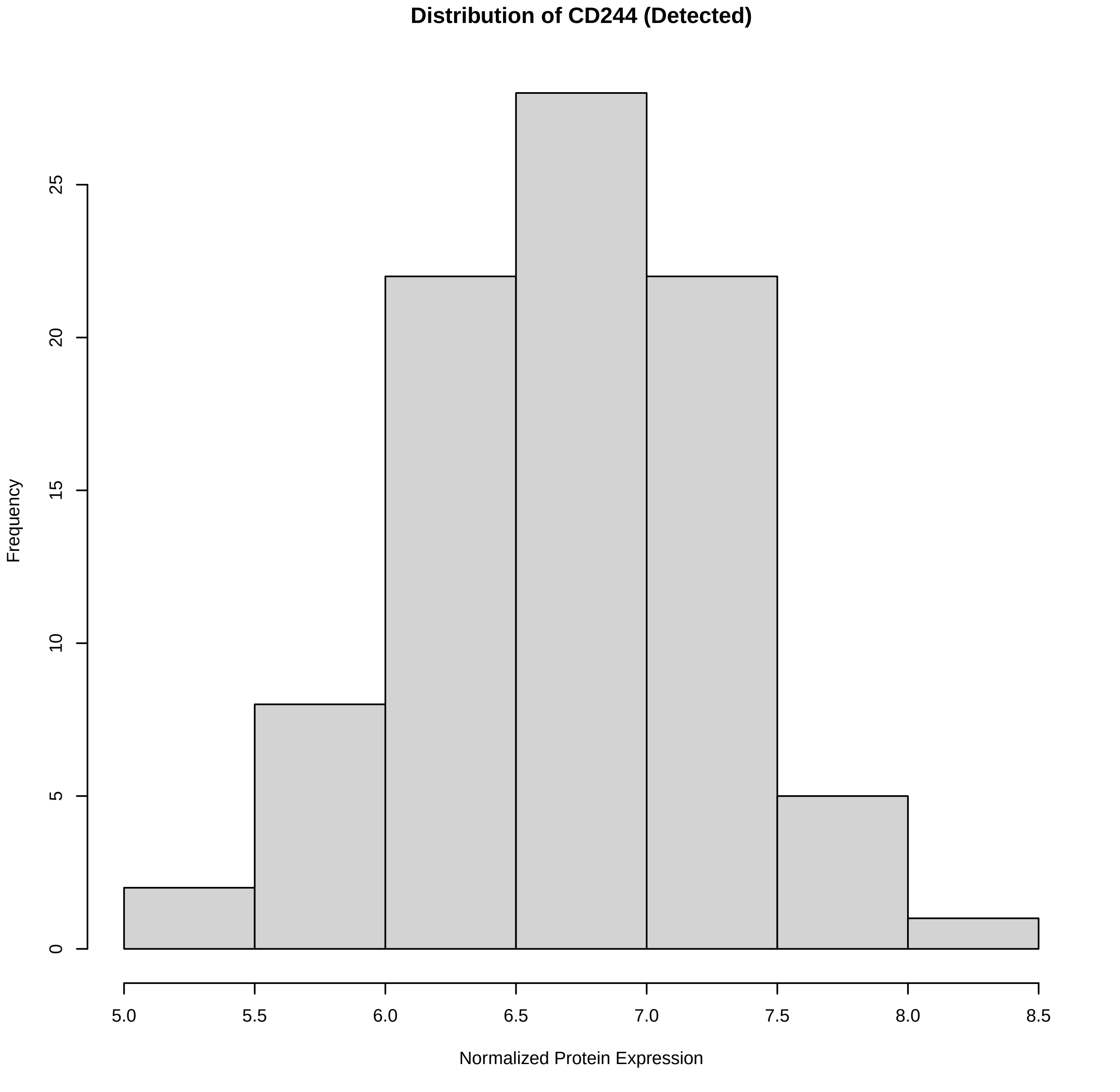
Distribution of CD244 (Detected)
Frequency
0
5
10
15
20
25
5.0
5.5
6.0
6.5
7.0
7.5
8.0
8.5
Normalized Protein Expression

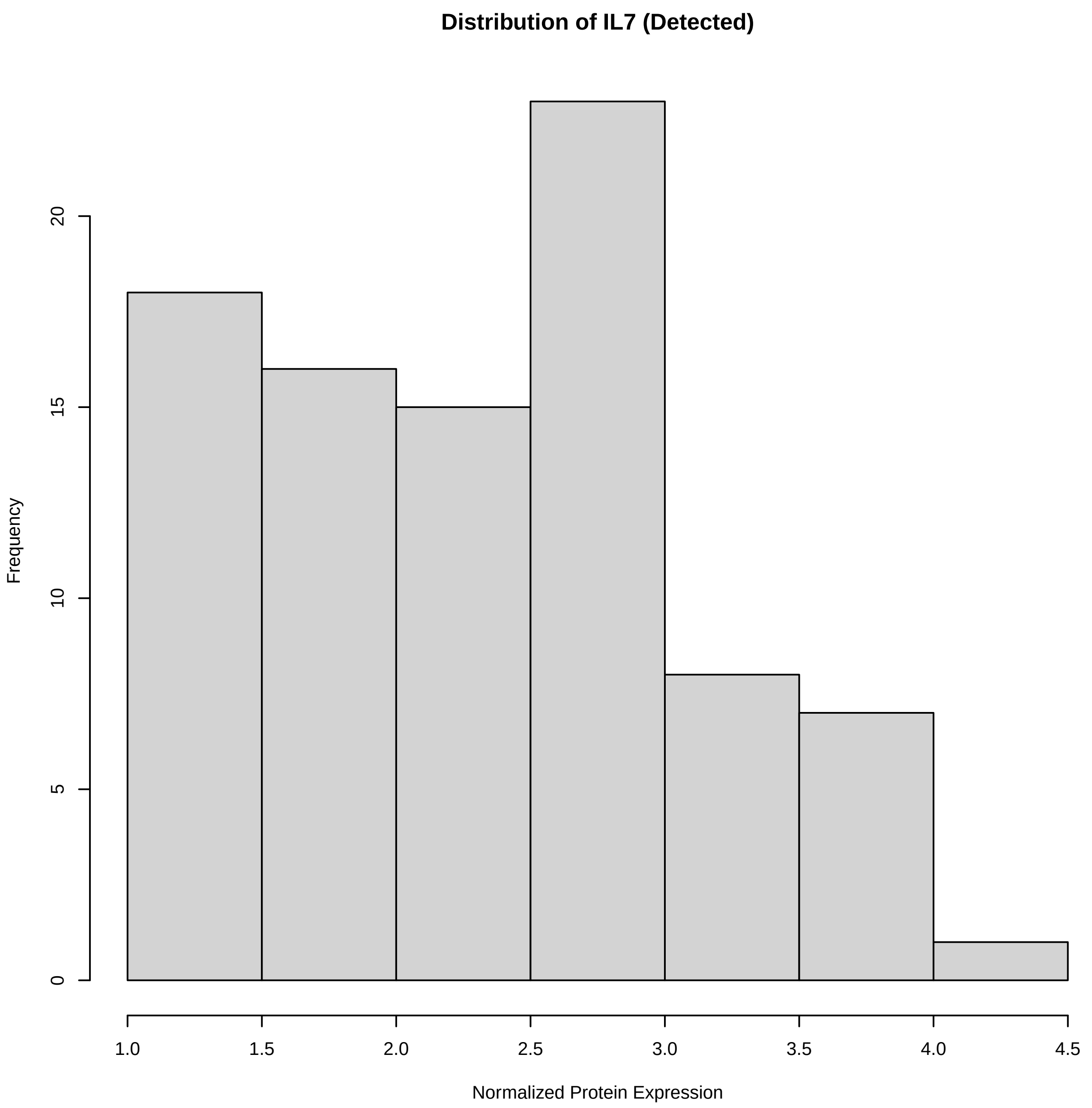
Distribution of IL7 (Detected)
Frequency
0
5
10
15
20
1.0
1.5
2.0
2.5
3.0
3.5
4.0
4.5
Normalized Protein Expression

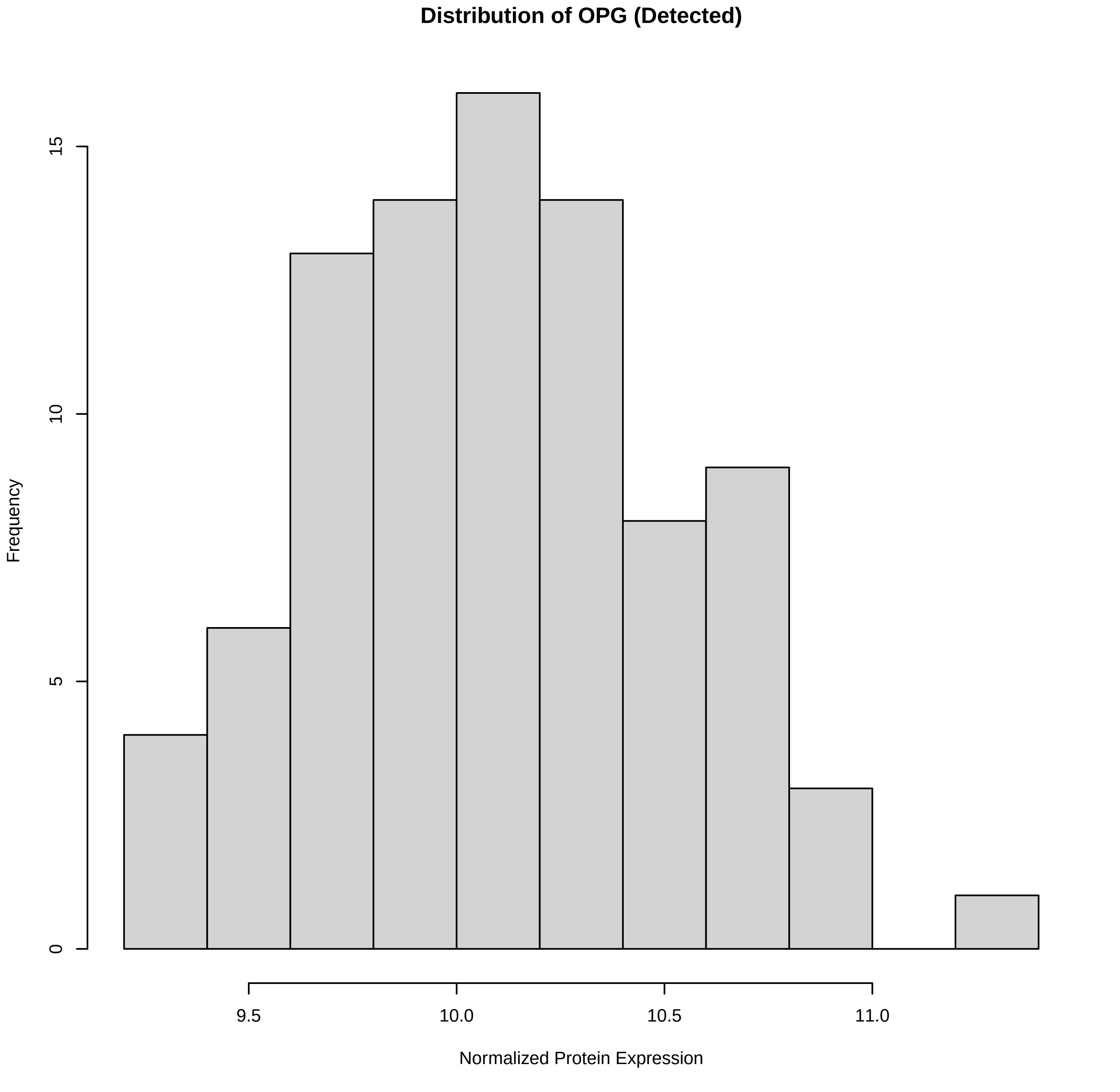

Distribution of OPG (Detected)
Frequency
0
5
10
15
9.5
10.0
10.5
11.0
Normalized Protein Expression

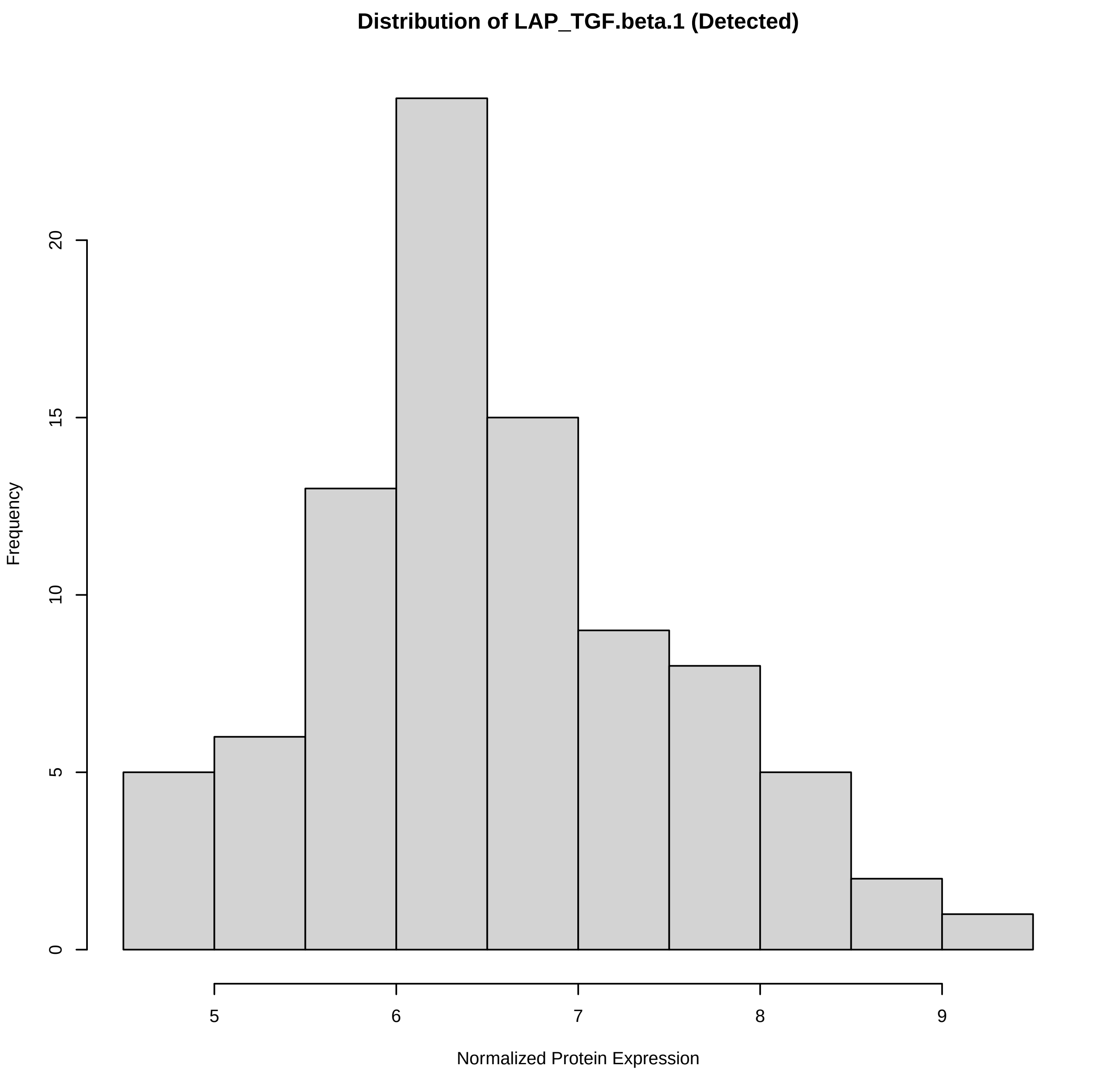
Distribution of LAP_TGF.beta.1 (Detected)
Frequency
0
5
10
15
20
5
6
7
8
9
Normalized Protein Expression

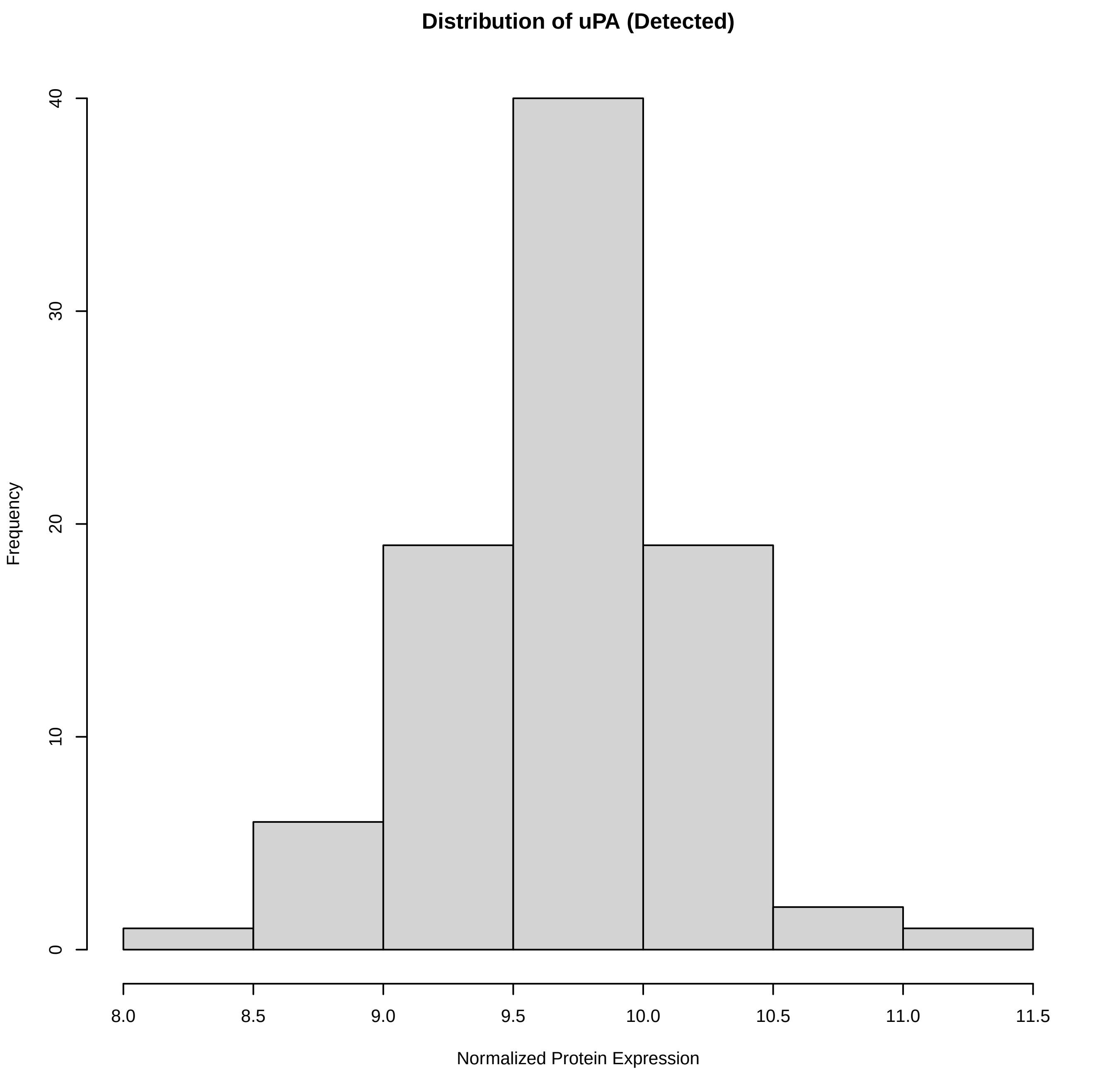
Distribution of uPA (Detected)
Frequency
0
10
20
30
40
8.0
8.5
9.0
9.5
10.0
10.5
11.0
11.5
Normalized Protein Expression

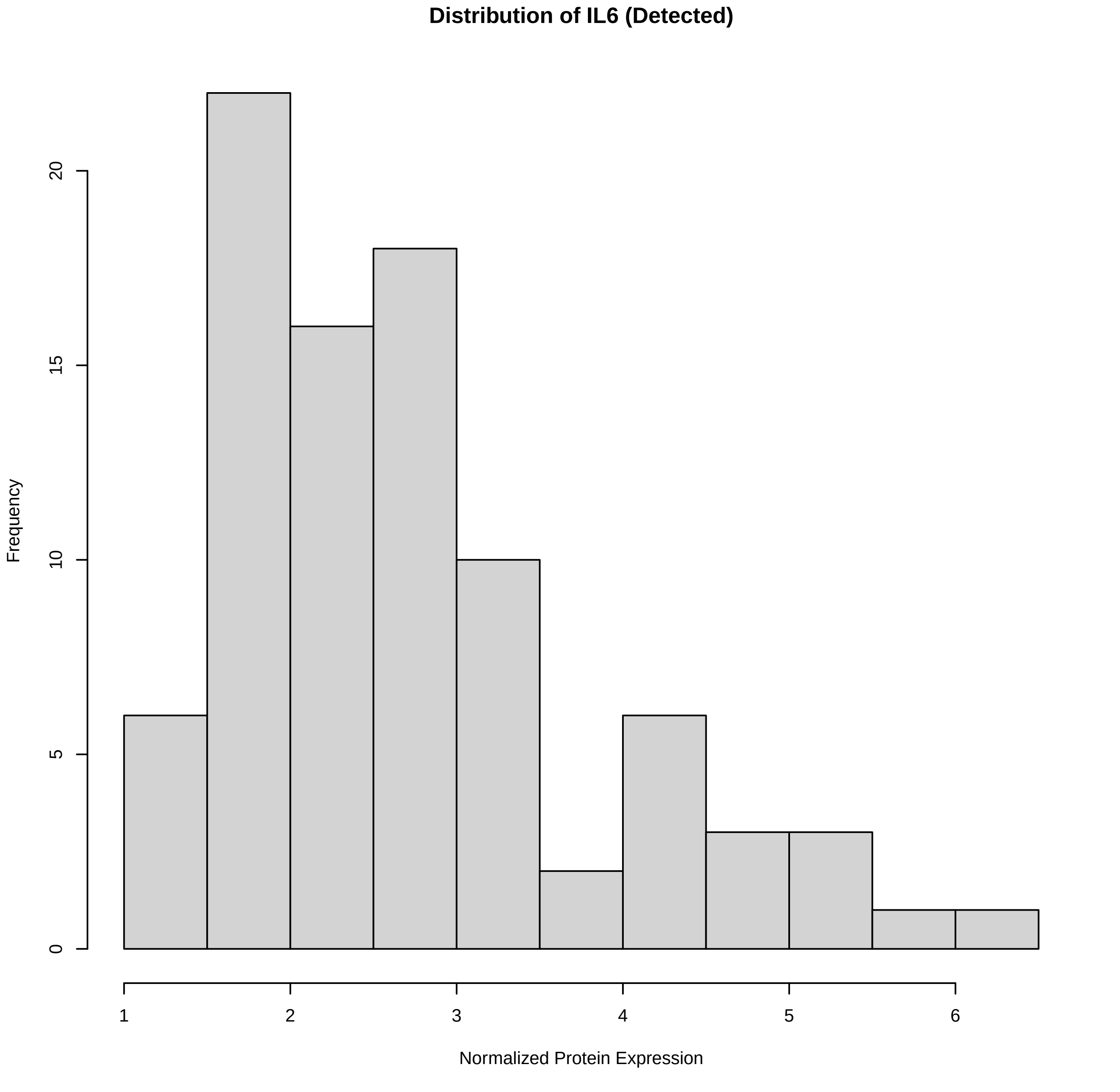
Distribution of IL6 (Detected)
Frequency
0
5
10
15
20
1
2
3
4
5
6
Normalized Protein Expression

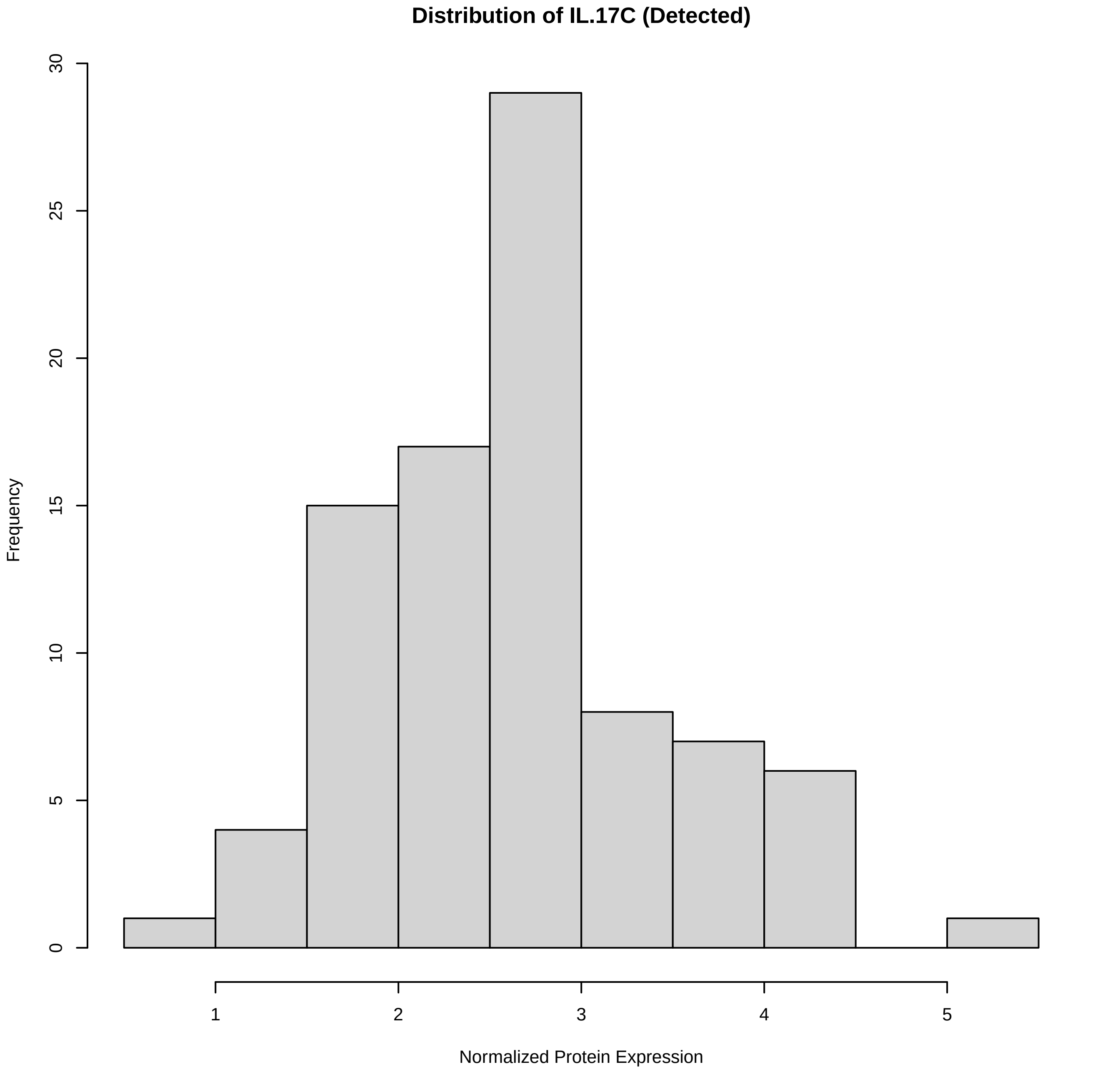

Distribution of IL.17C (Detected)
Frequency
0
5
10
15
20
25
30
1
2
3
4
5
Normalized Protein Expression

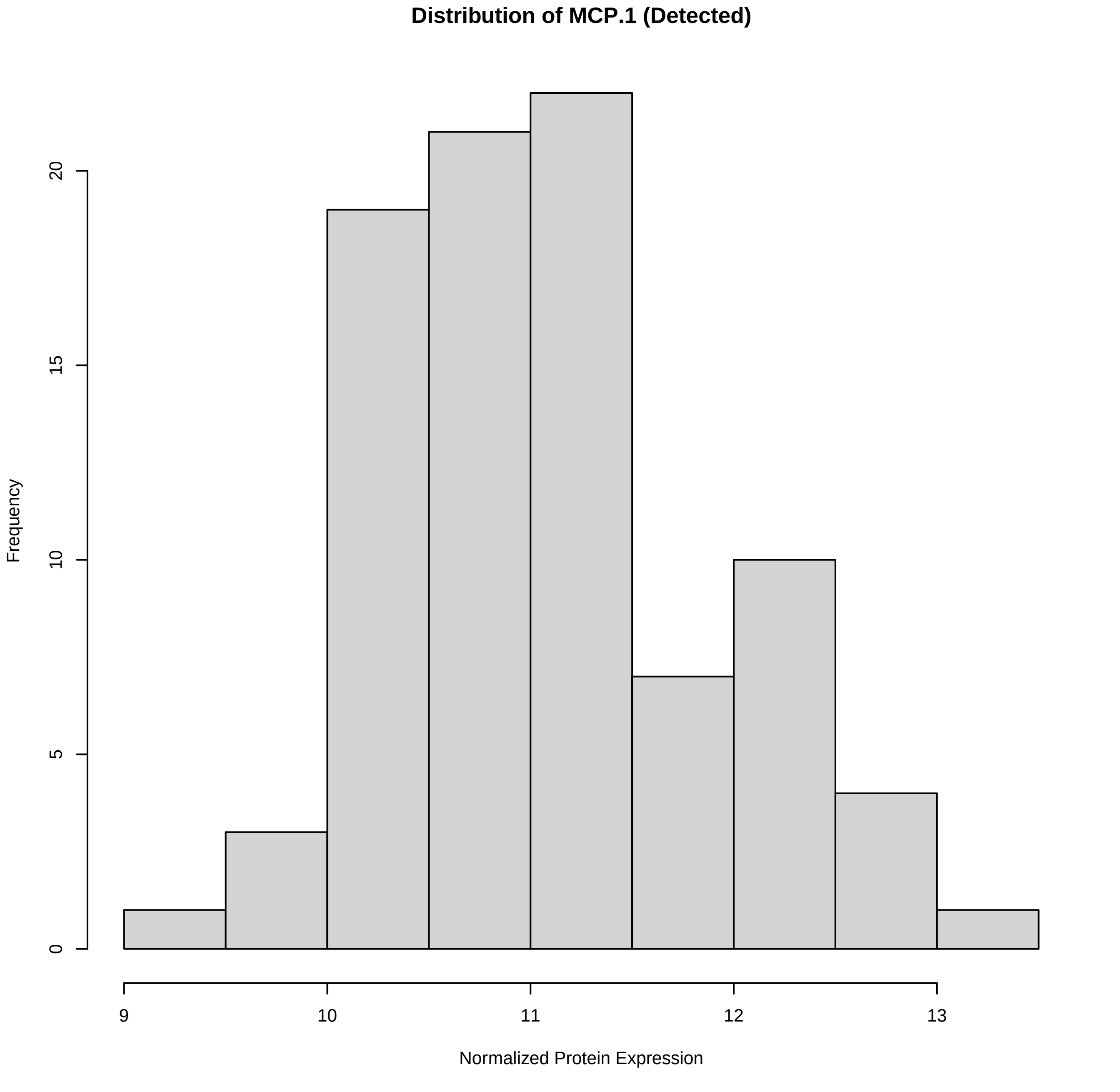
Distribution of MCP.1 (Detected)
Frequency
0
5
10
15
20
9
10
11
12
13
Normalized Protein Expression

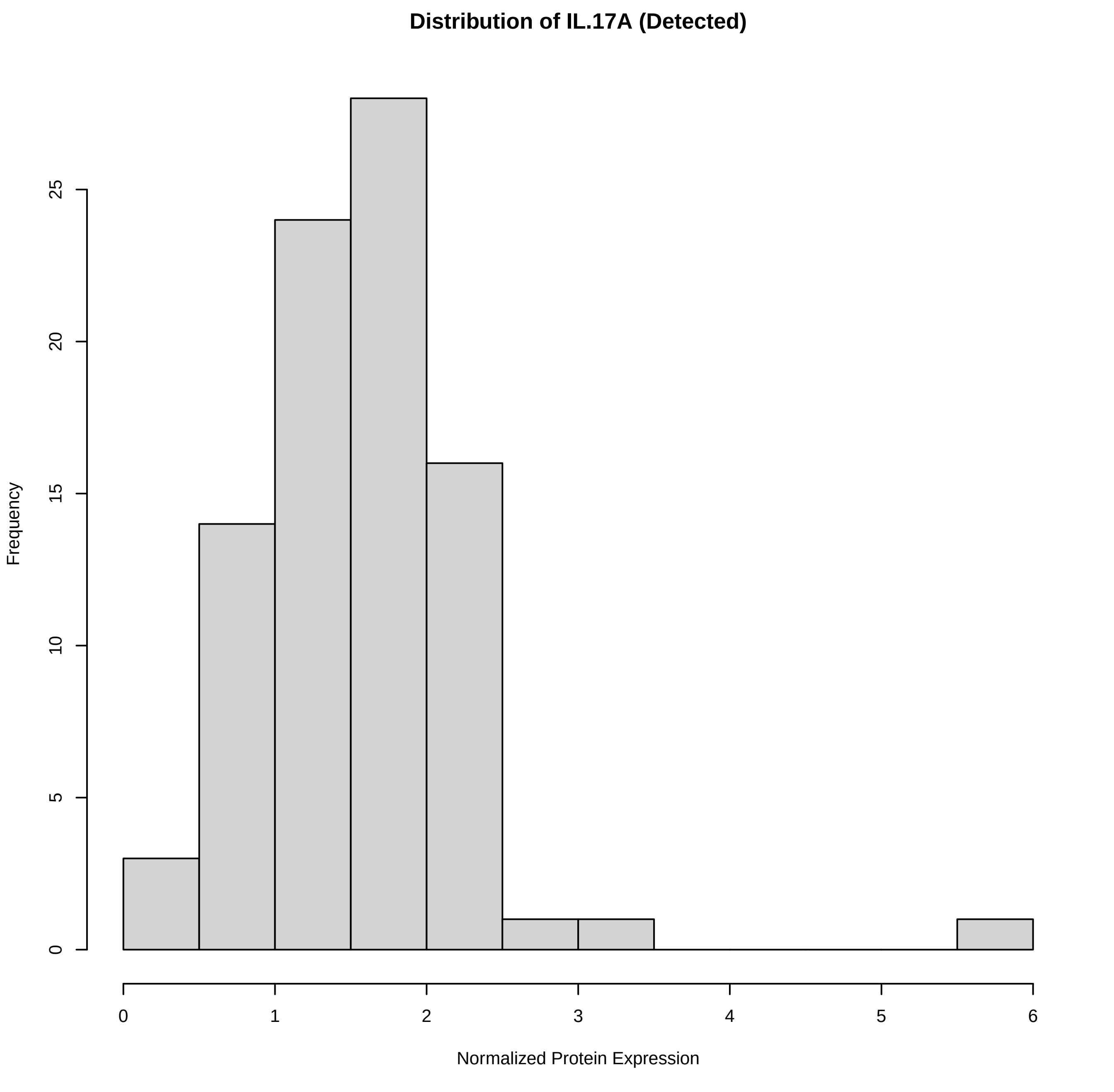

Distribution of IL.17A (Detected)
Frequency
0
5
10
15
20
25
0
1
2
3
4
5
6
Normalized Protein Expression

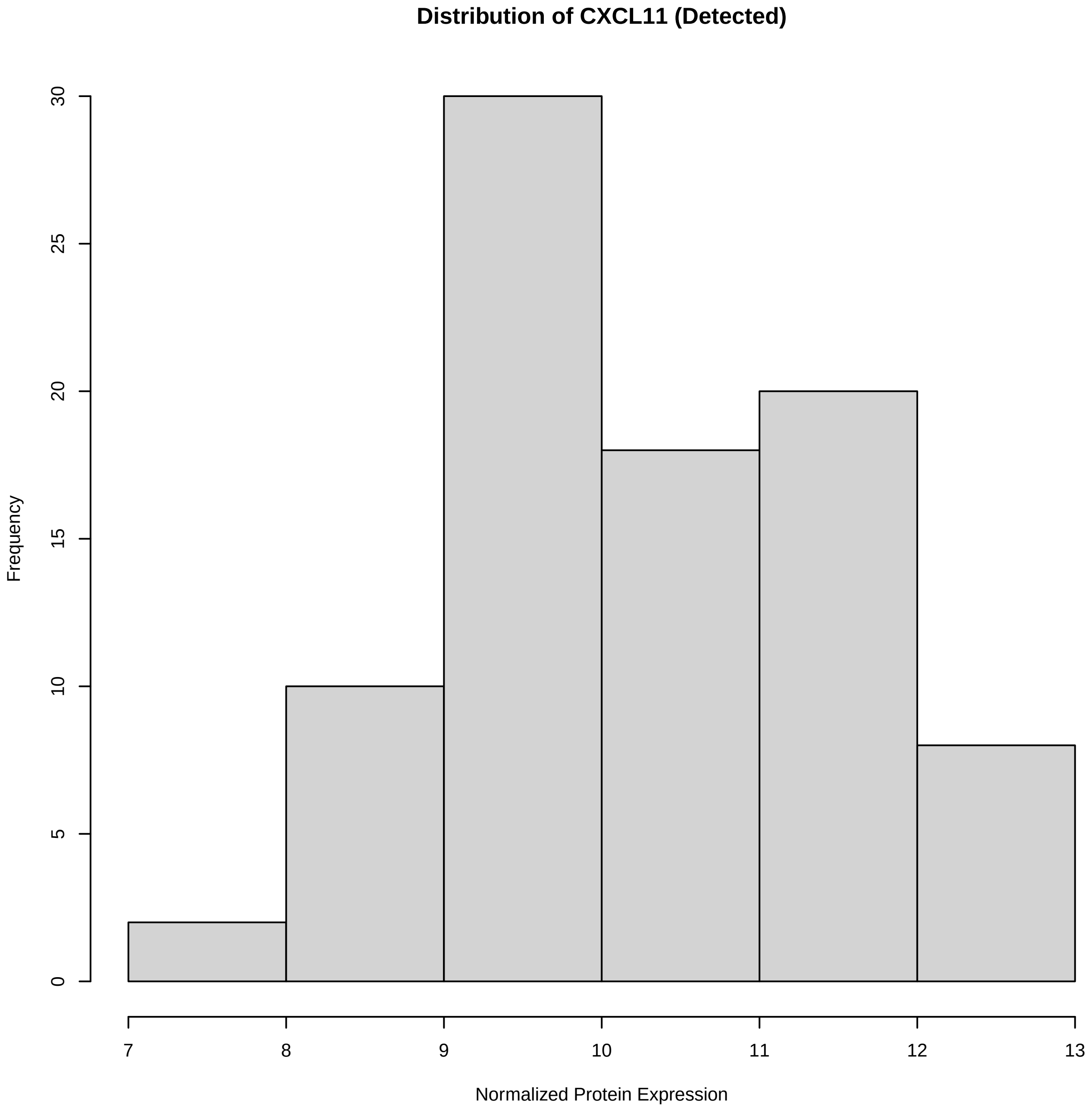

Distribution of CXCL11 (Detected)
Frequency
0
5
10
15
20
25
30
7
8
9
10
11
12
13
Normalized Protein Expression

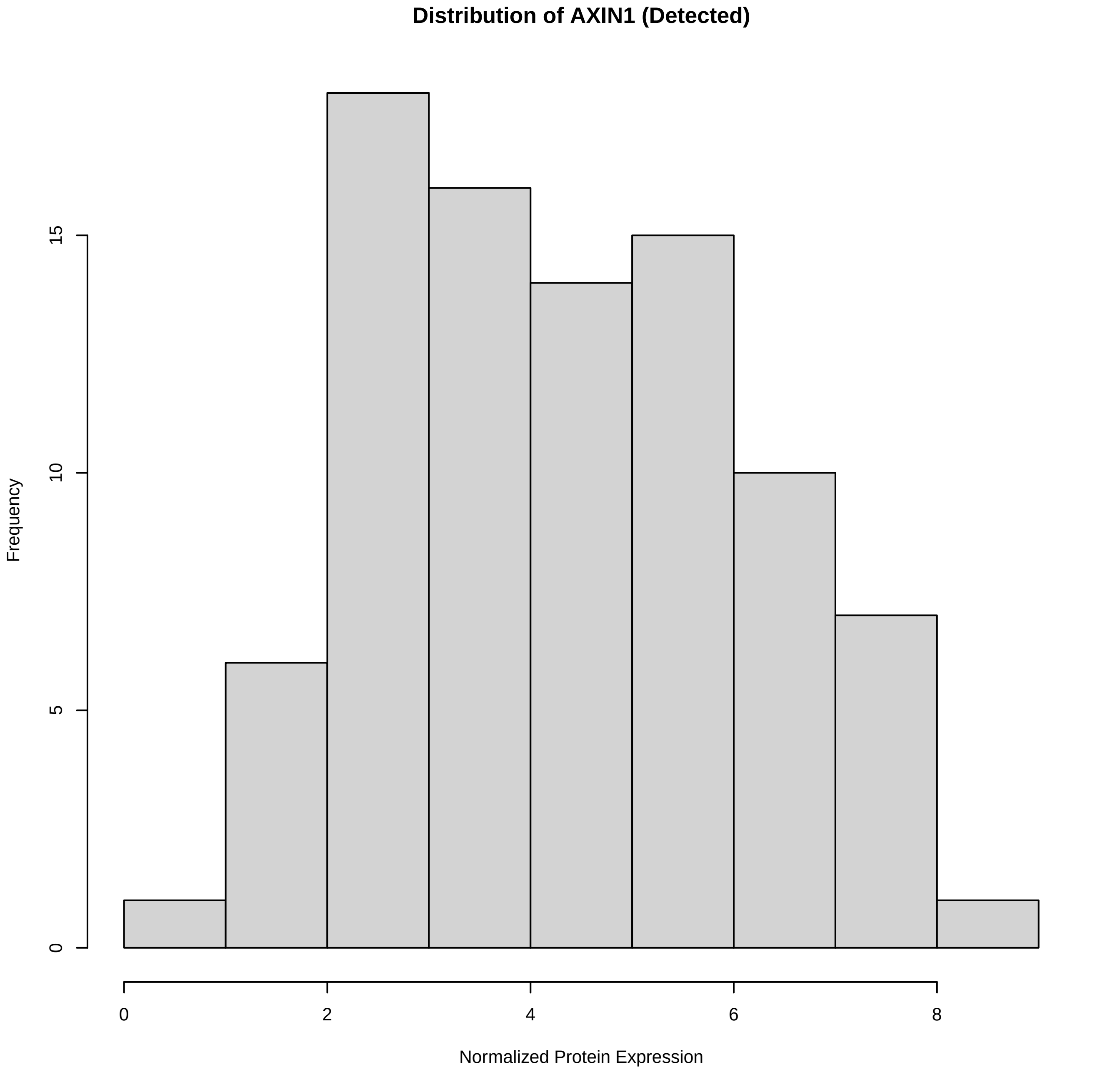

Distribution of AXIN1 (Detected)
Frequency
0
5
10
15
0
2
4
6
8
Normalized Protein Expression

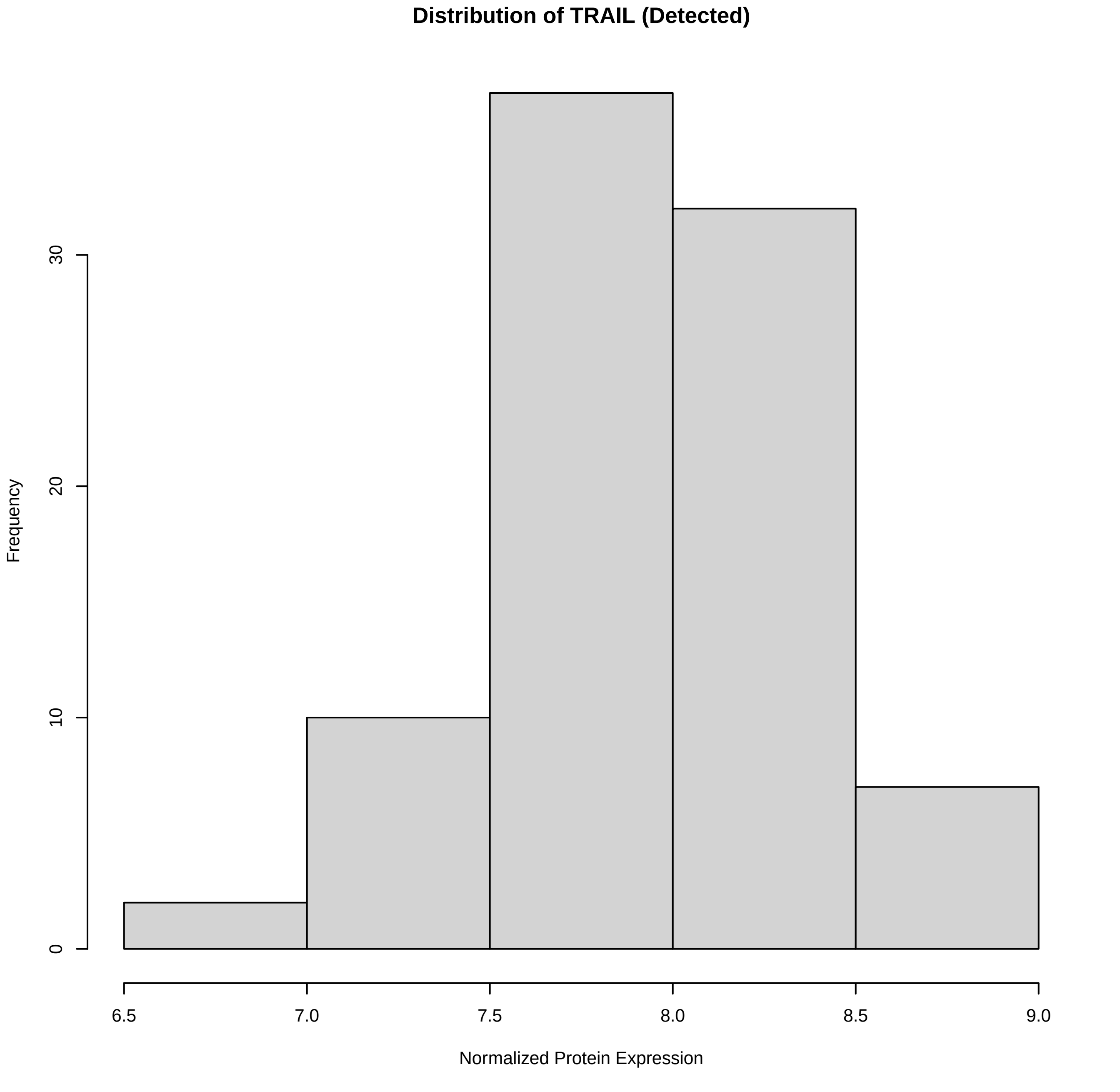
Distribution of TRAIL (Detected)
Frequency
0
10
20
30
6.5
7.0
7.5
8.0
8.5
9.0
Normalized Protein Expression

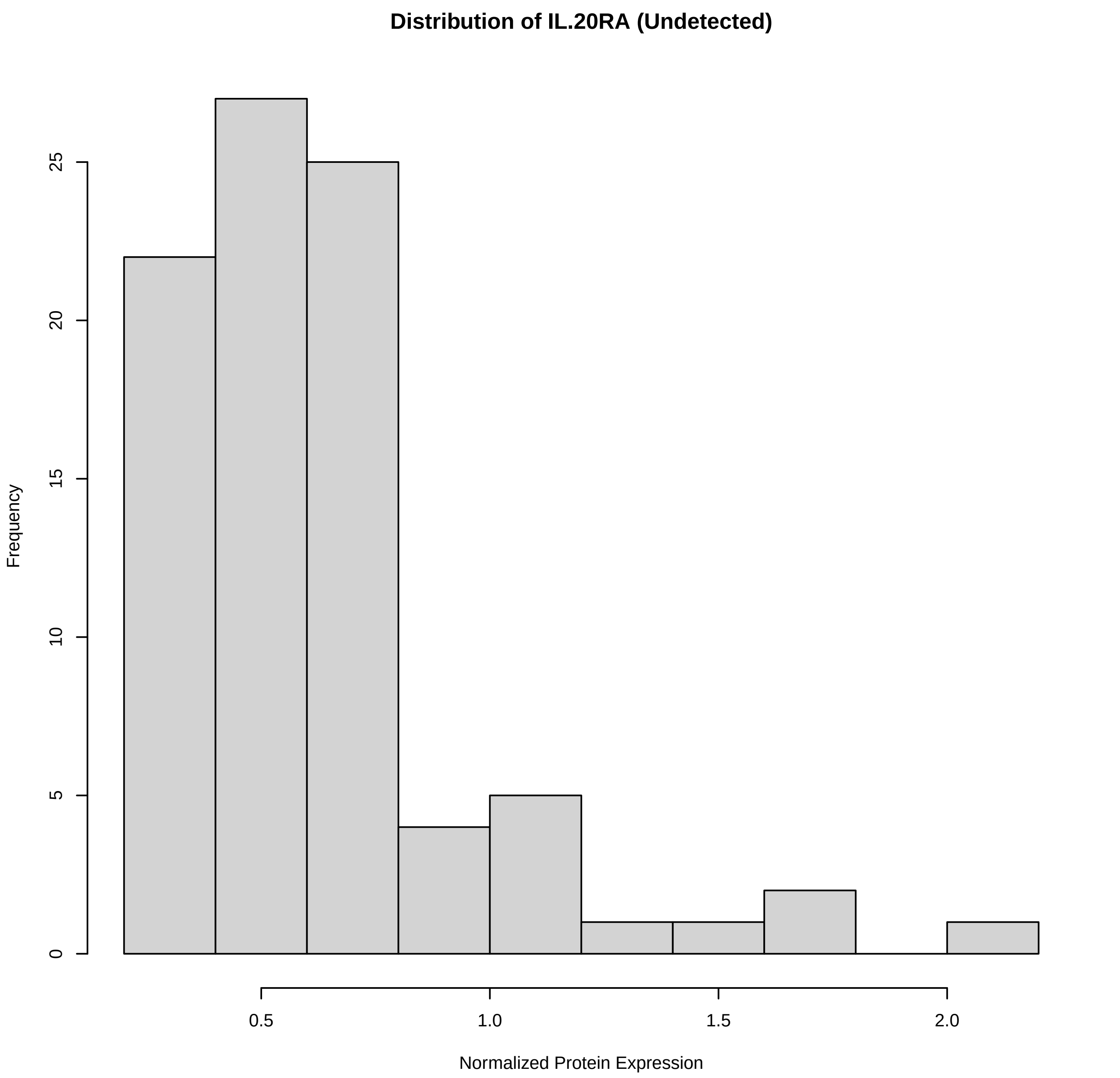

Distribution of IL.20RA (Undetected)
Frequency
0
5
10
15
20
25
0.5
1.0
1.5
2.0
Normalized Protein Expression

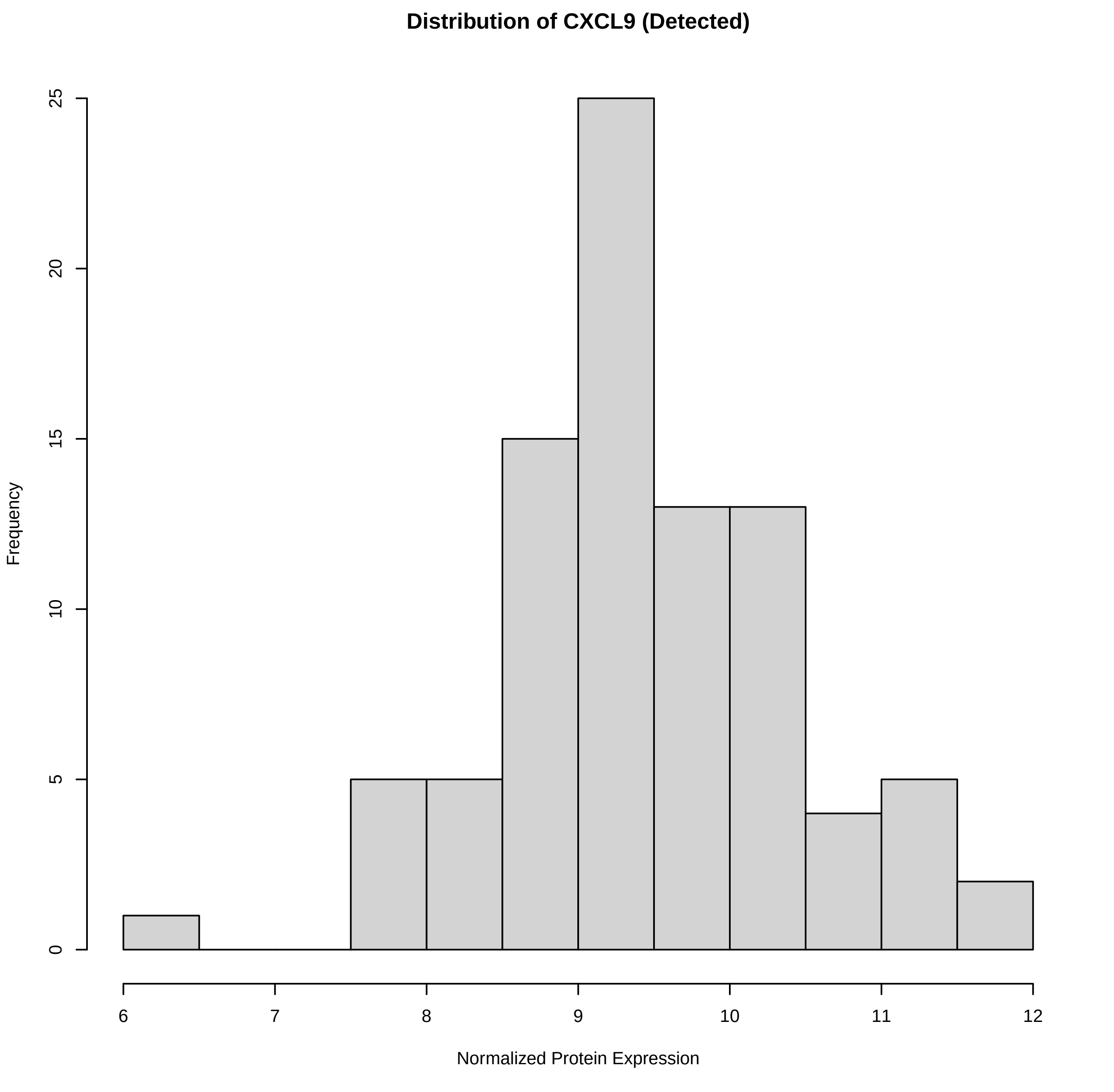
Distribution of CXCL9 (Detected)
Frequency
0
5
10
15
20
25
6
7
8
9
10
11
12
Normalized Protein Expression

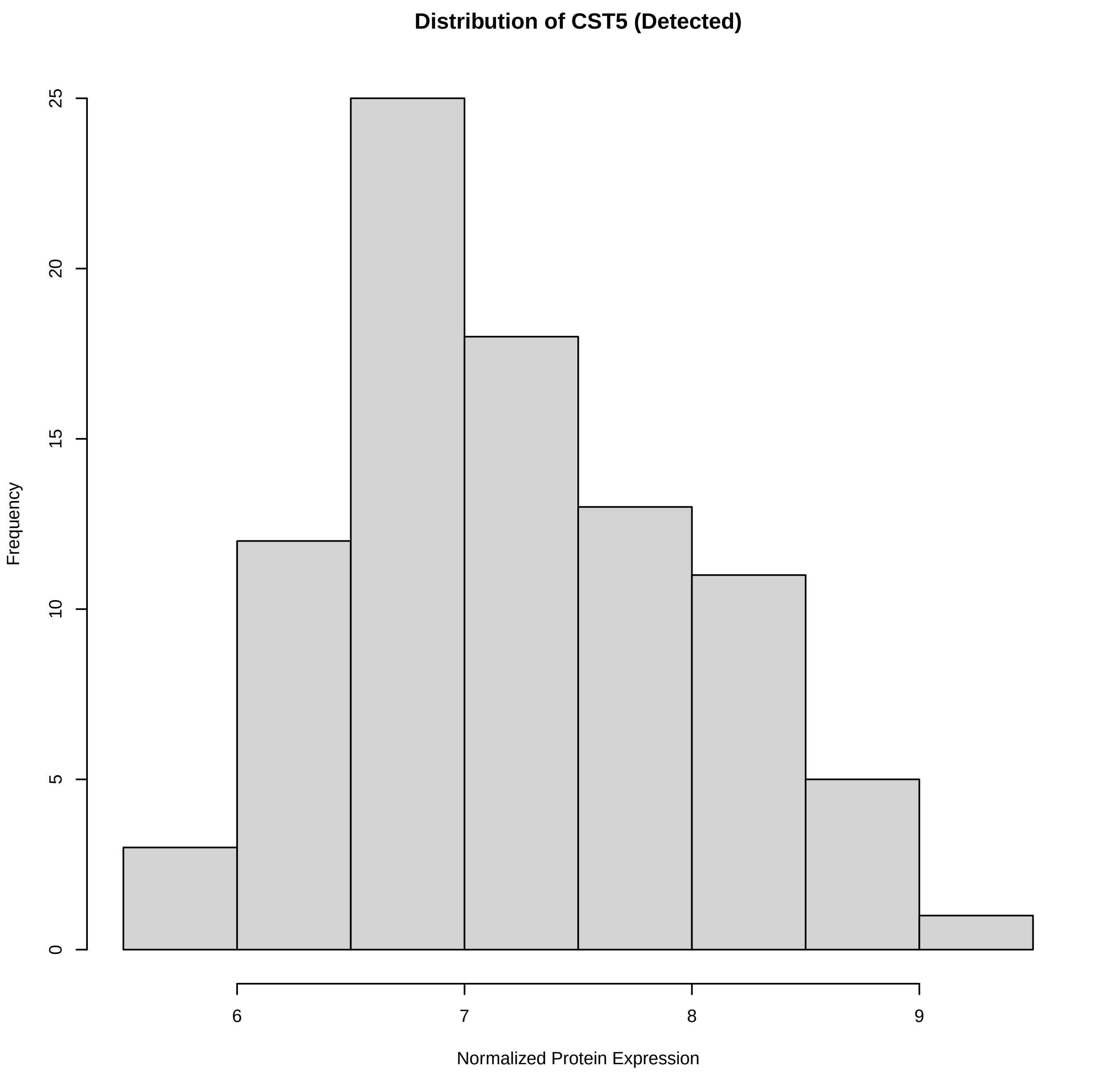

Distribution of CST5 (Detected)
Frequency
0
5
10
15
20
25
6
7
8
9
Normalized Protein Expression

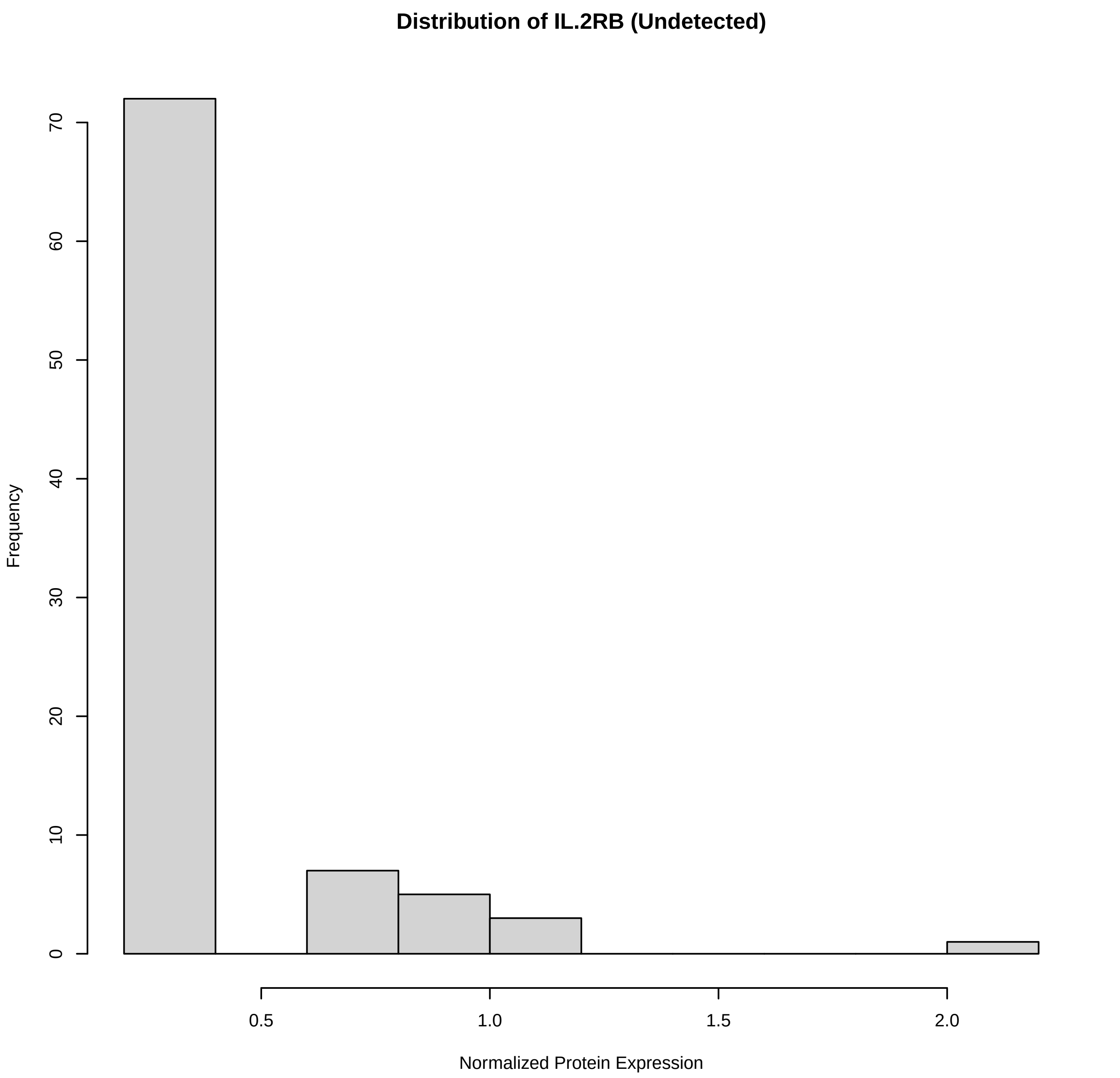

Distribution of IL.2RB (Undetected)
Frequency
0
10
20
30
40
50
60
70
0.5
1.0
1.5
2.0
Normalized Protein Expression

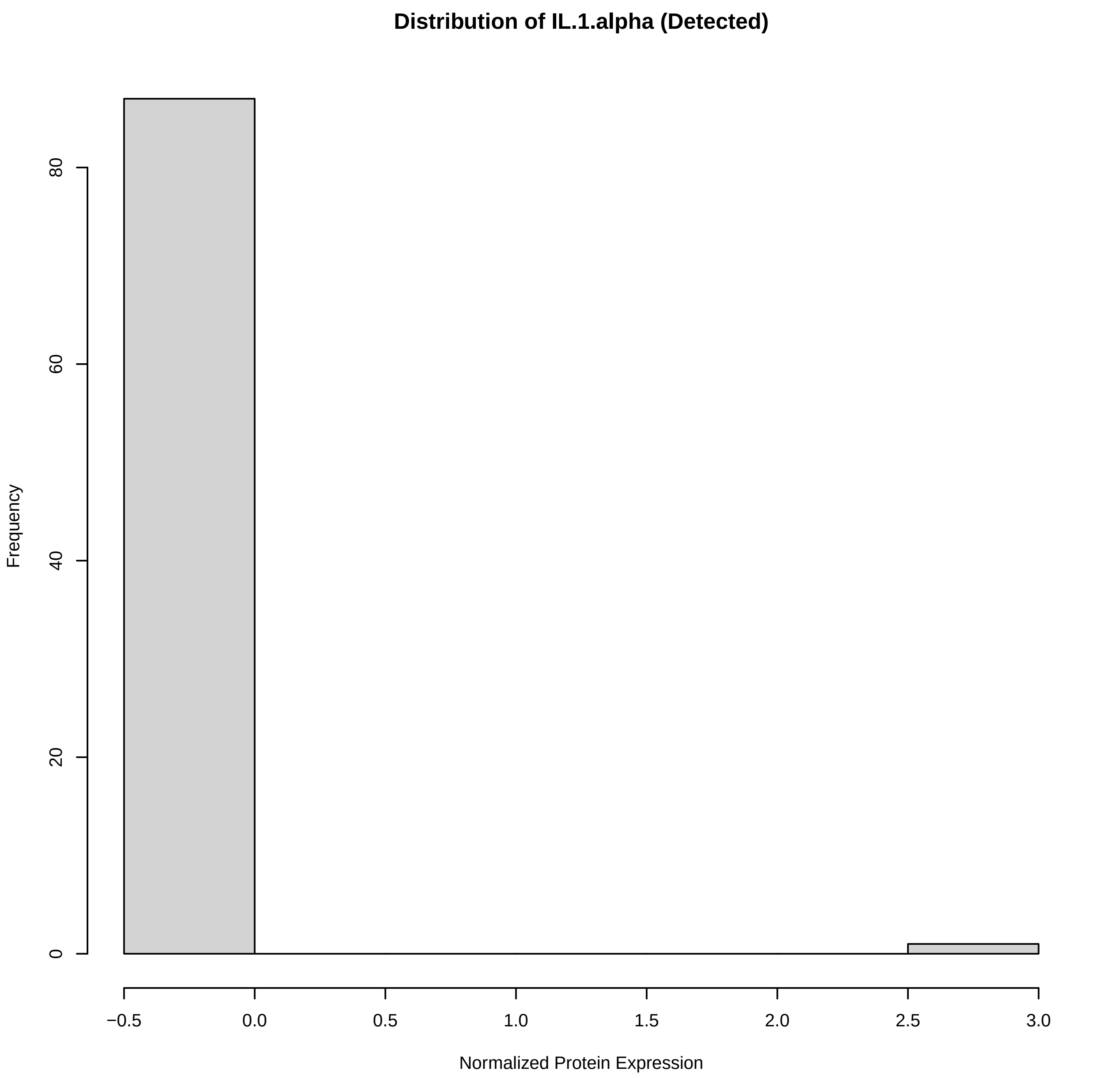
Distribution of IL.1.alpha (Detected)
Frequency
0
20
40
60
80
−0.5
0.0
0.5
1.0
1.5
2.0
2.5
3.0
Normalized Protein Expression

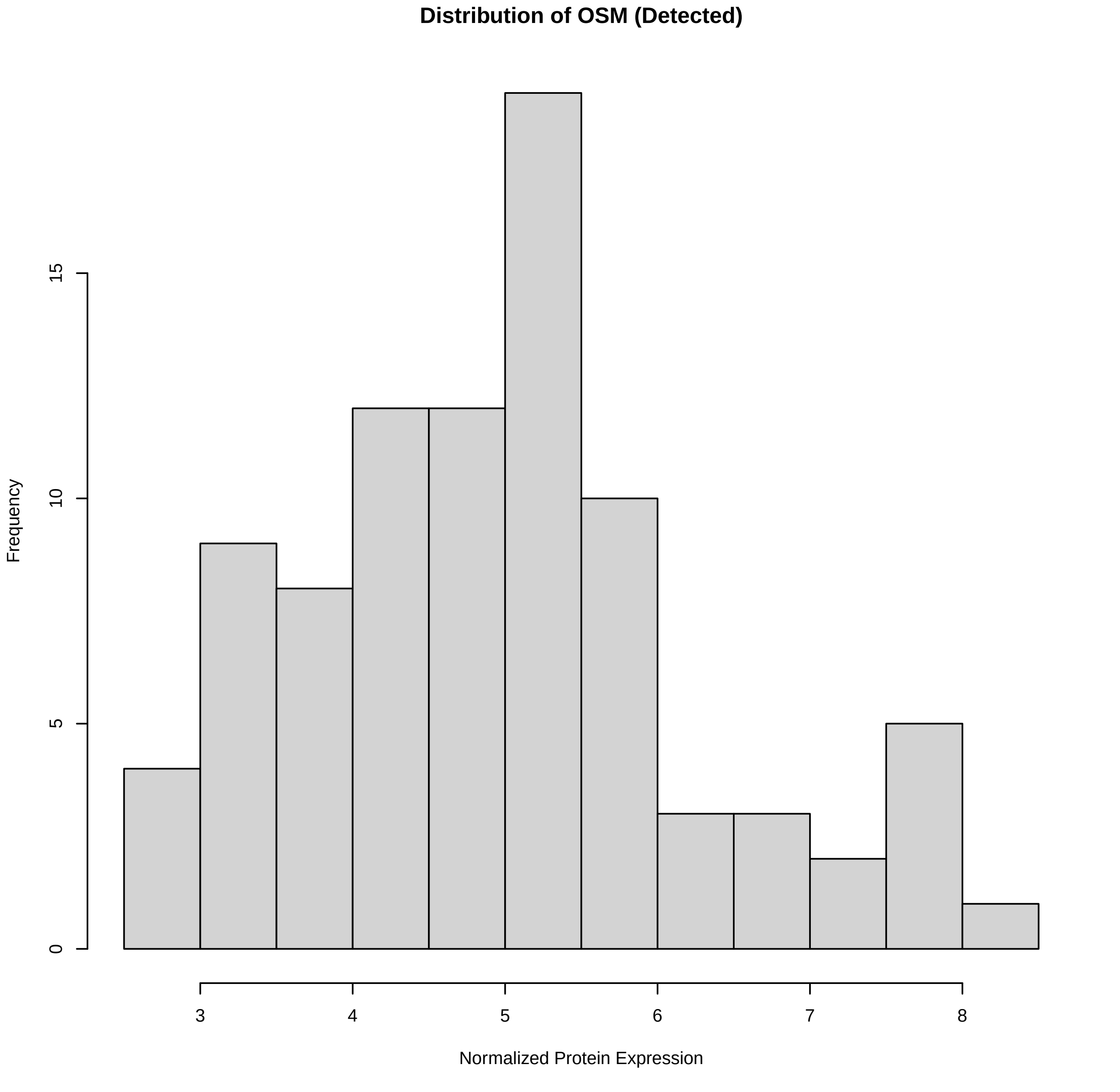
Distribution of OSM (Detected)
Frequency
0
5
10
15
3
4
5
6
7
8
Normalized Protein Expression

Distribution of IL2 (Undetected)

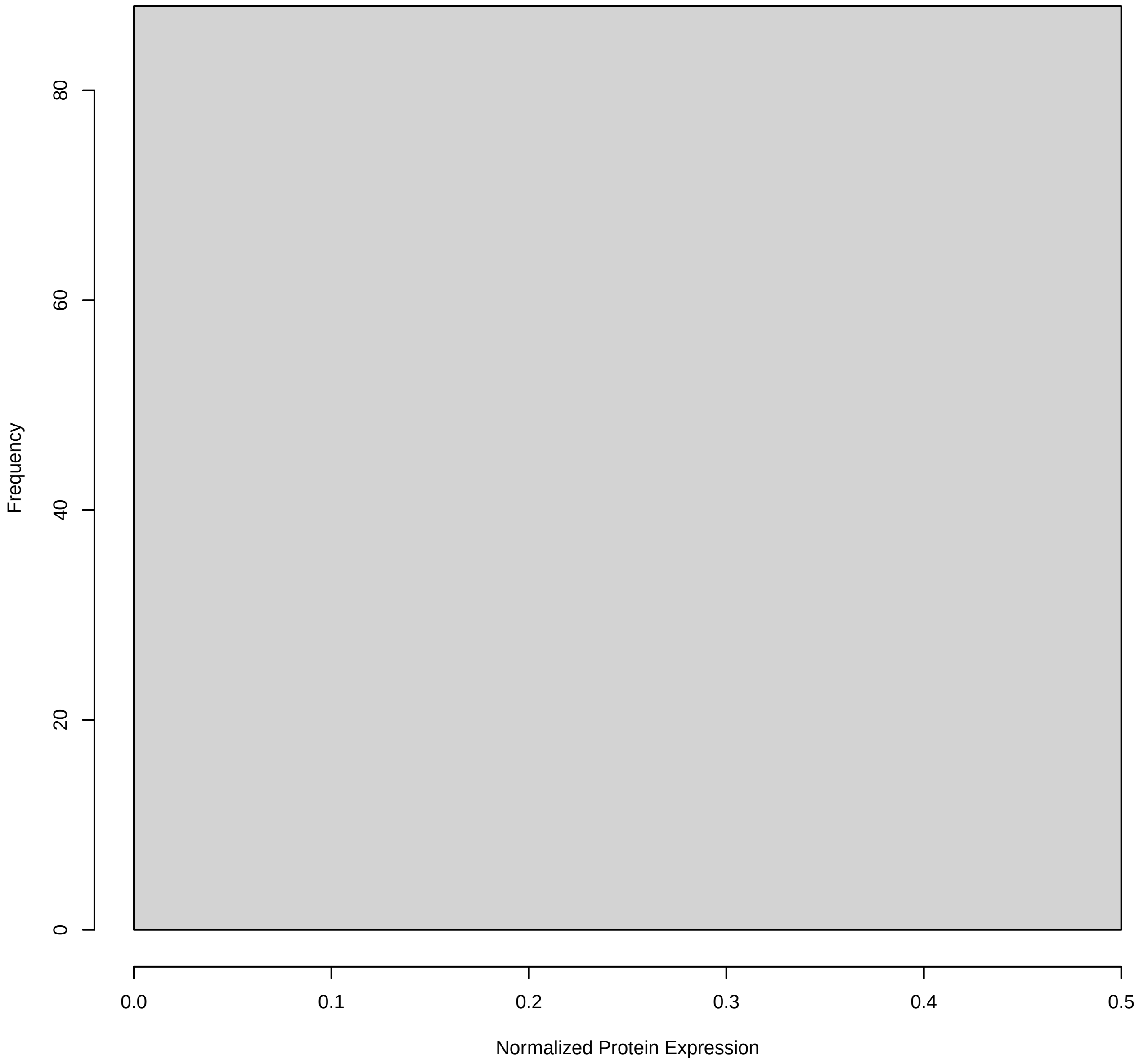

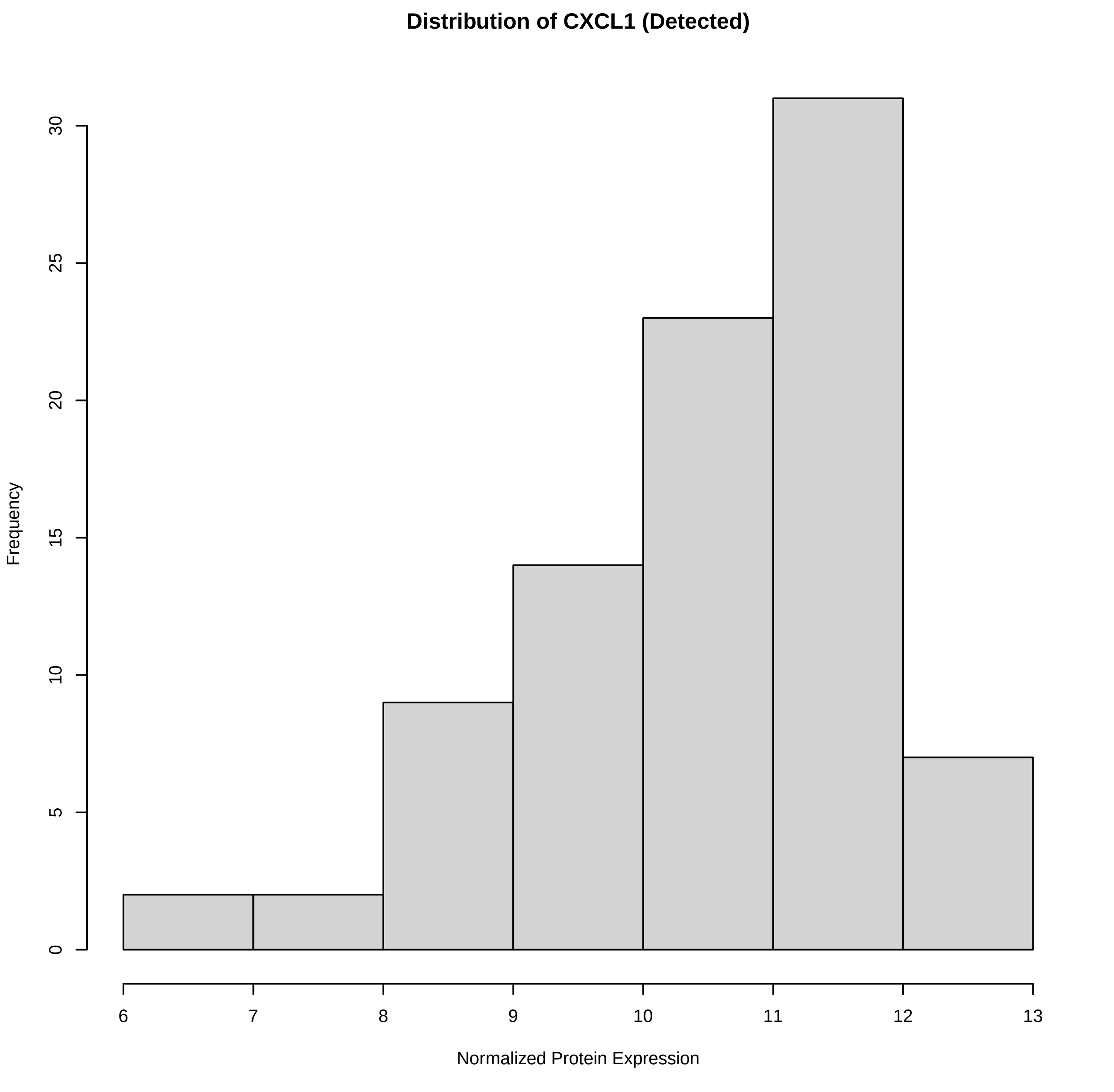
Distribution of CXCL1 (Detected)
Frequency
0
5
10
15
20
25
30
6
7
8
9
10
11
12
13
Normalized Protein Expression

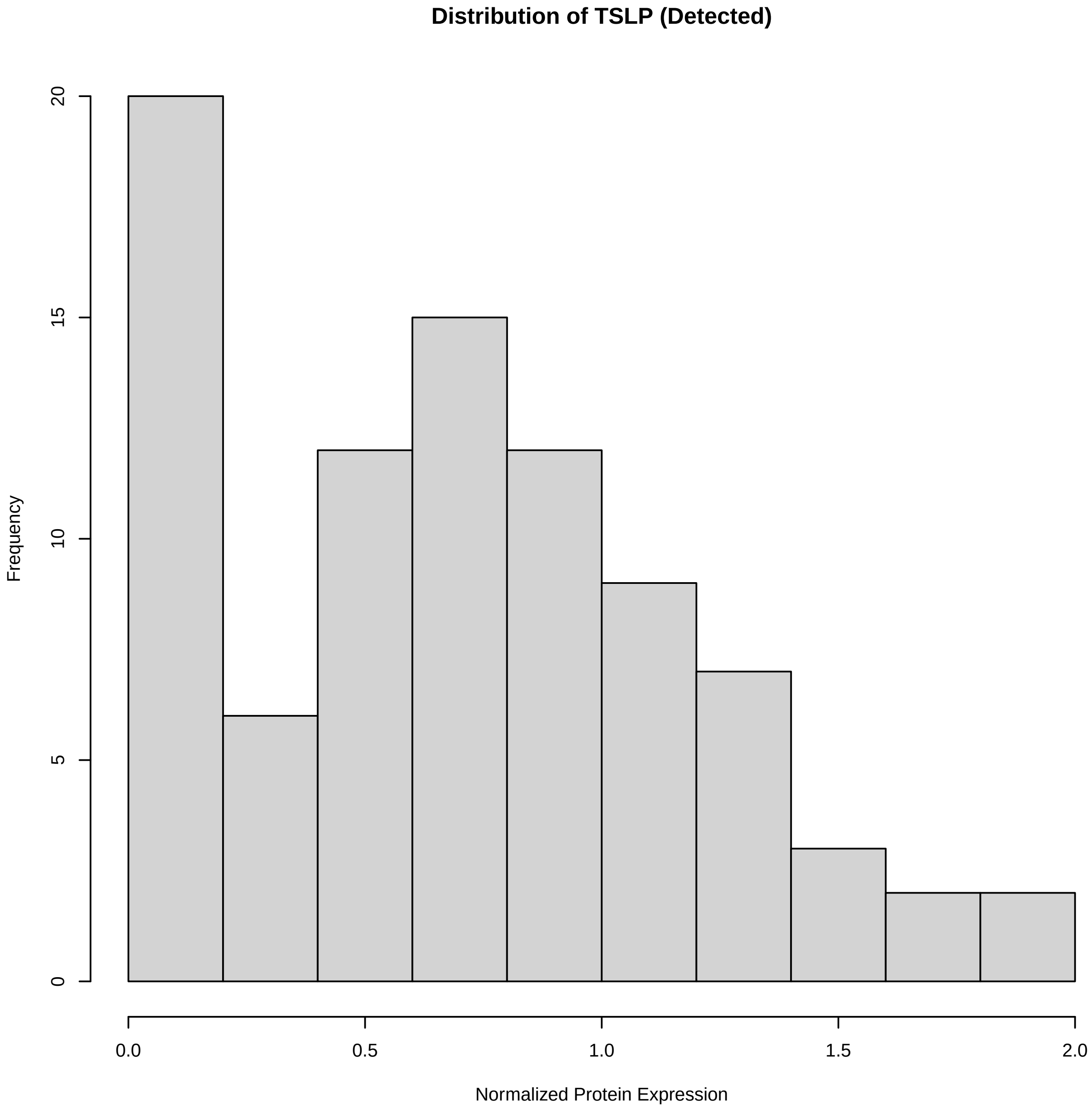

Distribution of TSLP (Detected)
Frequency
0
5
10
15
20
0.0
0.5
1.0
1.5
2.0
Normalized Protein Expression

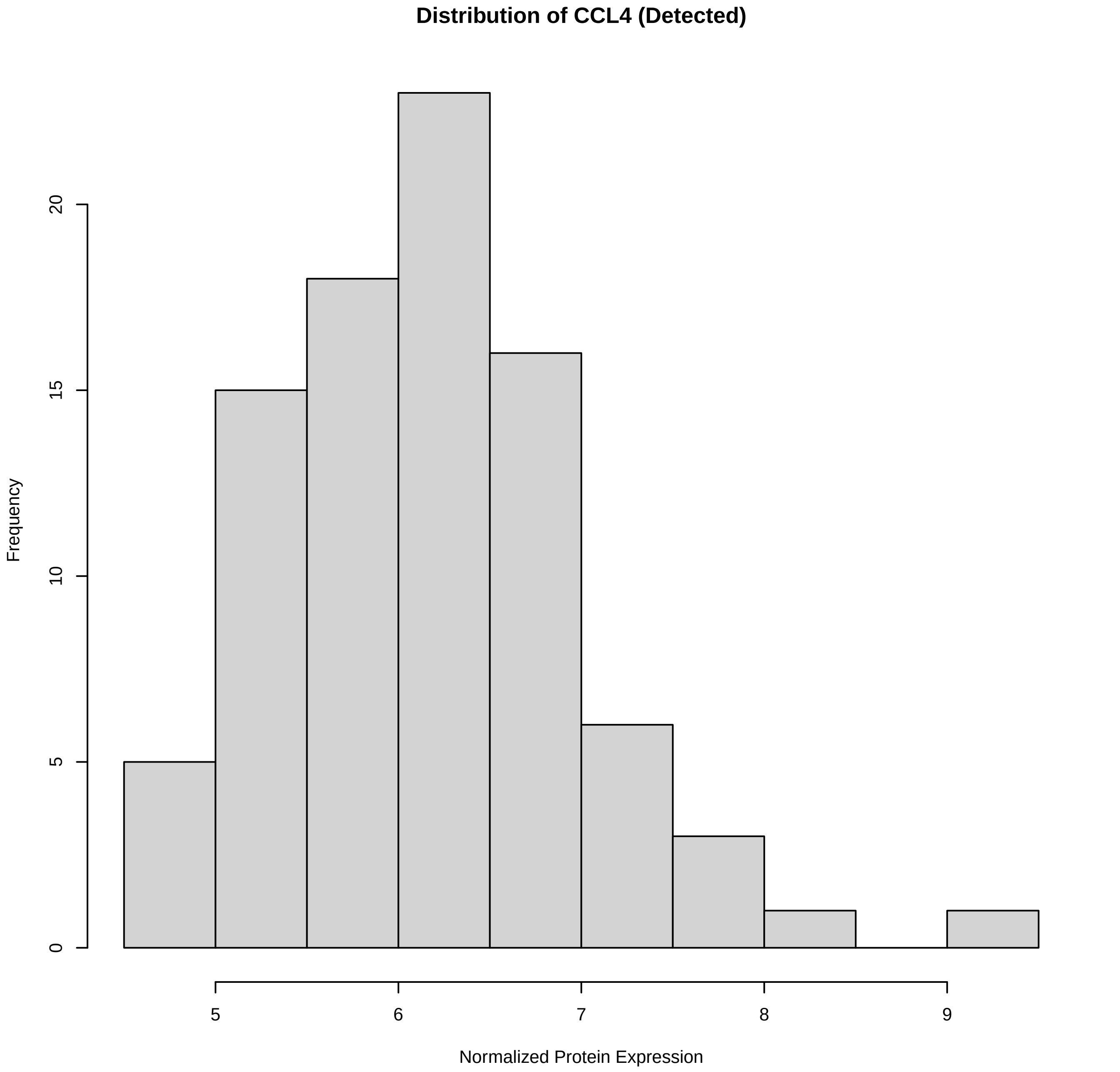
Distribution of CCL4 (Detected)
Frequency
0
5
10
15
20
5
6
7
8
9
Normalized Protein Expression

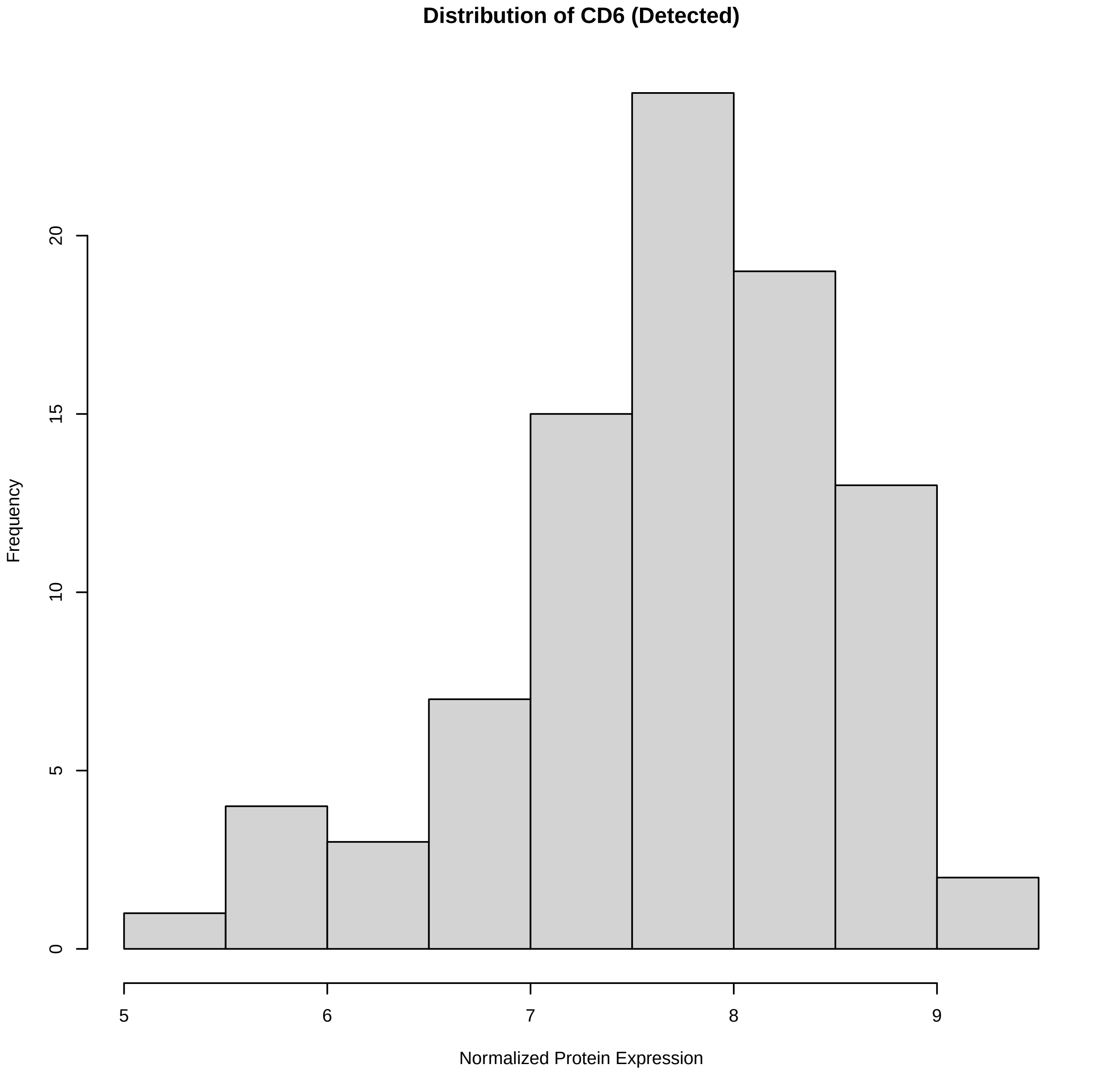

Distribution of CD6 (Detected)
Frequency
0
5
10
15
20
5
6
7
8
9
Normalized Protein Expression

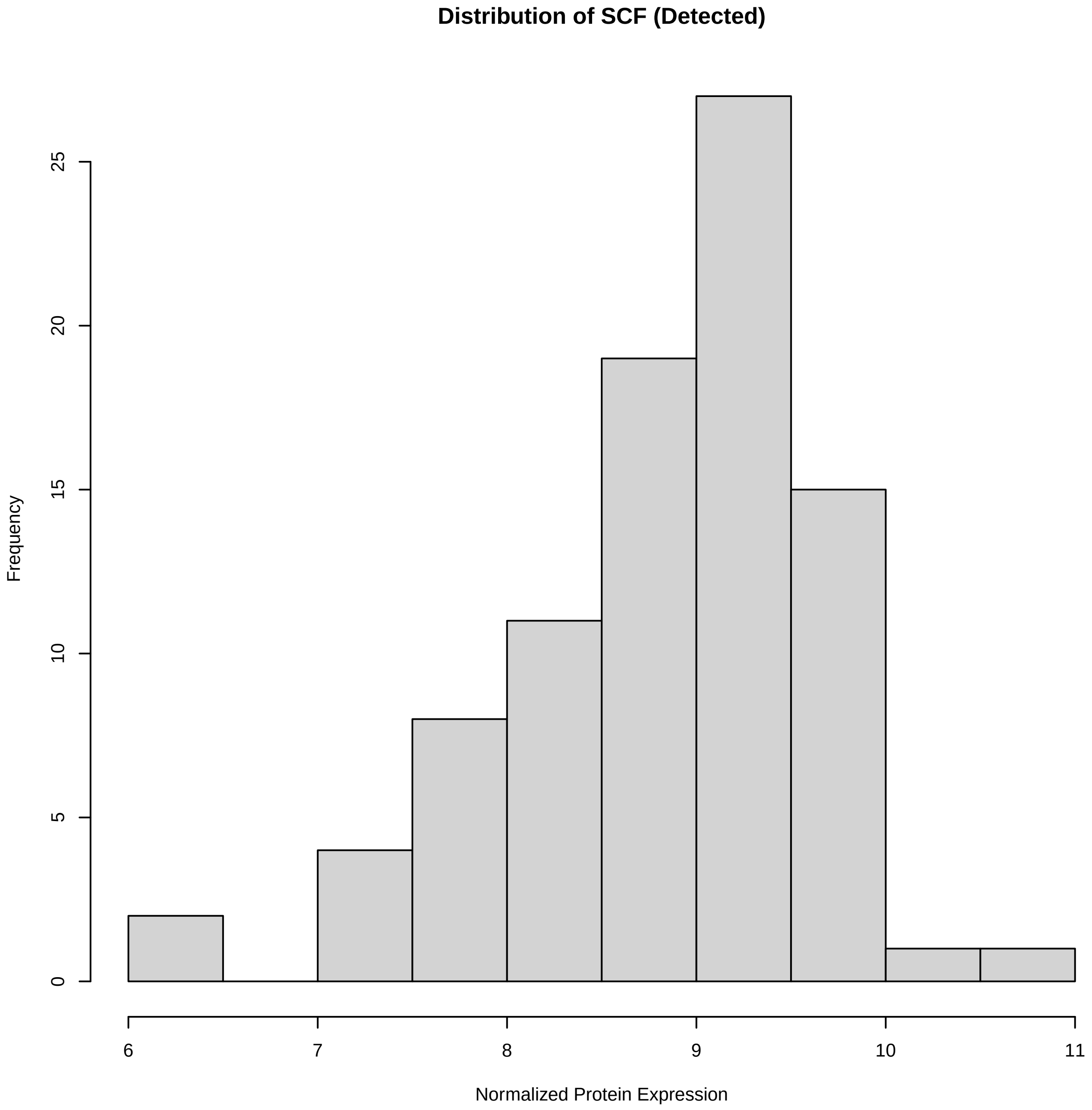

Distribution of SCF (Detected)
Frequency
0
5
10
15
20
25
6
7
8
9
10
11
Normalized Protein Expression

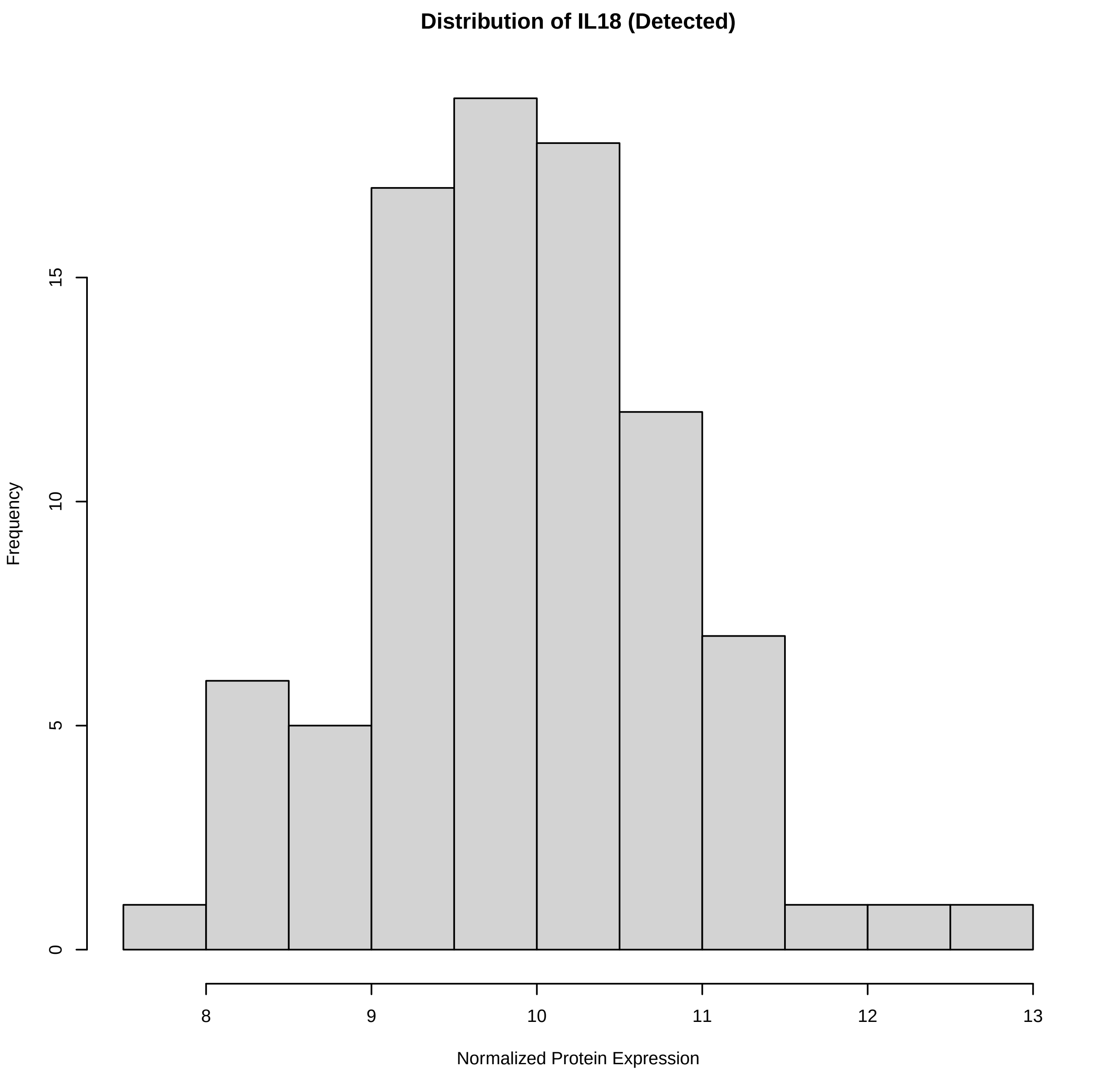
Distribution of IL18 (Detected)
Frequency
0
5
10
15
8
9
10
11
12
13
Normalized Protein Expression

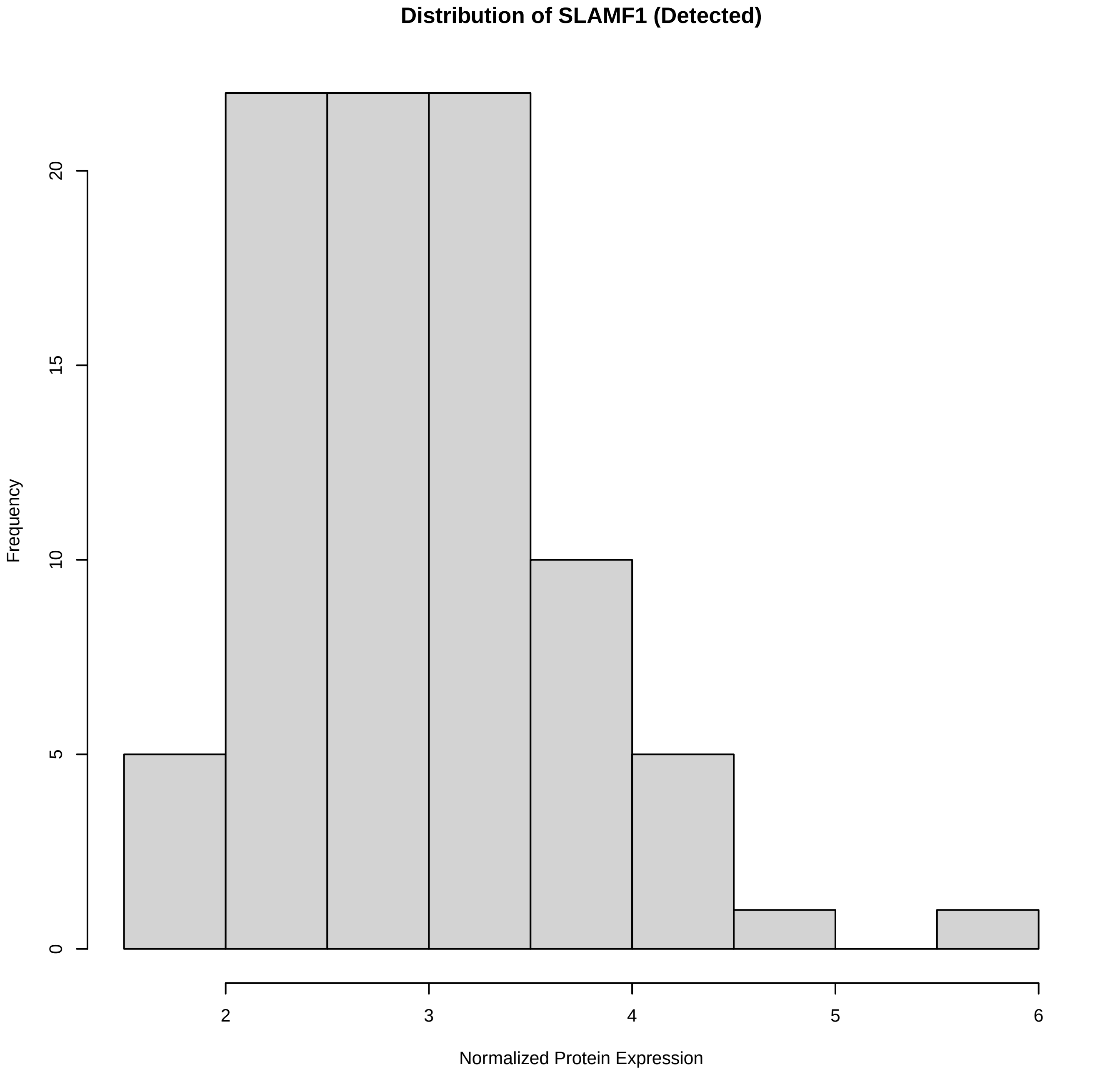

Distribution of SLAMF1 (Detected)
Frequency
0
5
10
15
20
2
3
4
5
6
Normalized Protein Expression

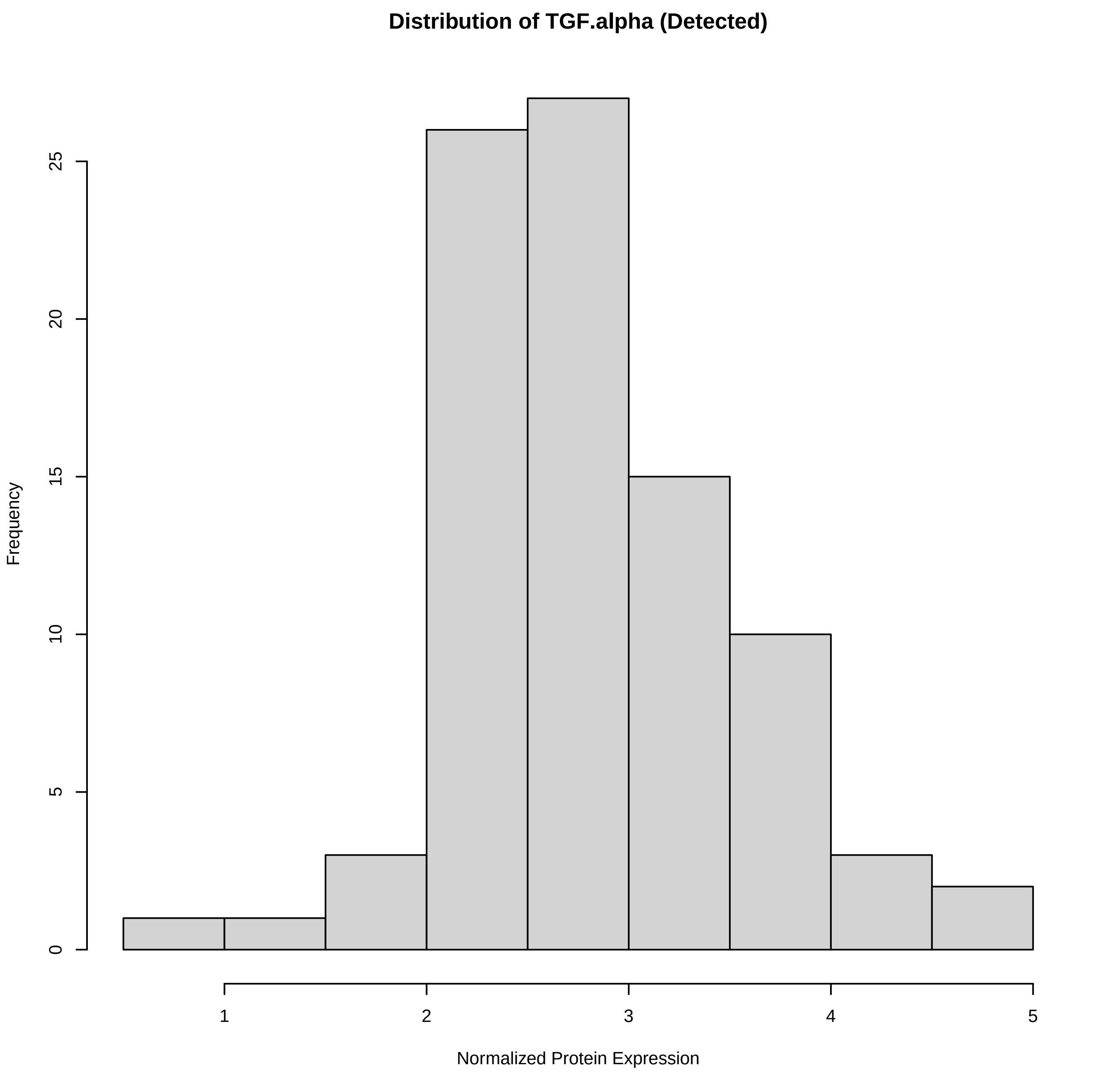

Distribution of TGF.alpha (Detected)
Frequency
0
5
10
15
20
25
1
2
3
4
5
Normalized Protein Expression

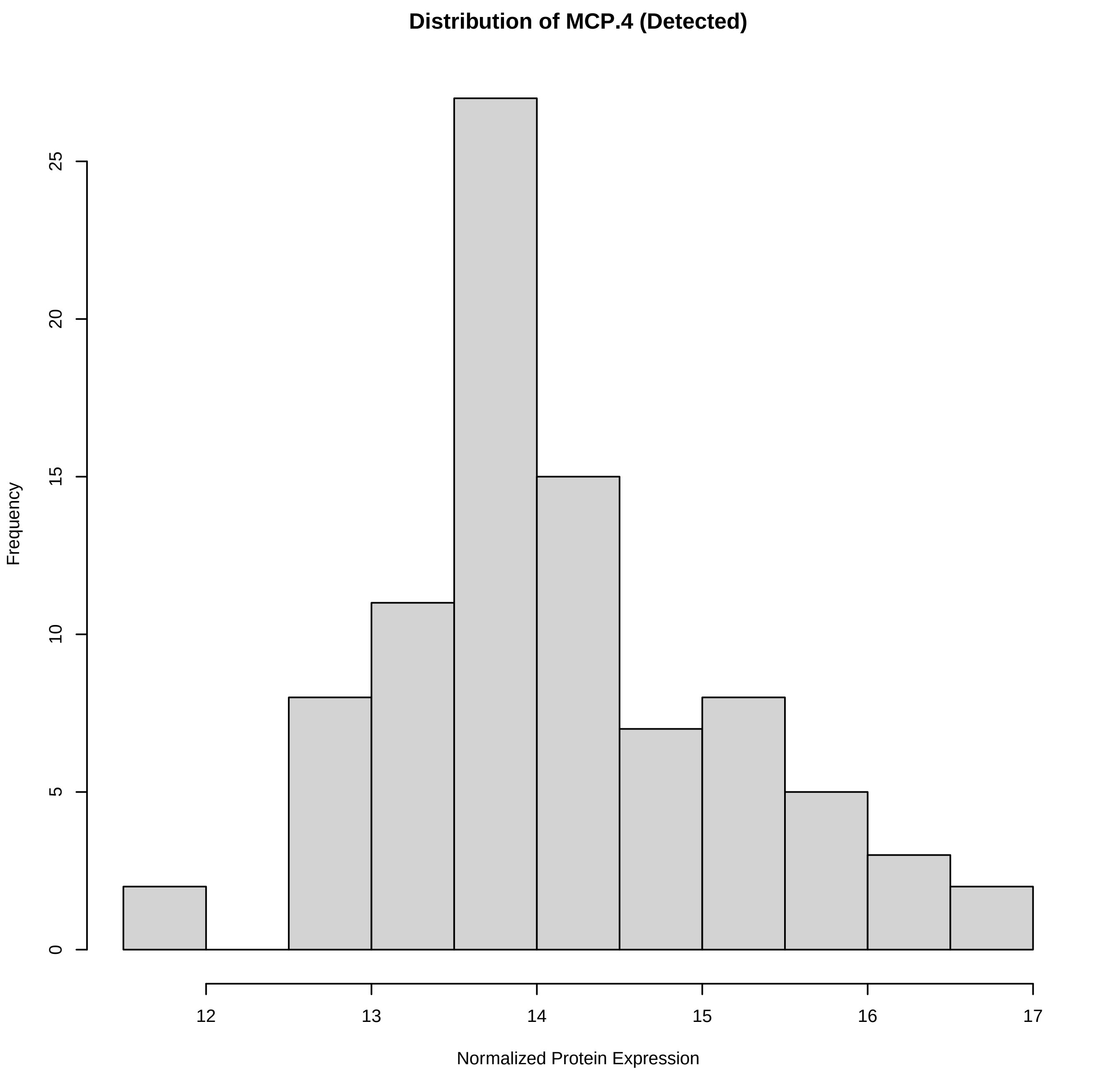
Distribution of MCP.4 (Detected)
Frequency
0
5
10
15
20
25
12
13
14
15
16
17
Normalized Protein Expression

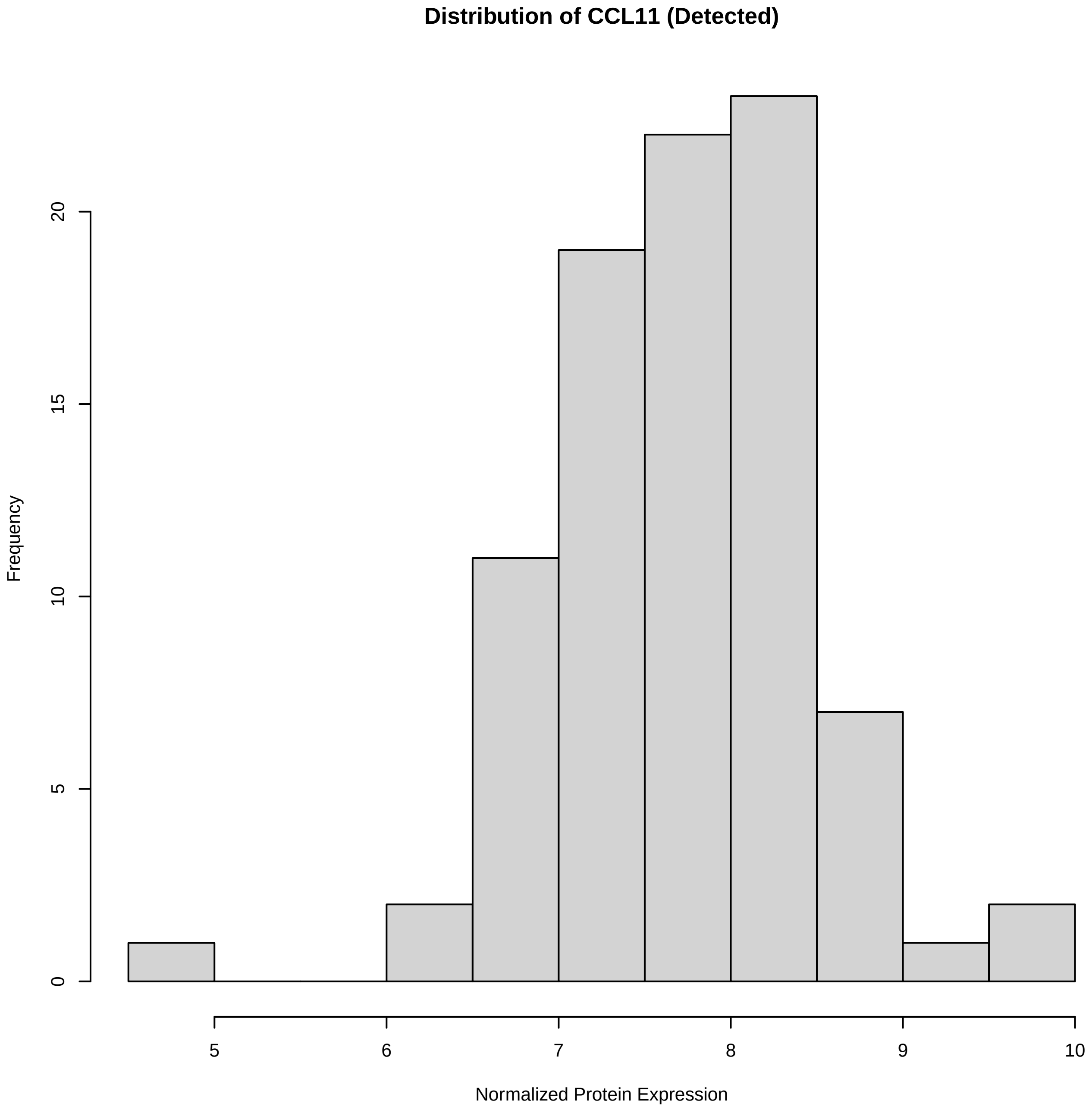
Distribution of CCL11 (Detected)
Frequency
0
5
10
15
20
5
6
7
8
9
10
Normalized Protein Expression

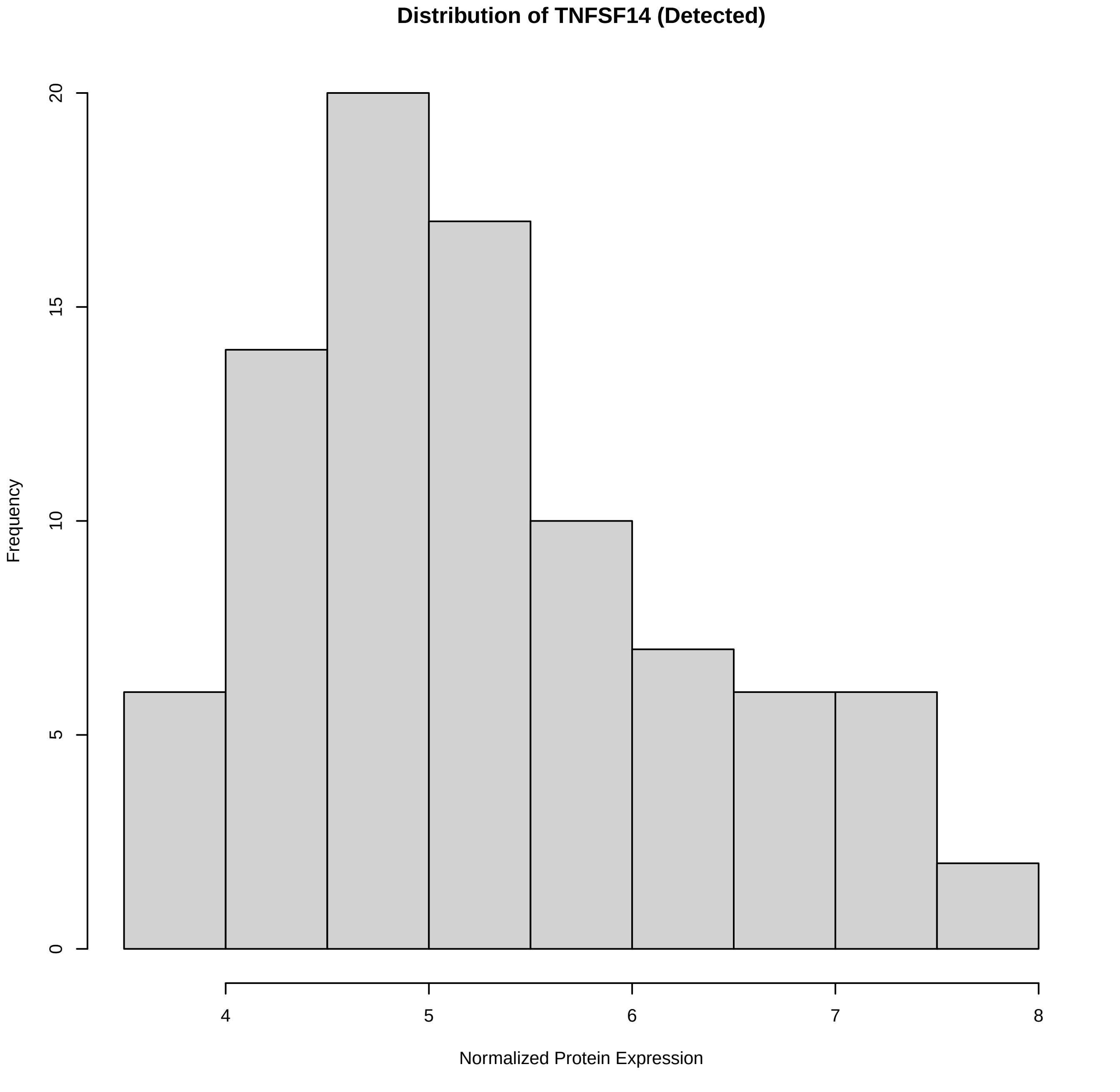
Distribution of TNFSF14 (Detected)
Frequency
0
5
10
15
20
4
5
6
7
8
Normalized Protein Expression

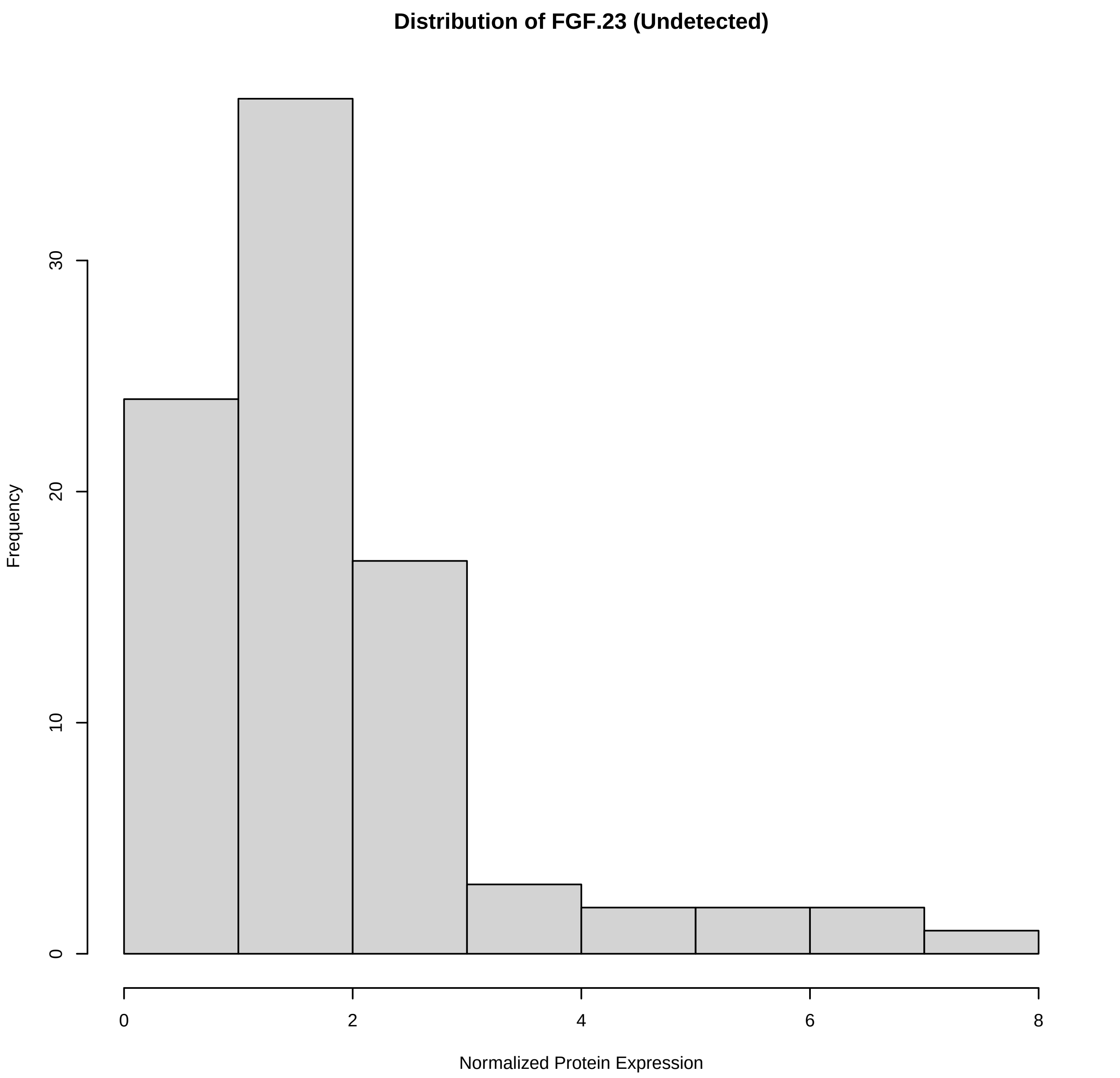

Distribution of FGF.23 (Undetected)
Frequency
0
10
20
30
0
2
4
6
8
Normalized Protein Expression

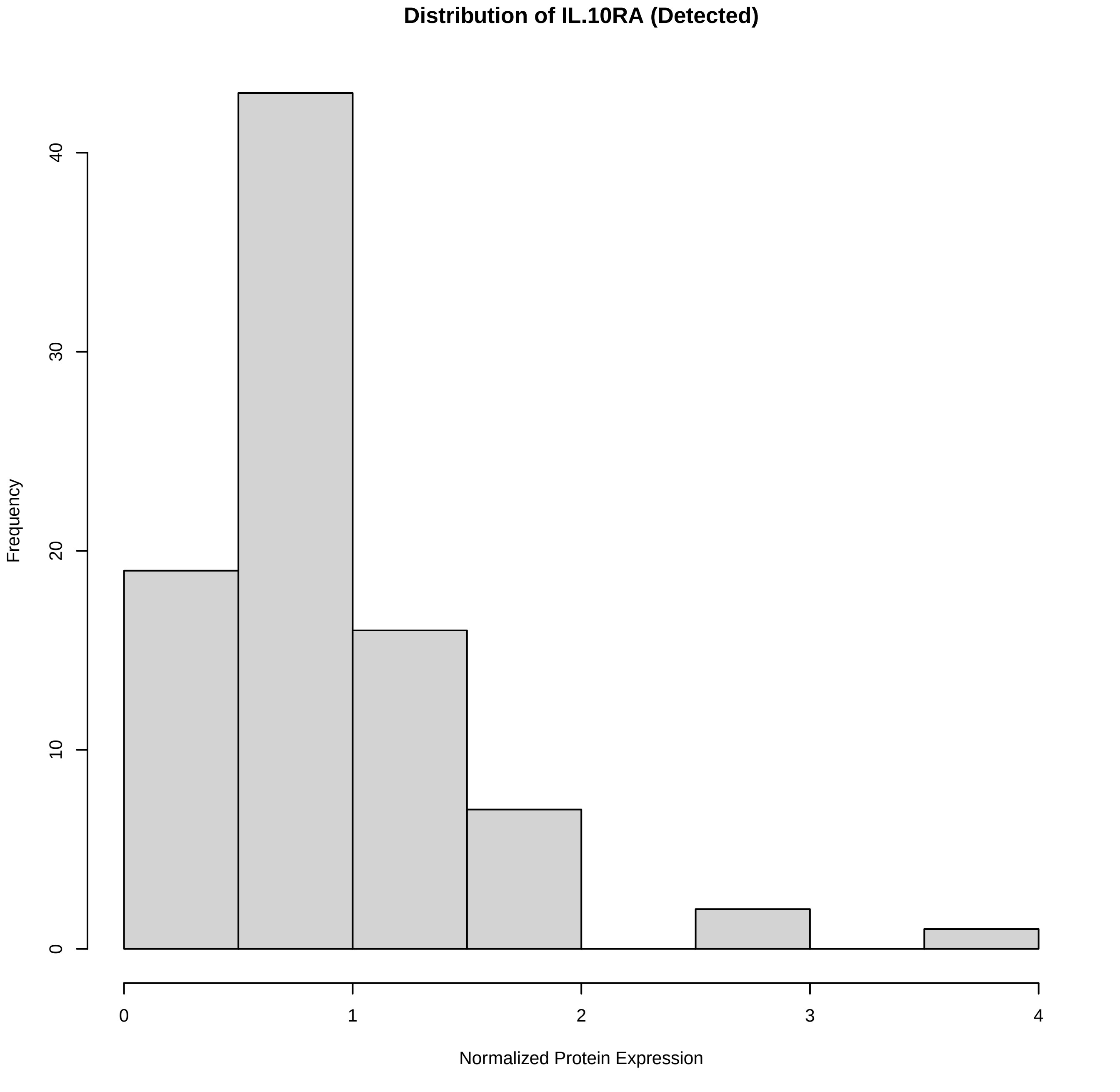
Distribution of IL.10RA (Detected)
Frequency
0
10
20
30
40
0
1
2
3
4
Normalized Protein Expression

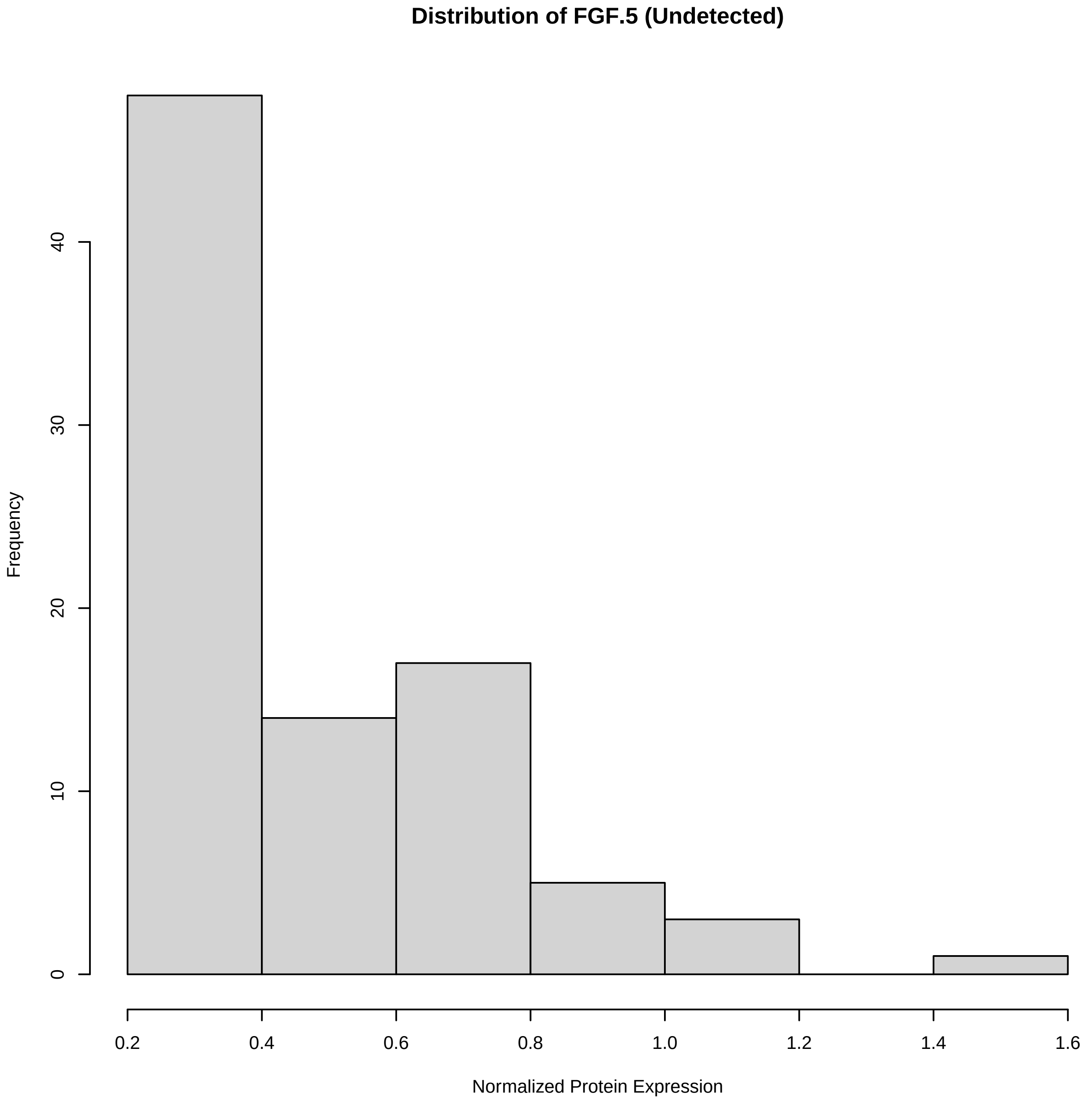

Distribution of FGF.5 (Undetected)
Frequency
0
10
20
30
40
0.2
0.4
0.6
0.8
1.0
1.2
1.4
1.6
Normalized Protein Expression

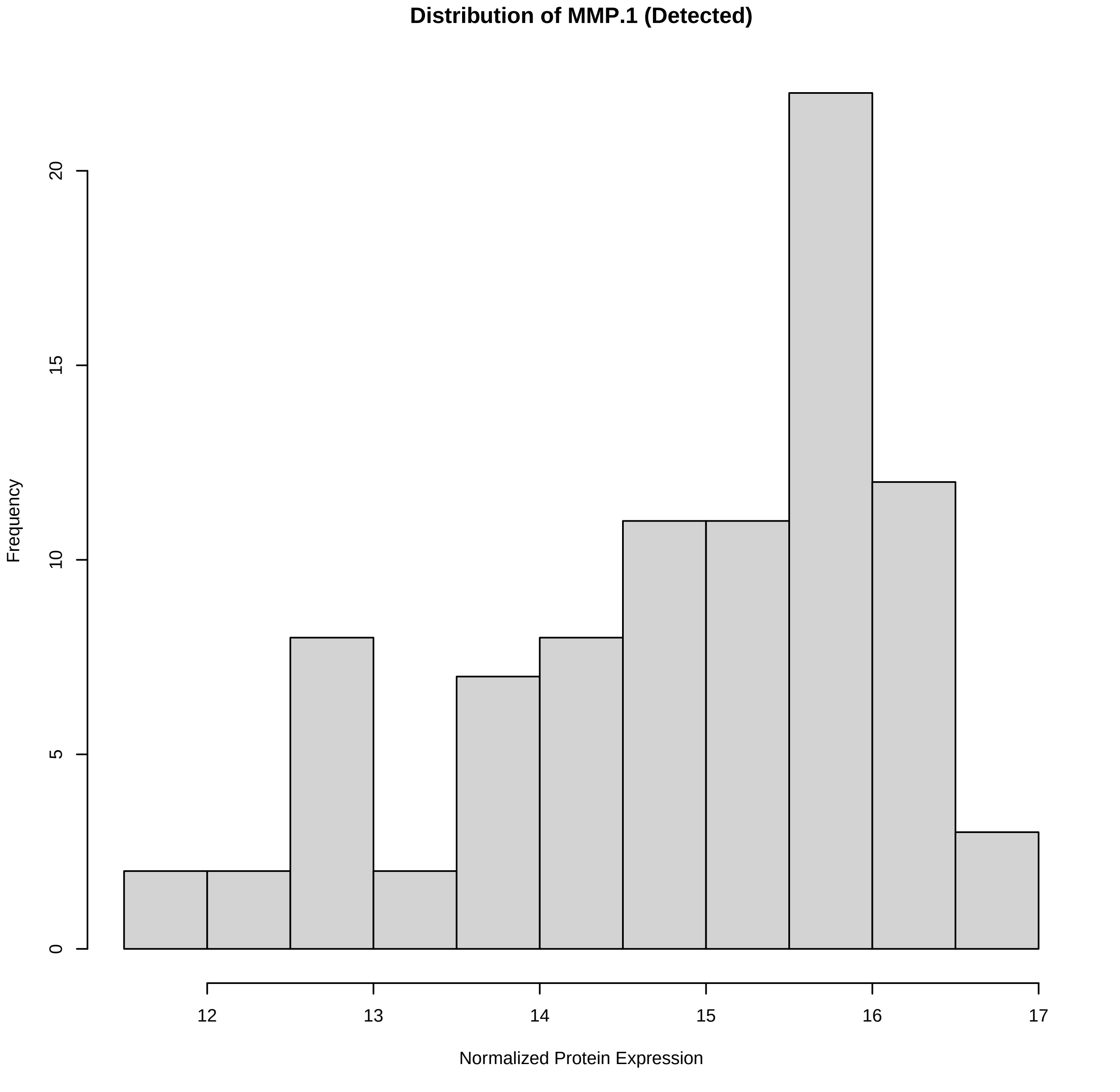

Distribution of MMP.1 (Detected)
Frequency
0
5
10
15
20
12
13
14
15
16
17
Normalized Protein Expression

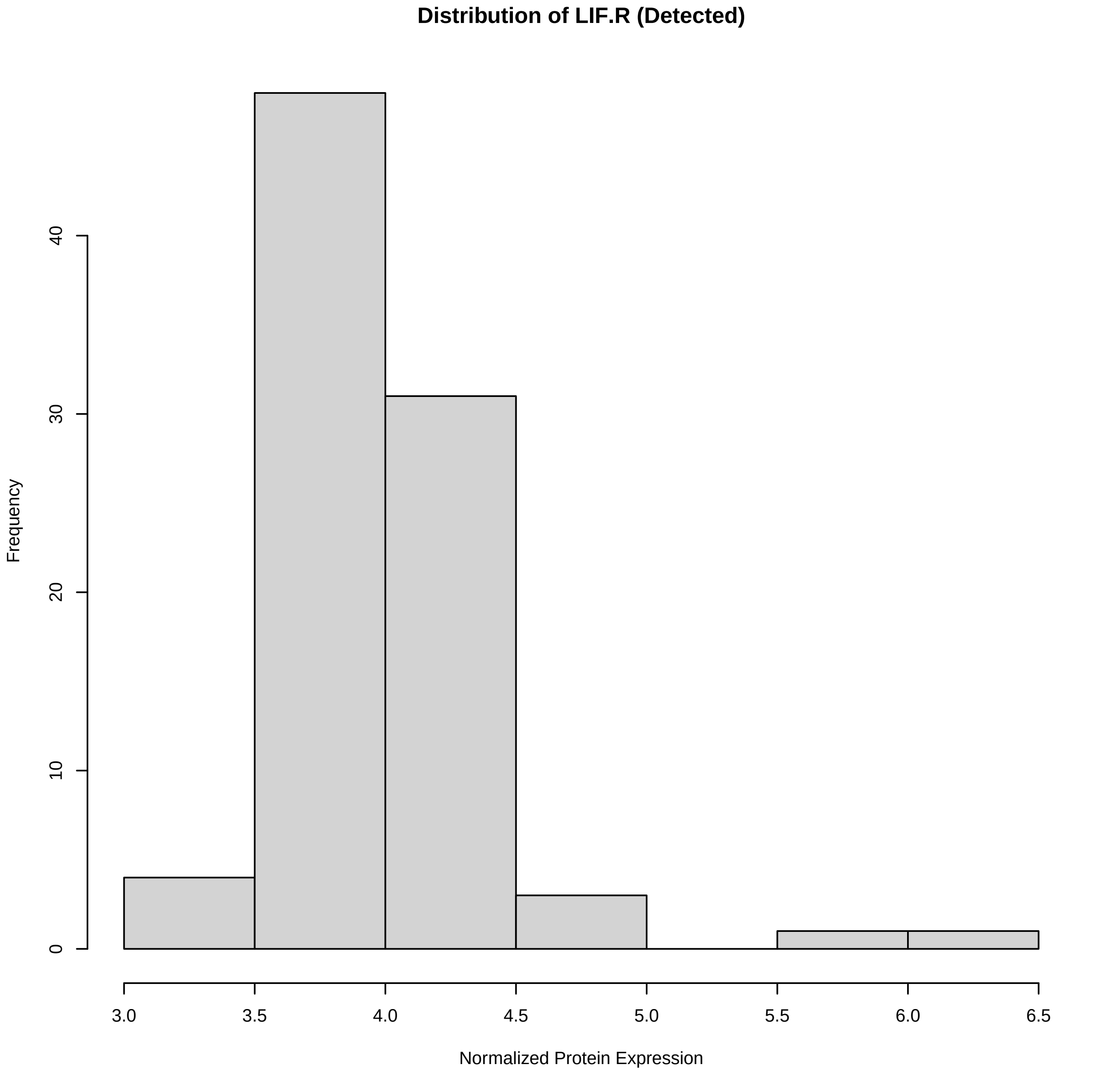
Distribution of LIF.R (Detected)
Frequency
0
10
20
30
40
3.0
3.5
4.0
4.5
5.0
5.5
6.0
6.5
Normalized Protein Expression

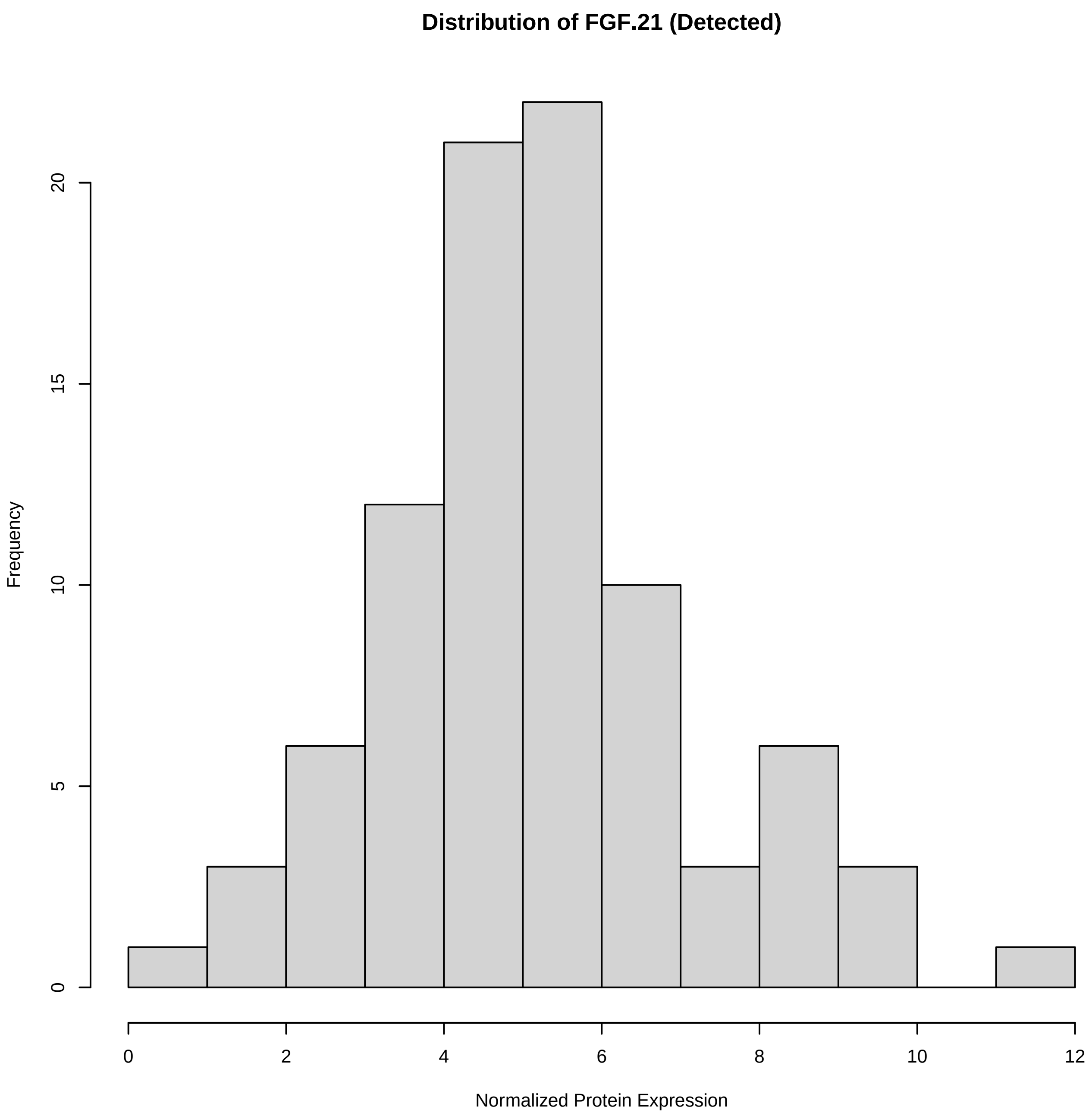

Distribution of FGF.21 (Detected)
Frequency
0
5
10
15
20
0
2
4
6
8
10
12
Normalized Protein Expression

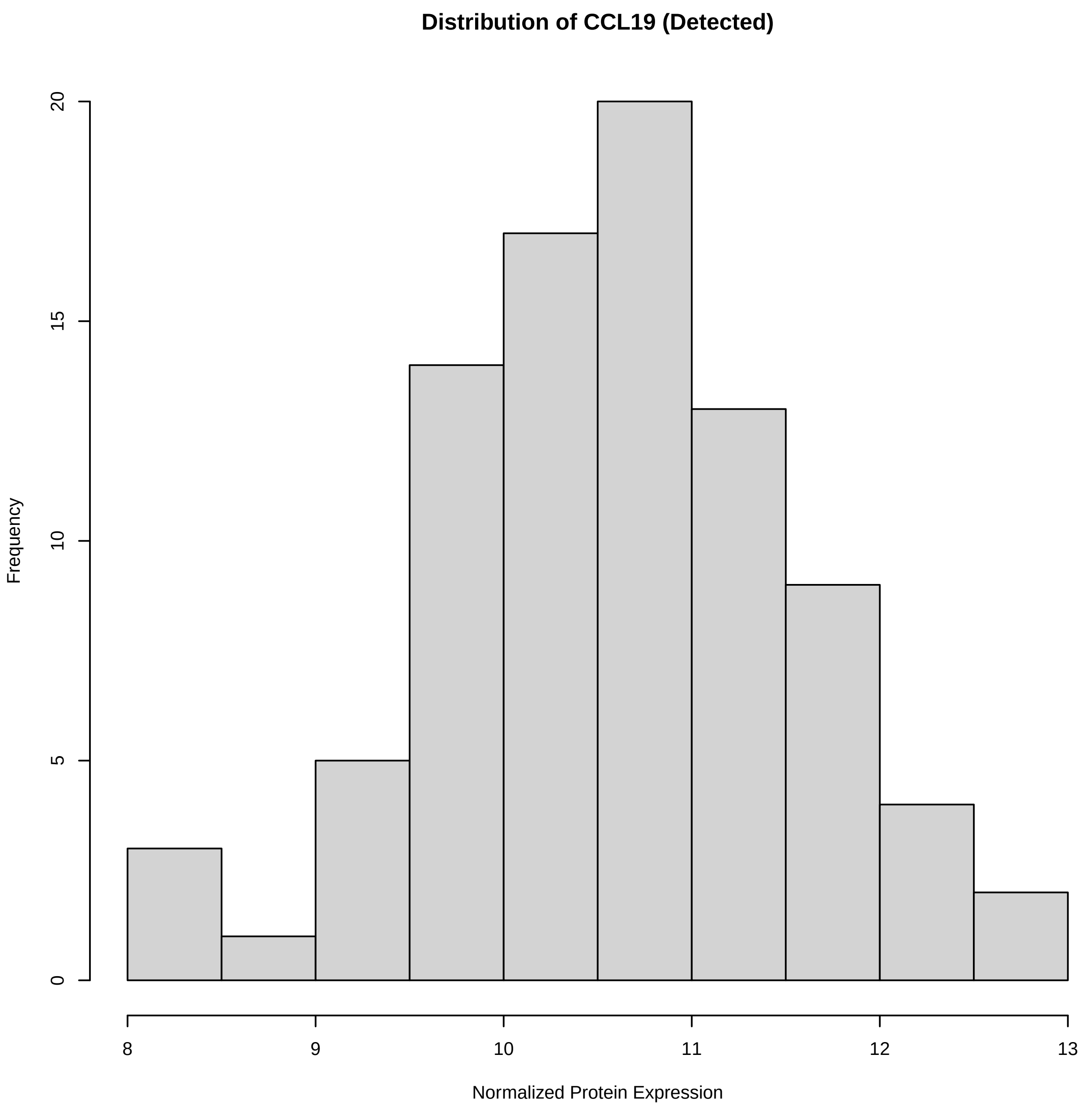

Distribution of CCL19 (Detected)
Frequency
0
5
10
15
20
8
9
10
11
12
13
Normalized Protein Expression

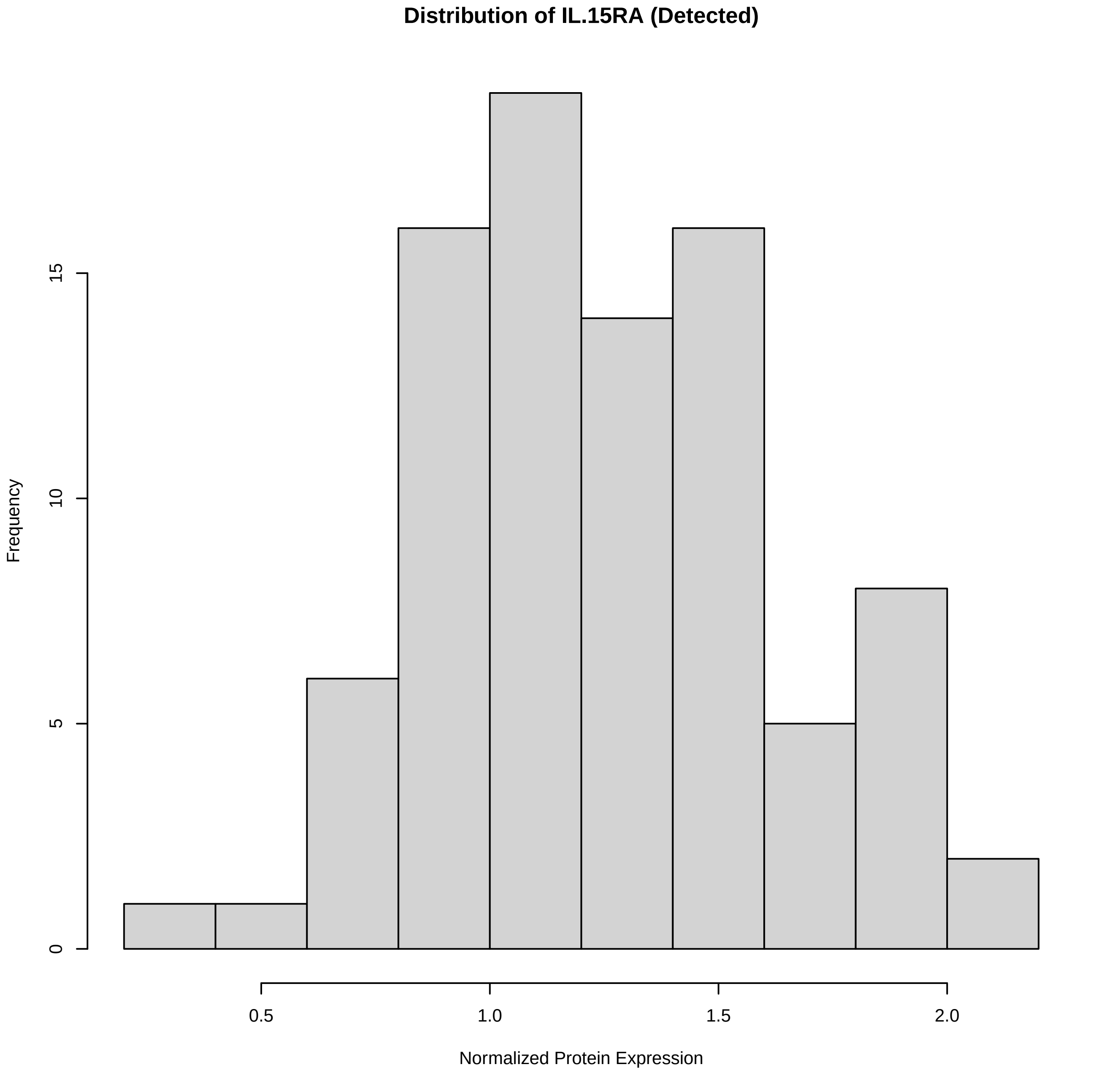

Distribution of IL.15RA (Detected)
Frequency
0
5
10
15
0.5
1.0
1.5
2.0
Normalized Protein Expression

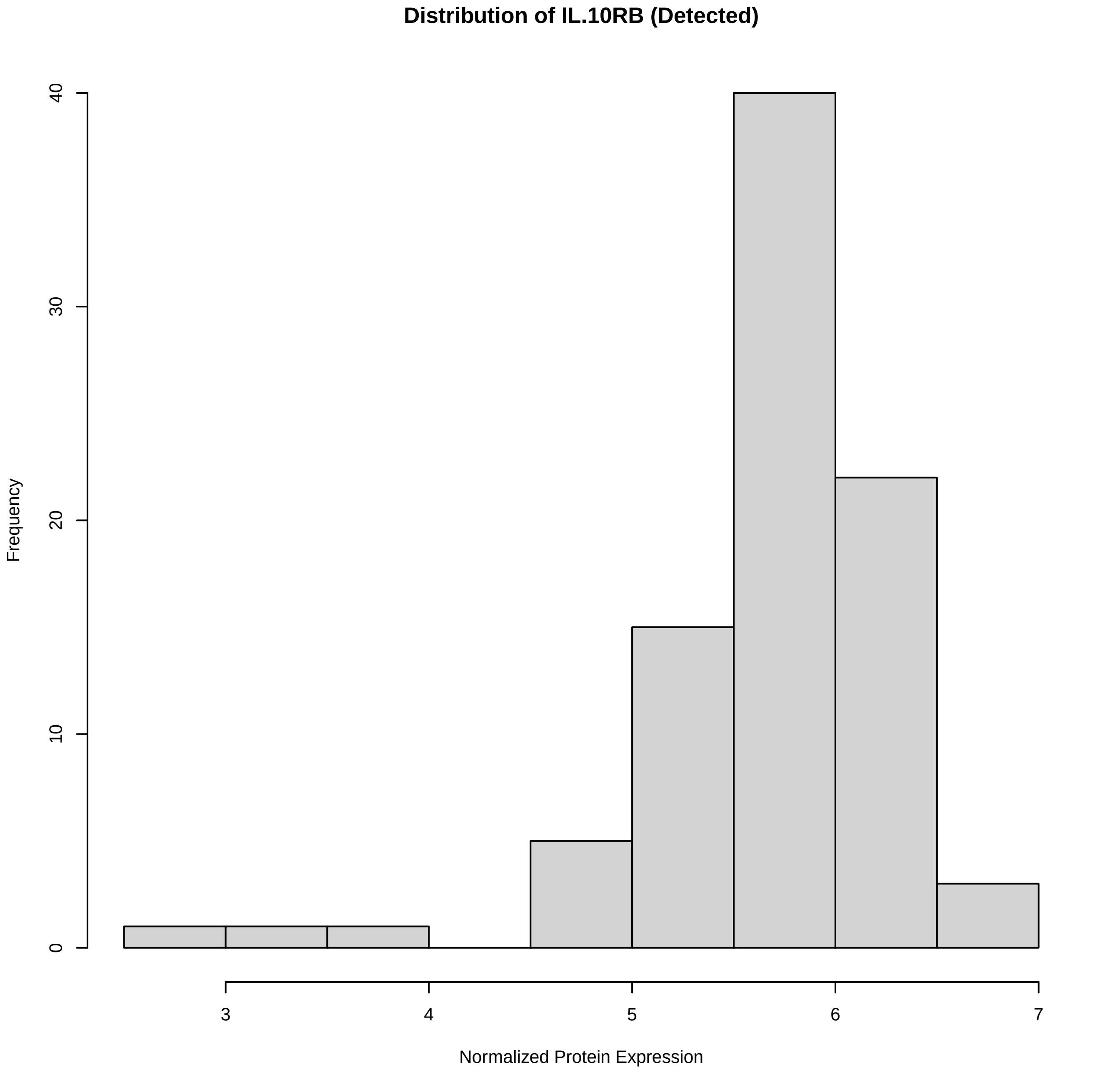

Distribution of IL.10RB (Detected)
Frequency
0
10
20
30
40
3
4
5
6
7
Normalized Protein Expression

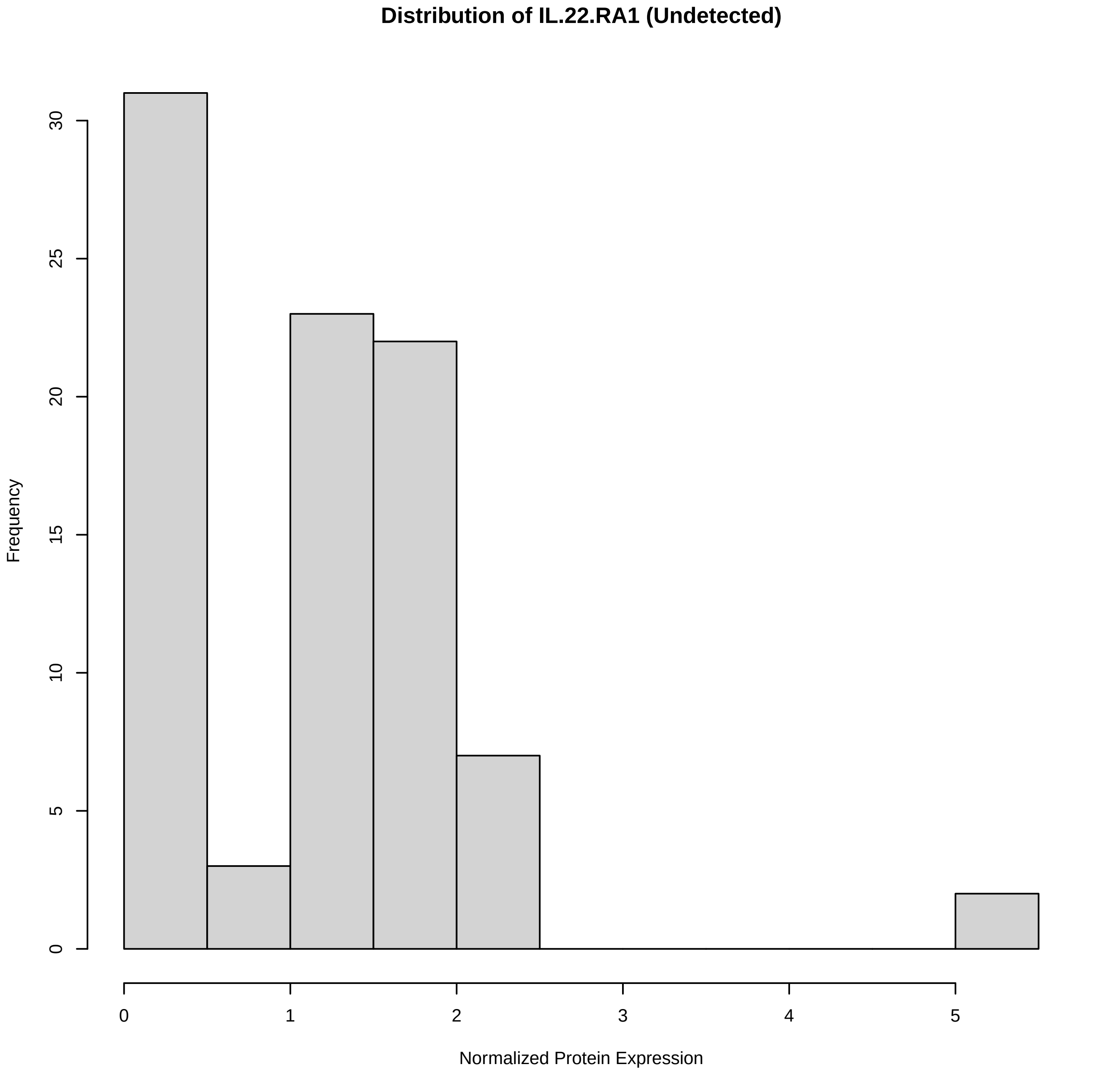

Distribution of IL.22.RA1 (Undetected)
Frequency
0
5
10
15
20
25
30
0
1
2
3
4
5
Normalized Protein Expression

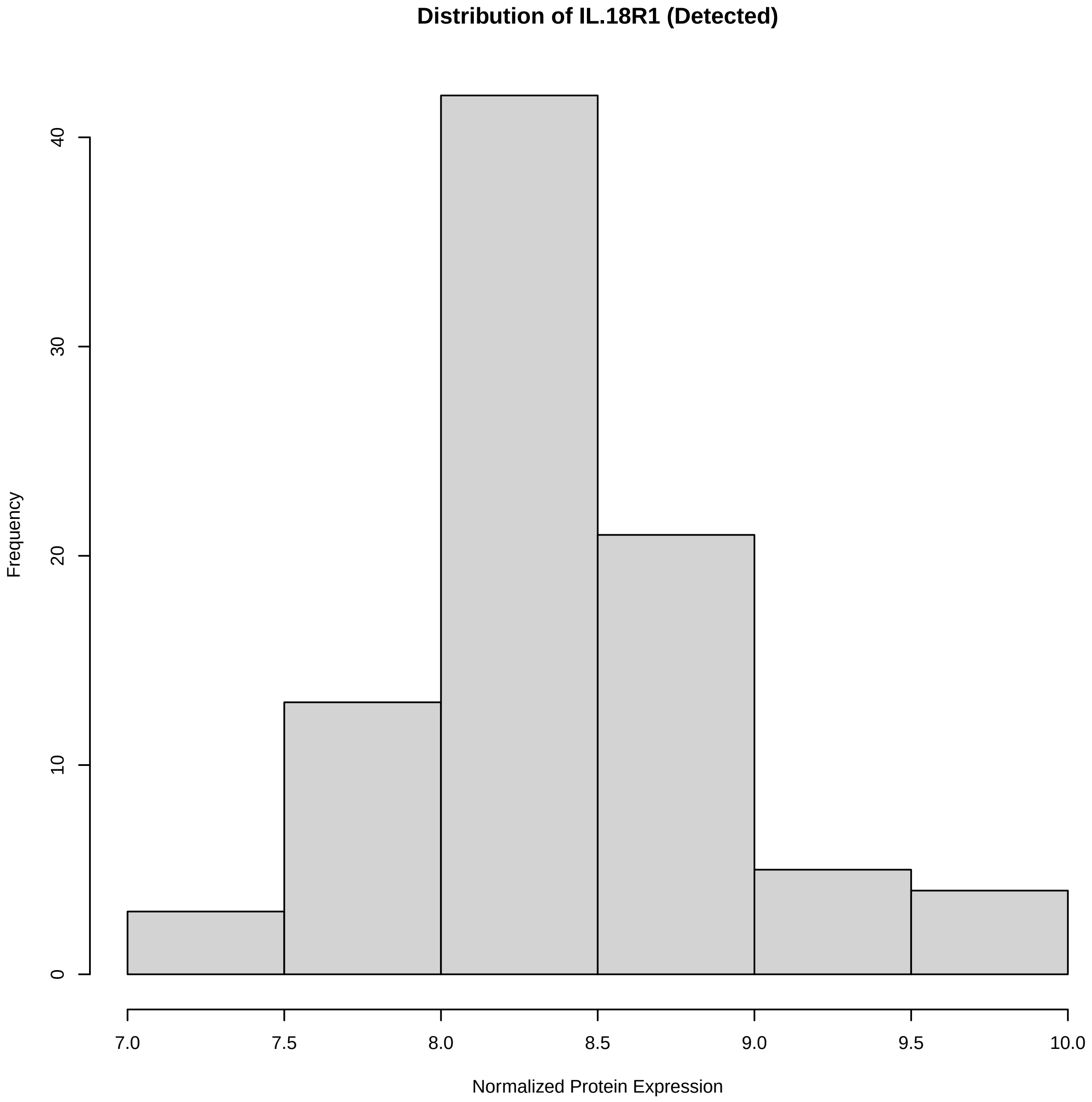

Distribution of IL.18R1 (Detected)
Frequency
0
10
20
30
40
7.0
7.5
8.0
8.5
9.0
9.5
10.0
Normalized Protein Expression

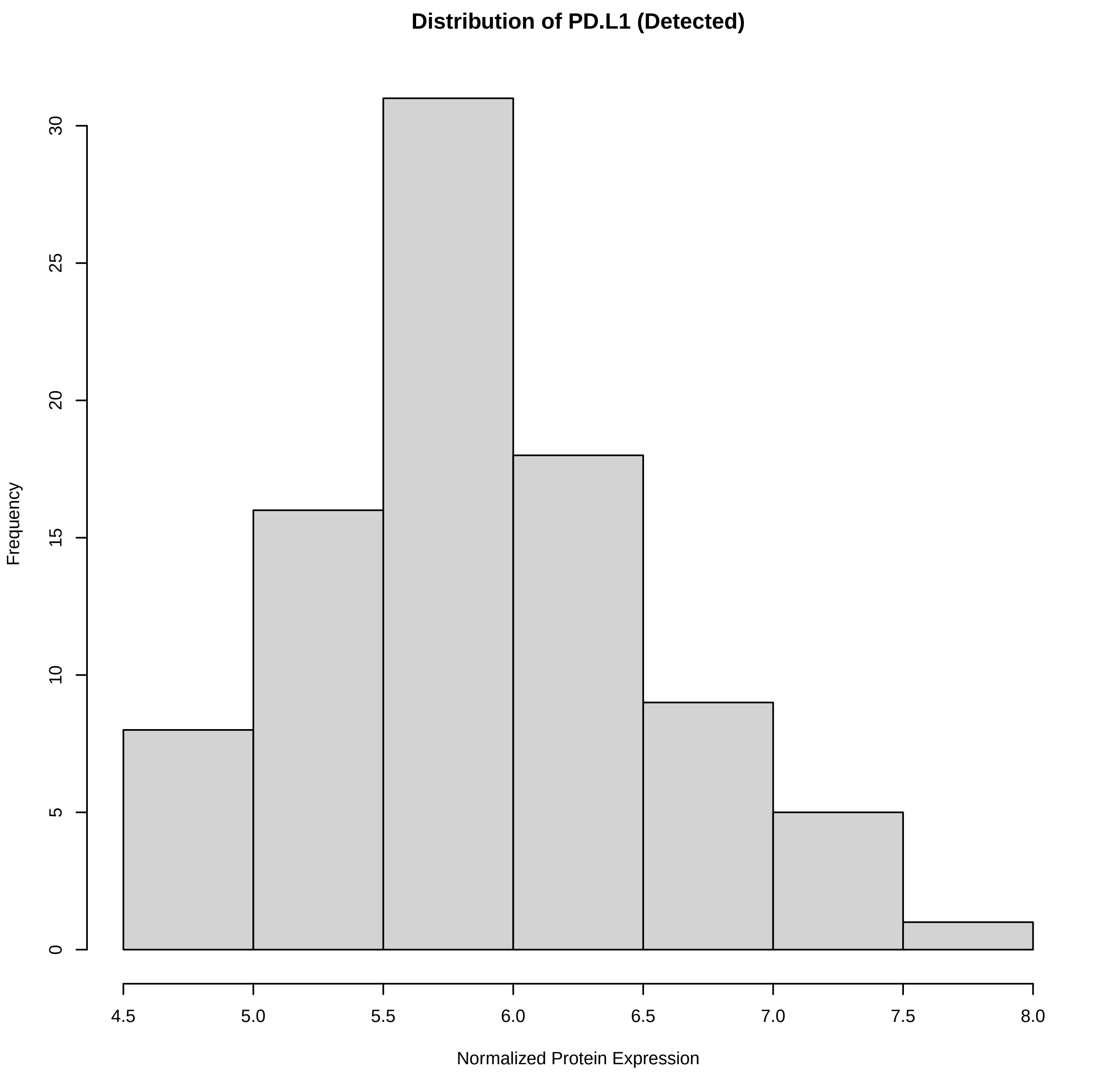

Distribution of PD.L1 (Detected)
Frequency
0
5
10
15
20
25
30
4.5
5.0
5.5
6.0
6.5
7.0
7.5
8.0
Normalized Protein Expression

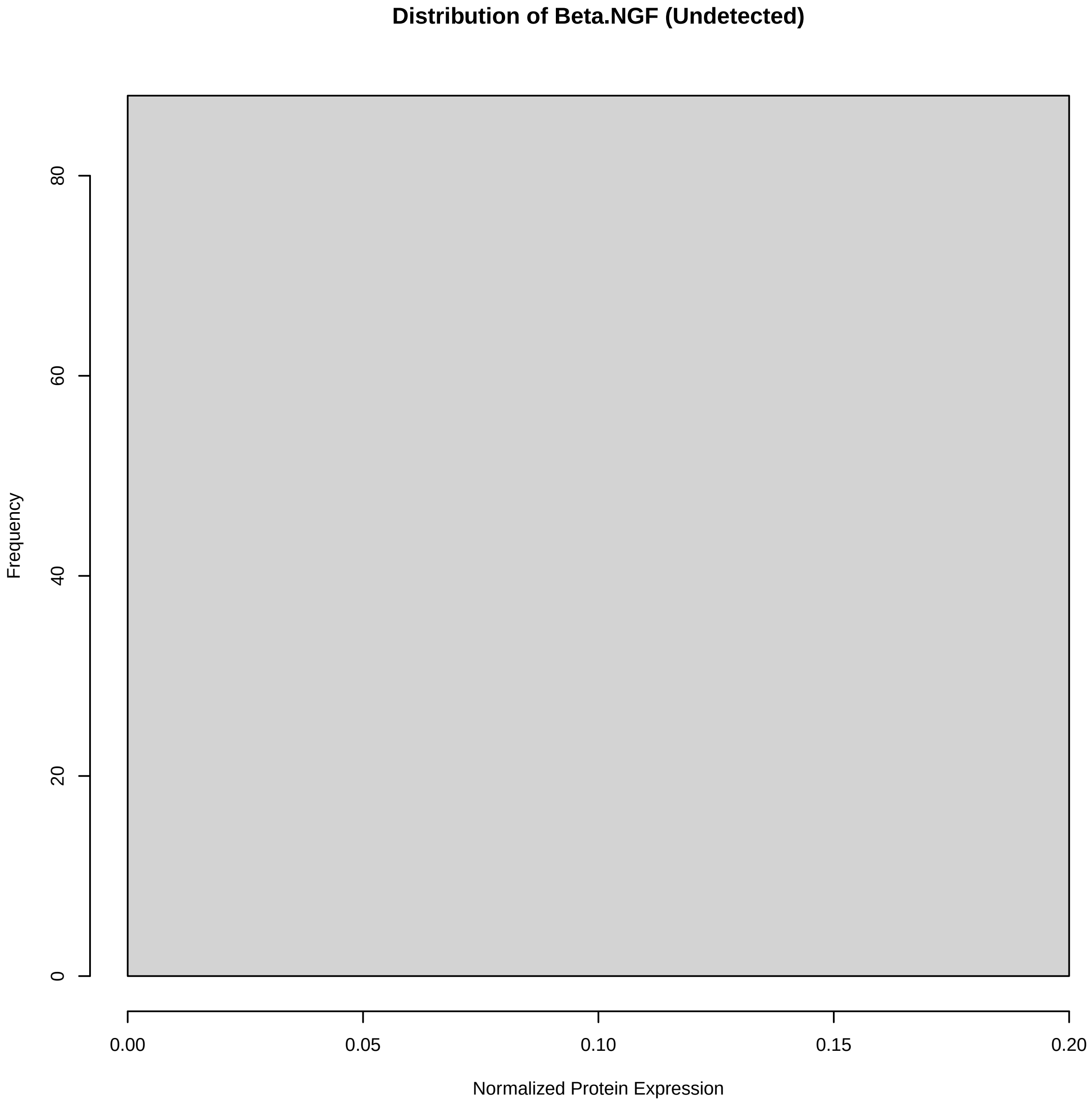

Distribution of Beta.NGF (Undetected)
Frequency
0
20
40
60
80
0.00
0.05
0.10
0.15
0.20
Normalized Protein Expression

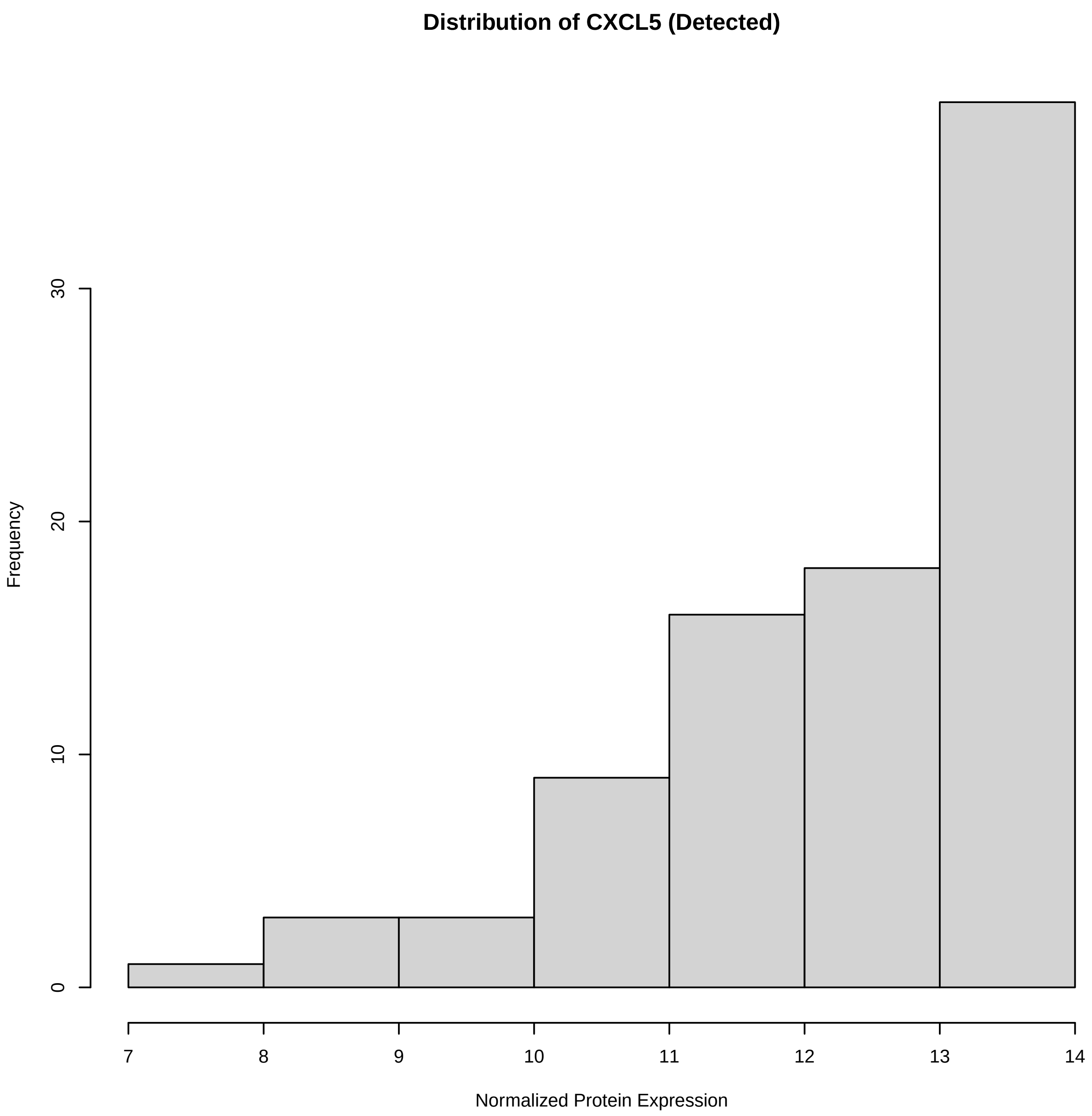
Distribution of CXCL5 (Detected)
Frequency
0
10
20
30
7
8
9
10
11
12
13
14
Normalized Protein Expression

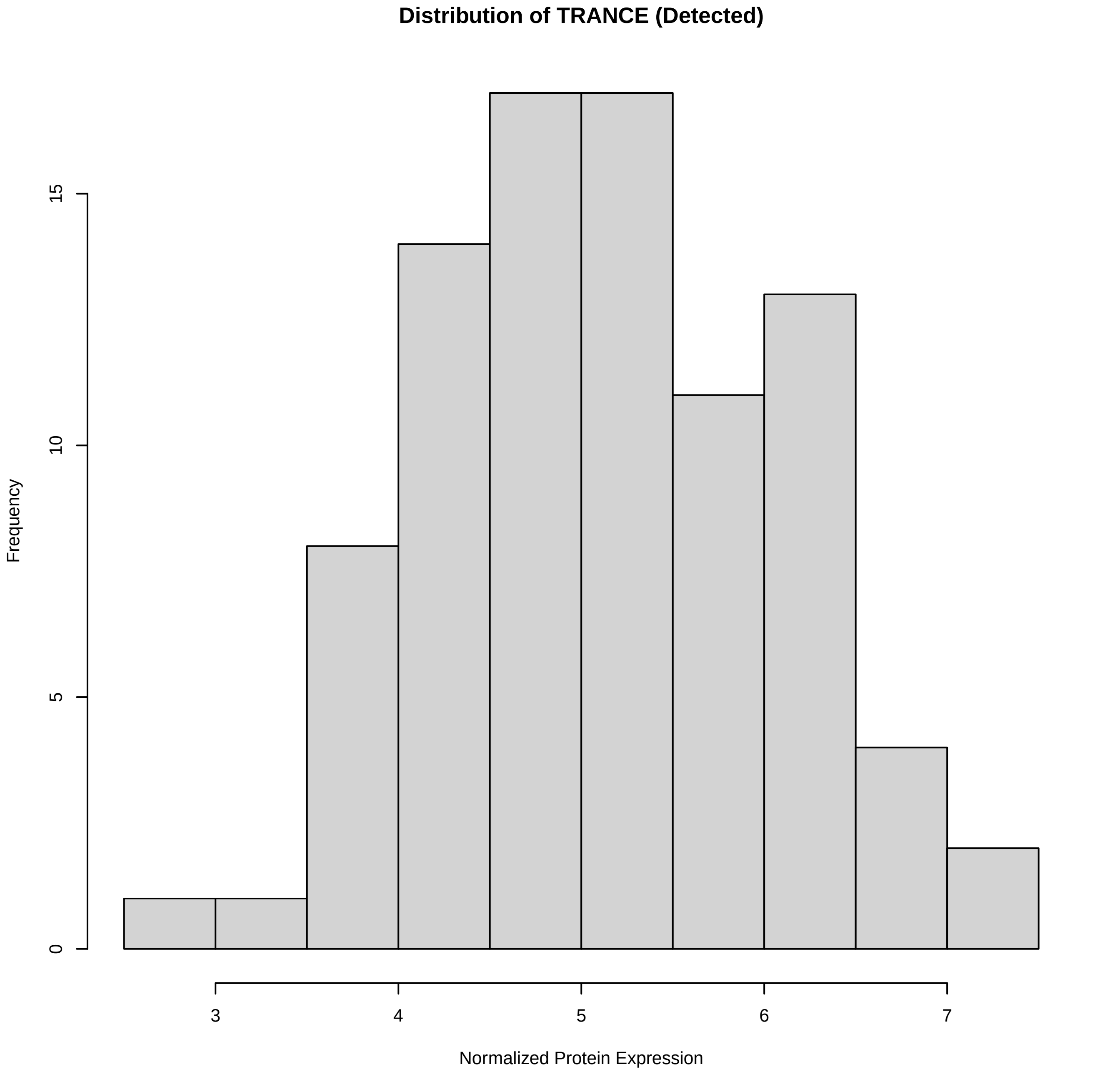
Distribution of TRANCE (Detected)
Frequency
0
5
10
15
3
4
5
6
7
Normalized Protein Expression

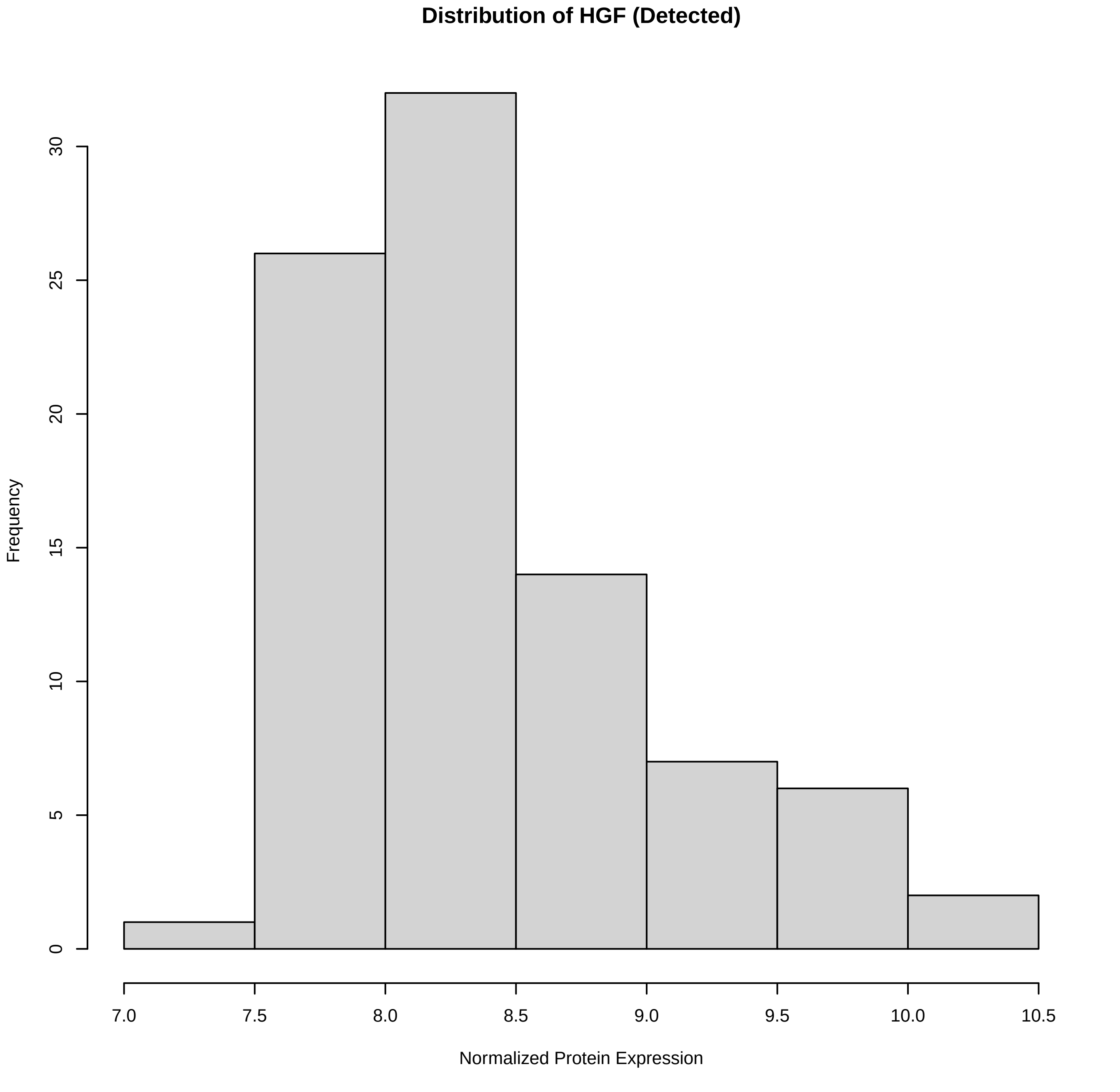

Distribution of HGF (Detected)
Frequency
0
5
10
15
20
25
30
7.0
7.5
8.0
8.5
9.0
9.5
10.0
10.5
Normalized Protein Expression

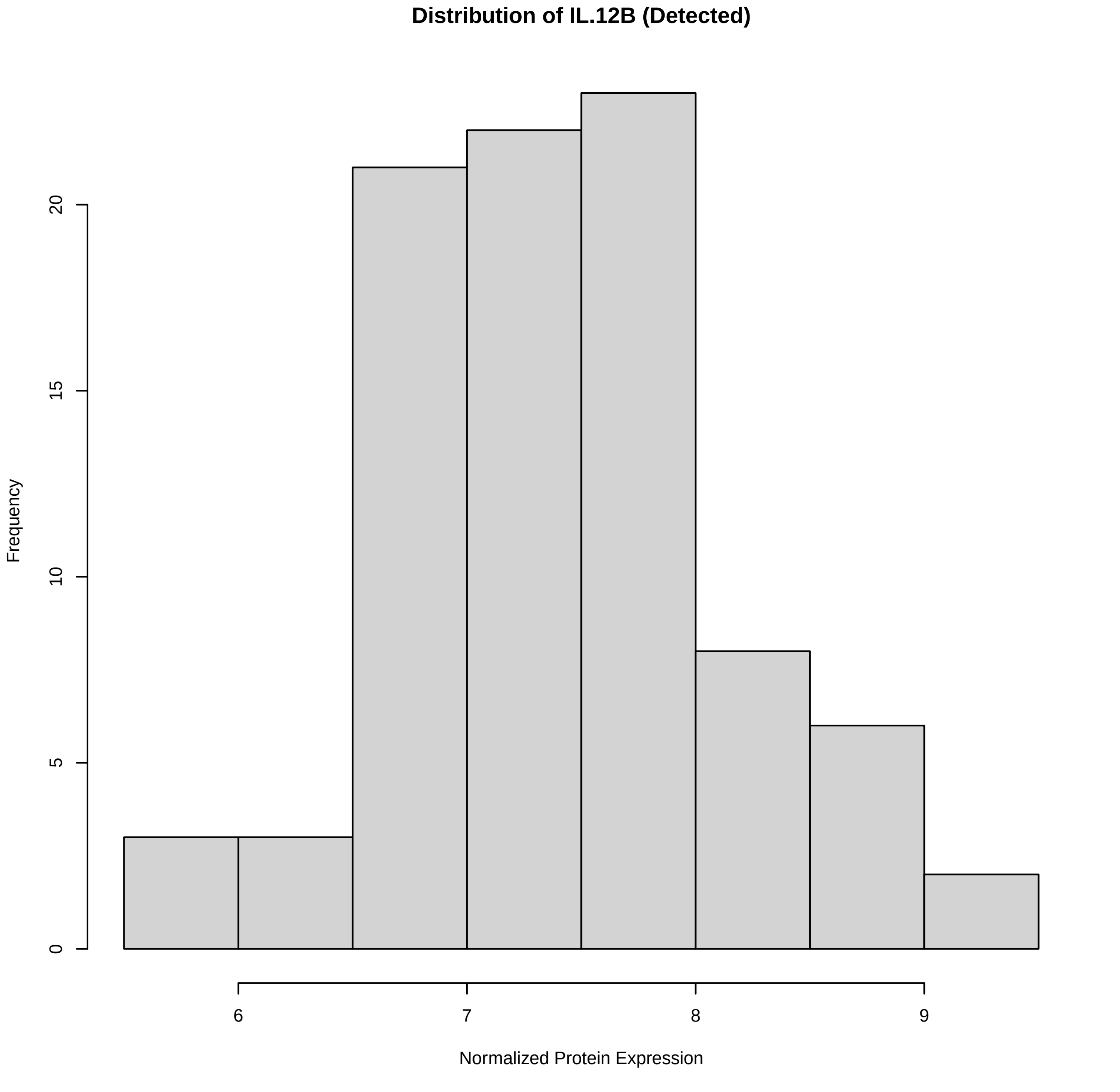
Distribution of IL.12B (Detected)
Frequency
0
5
10
15
20
6
7
8
9
Normalized Protein Expression

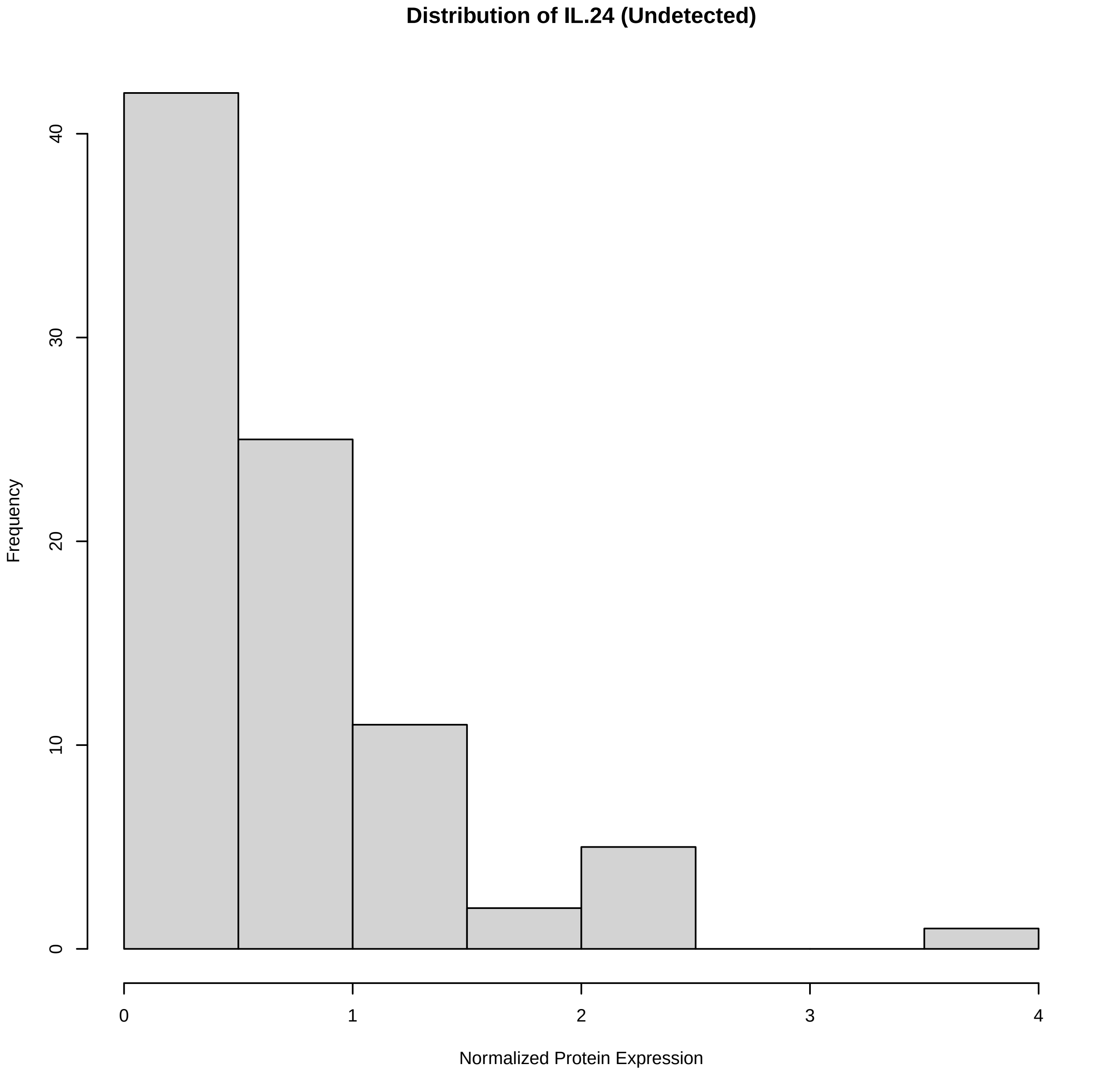

Distribution of IL.24 (Undetected)
Frequency
0
10
20
30
40
0
1
2
3
4
Normalized Protein Expression

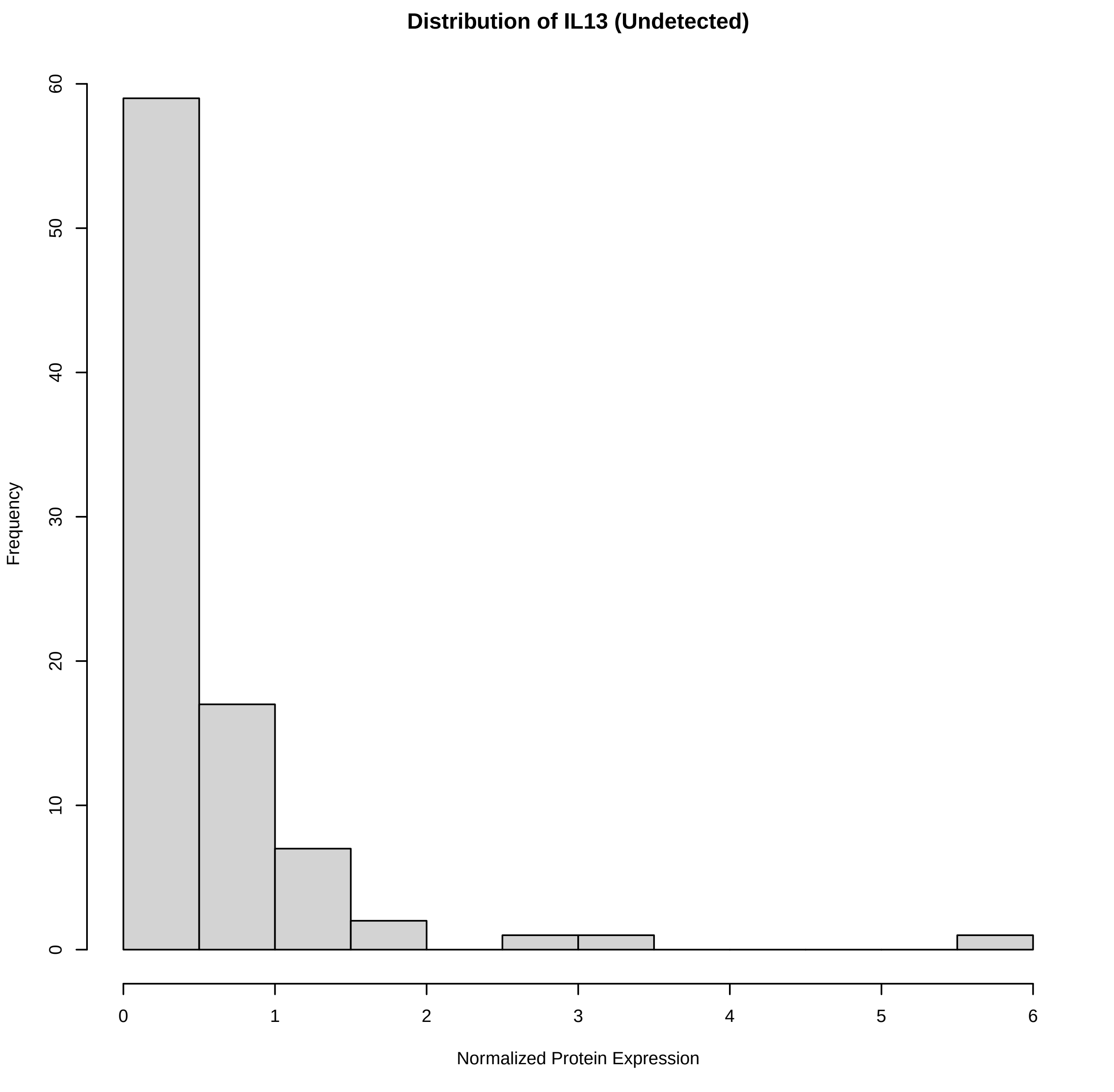

Distribution of IL13 (Undetected)
Frequency
0
10
20
30
40
50
60
0
1
2
3
4
5
6
Normalized Protein Expression

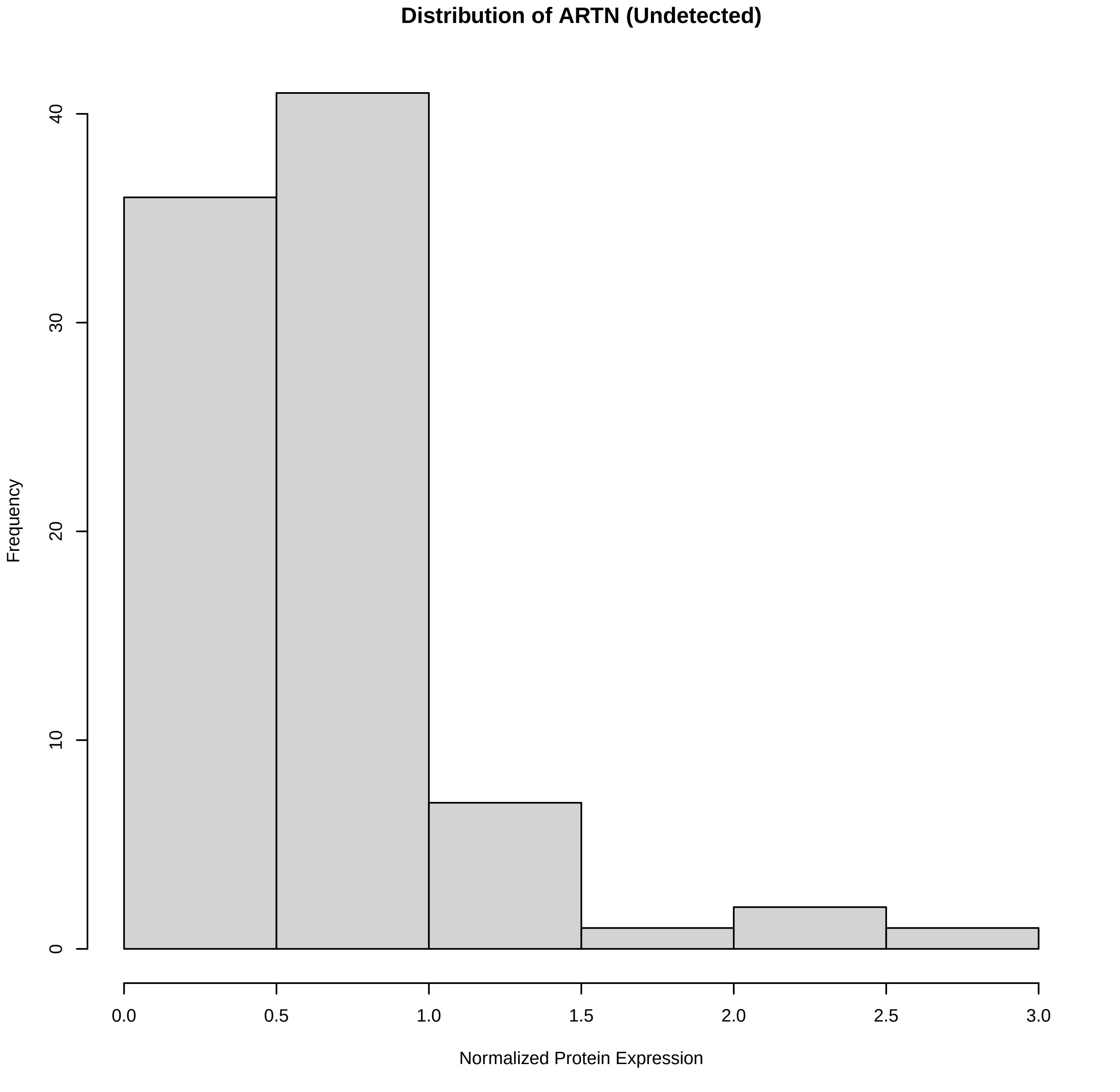
Distribution of ARTN (Undetected)
Frequency
0
10
20
30
40
0.0
0.5
1.0
1.5
2.0
2.5
3.0
Normalized Protein Expression

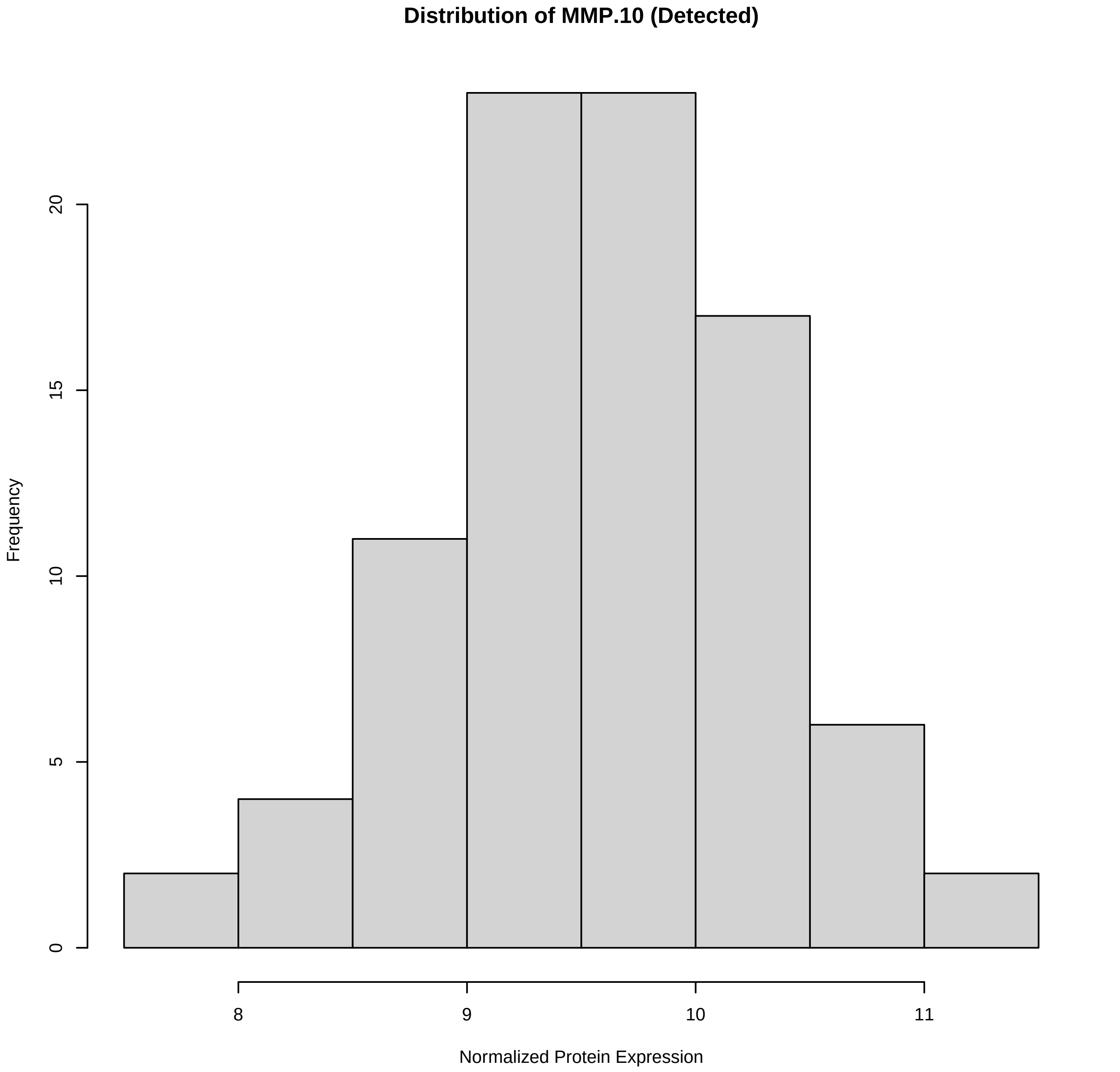
Distribution of MMP.10 (Detected)
Frequency
0
5
10
15
20
8
9
10
11
Normalized Protein Expression

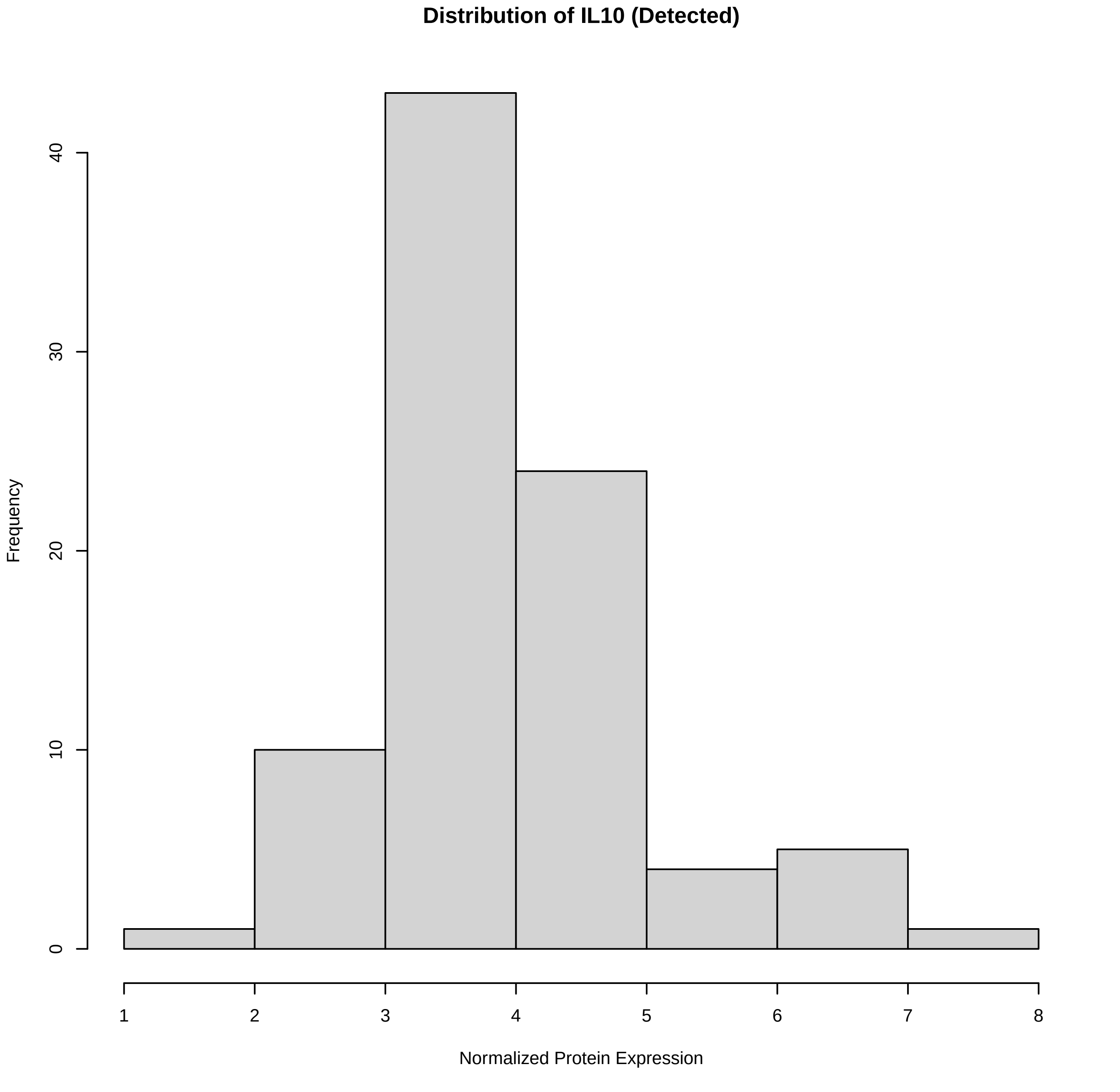

Distribution of IL10 (Detected)
Frequency
0
10
20
30
40
1
2
3
4
5
6
7
8
Normalized Protein Expression

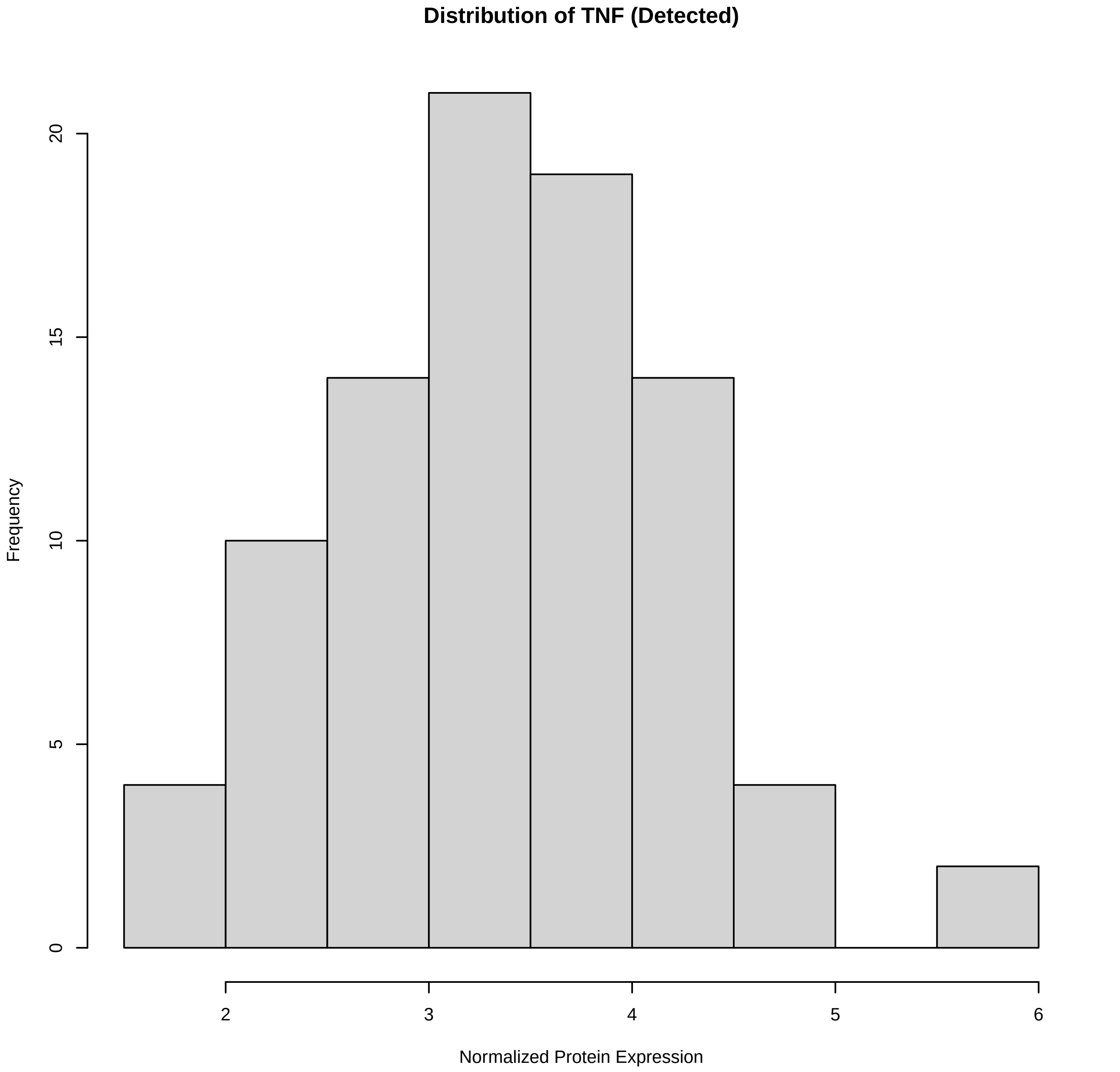

Distribution of TNF (Detected)
Frequency
0
5
10
15
20
2
3
4
5
6
Normalized Protein Expression

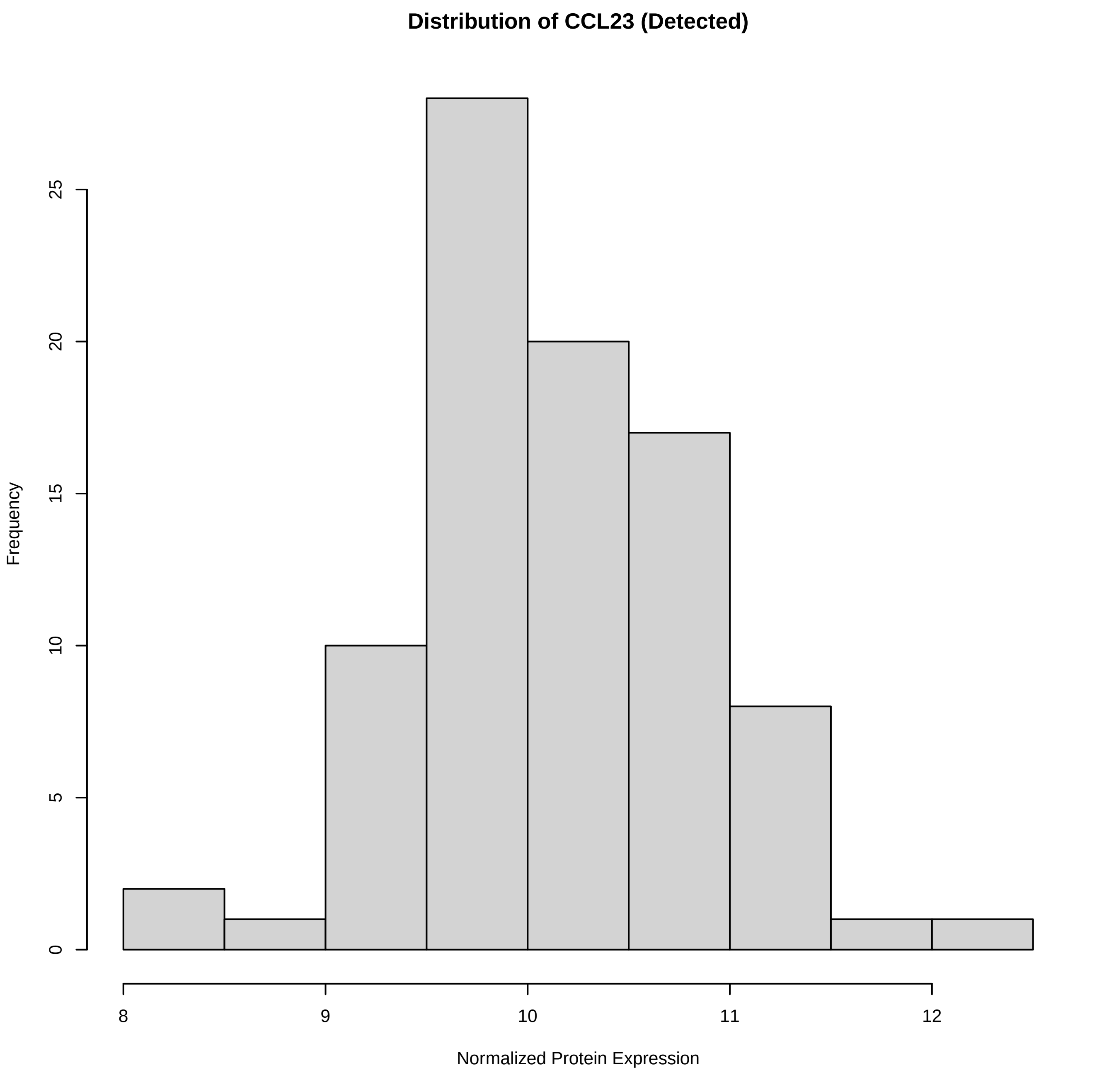
Distribution of CCL23 (Detected)
Frequency
0
5
10
15
20
25
8
9
10
11
12
Normalized Protein Expression

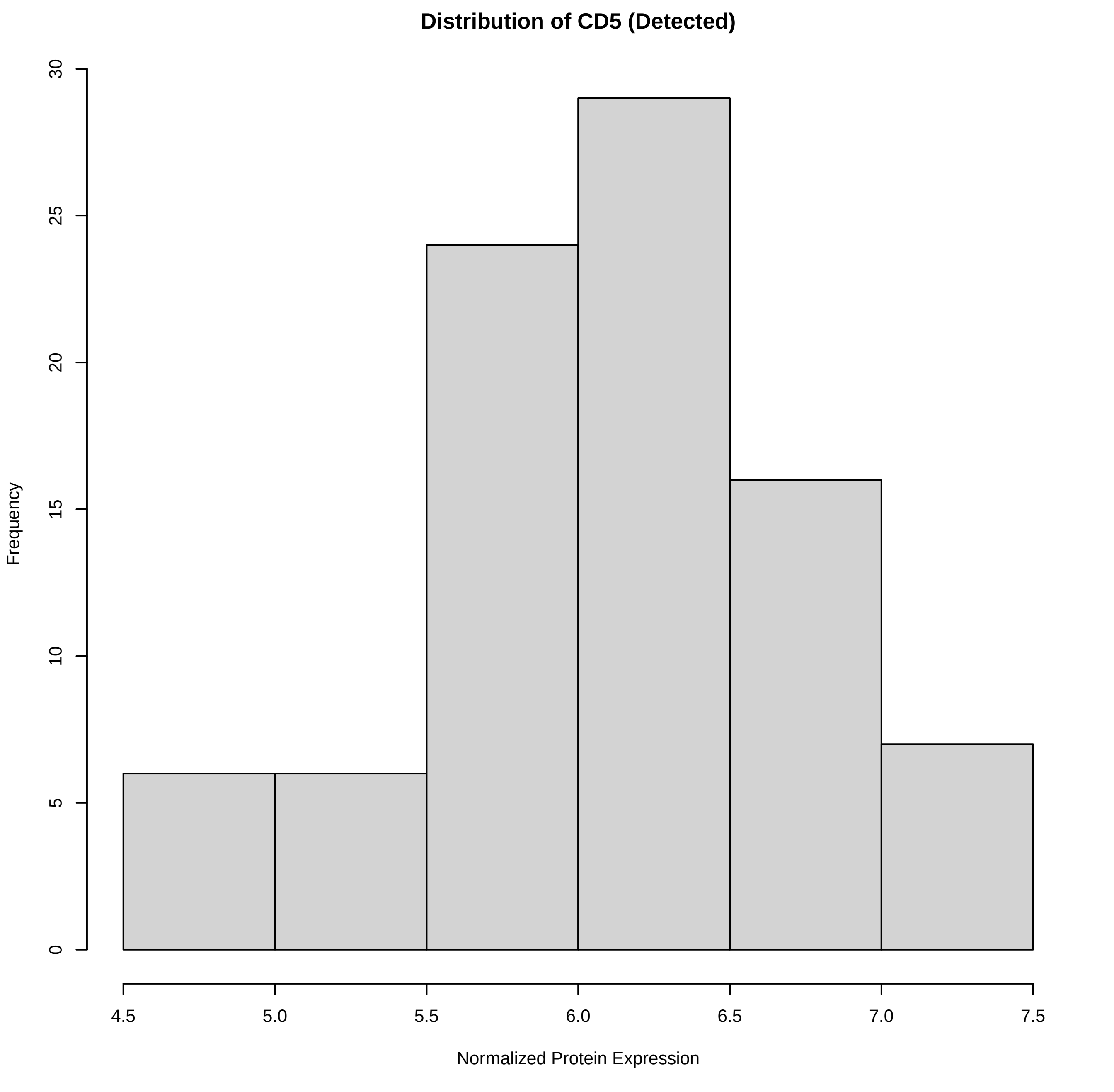
Distribution of CD5 (Detected)
Frequency
0
5
10
15
20
25
30
4.5
5.0
5.5
6.0
6.5
7.0
7.5
Normalized Protein Expression

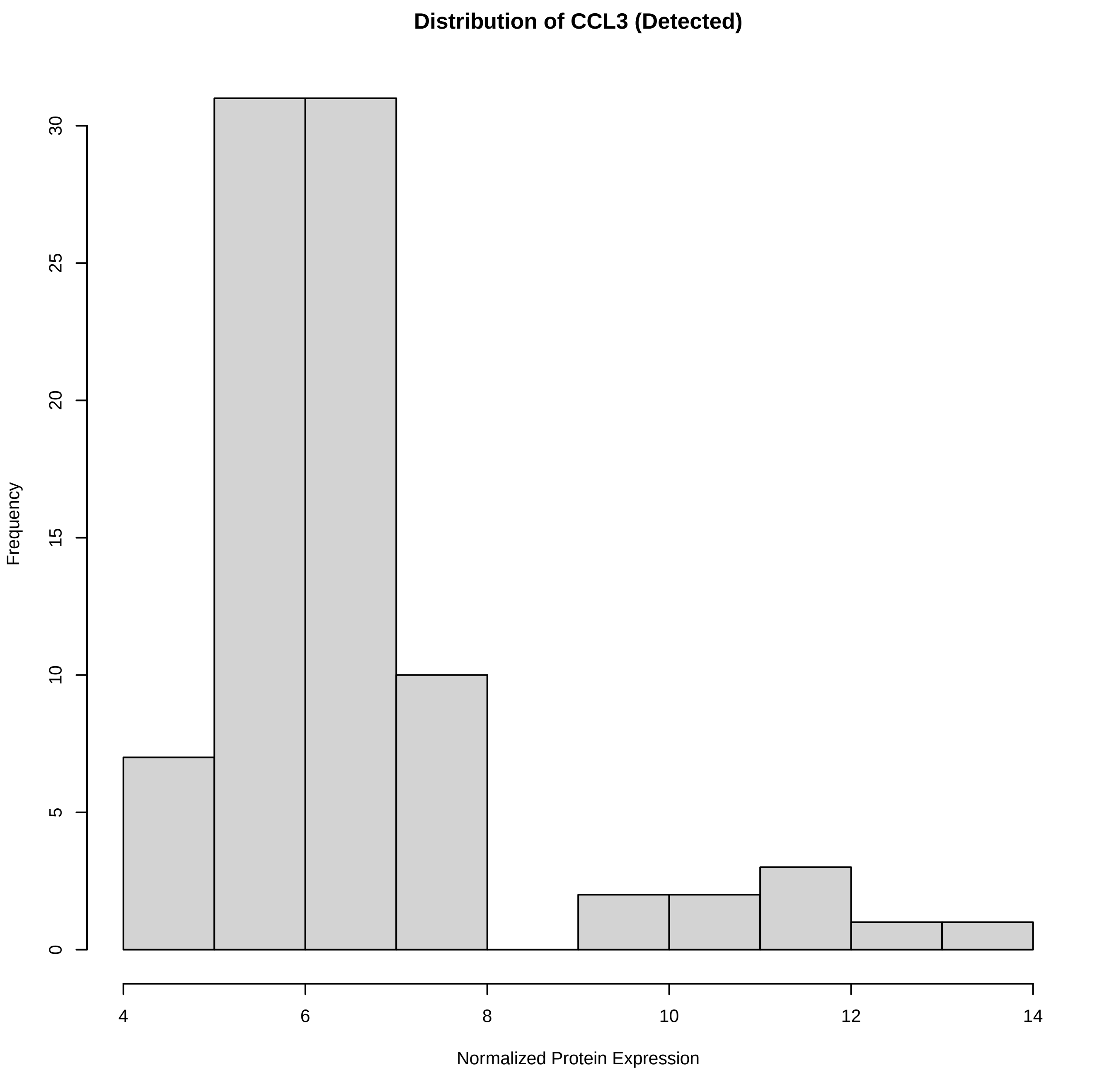

Distribution of CCL3 (Detected)
Frequency
0
5
10
15
20
25
30
4
6
8
10
12
14
Normalized Protein Expression

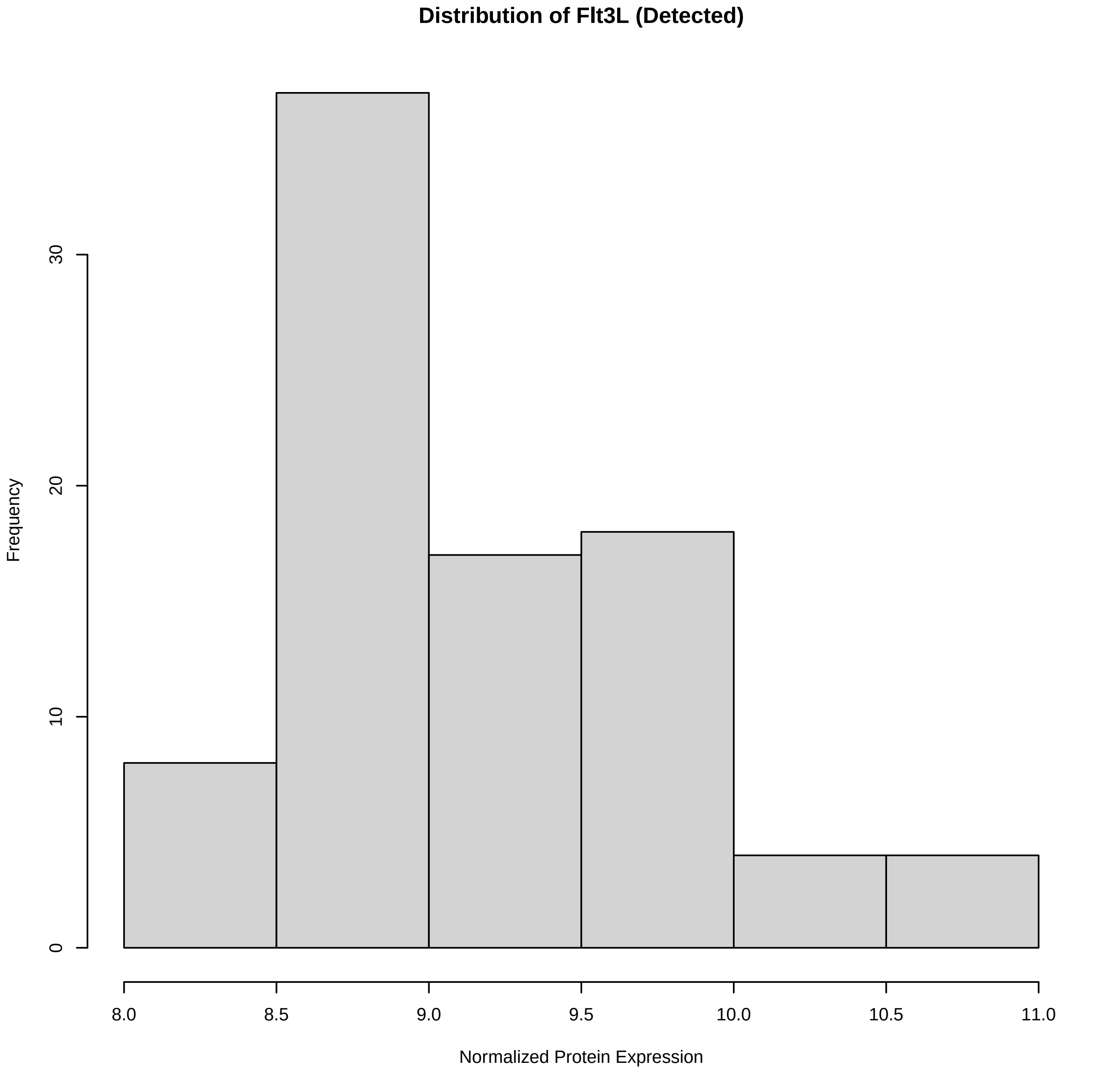
Distribution of Flt3L (Detected)
Frequency
0
10
20
30
8.0
8.5
9.0
9.5
10.0
10.5
11.0
Normalized Protein Expression

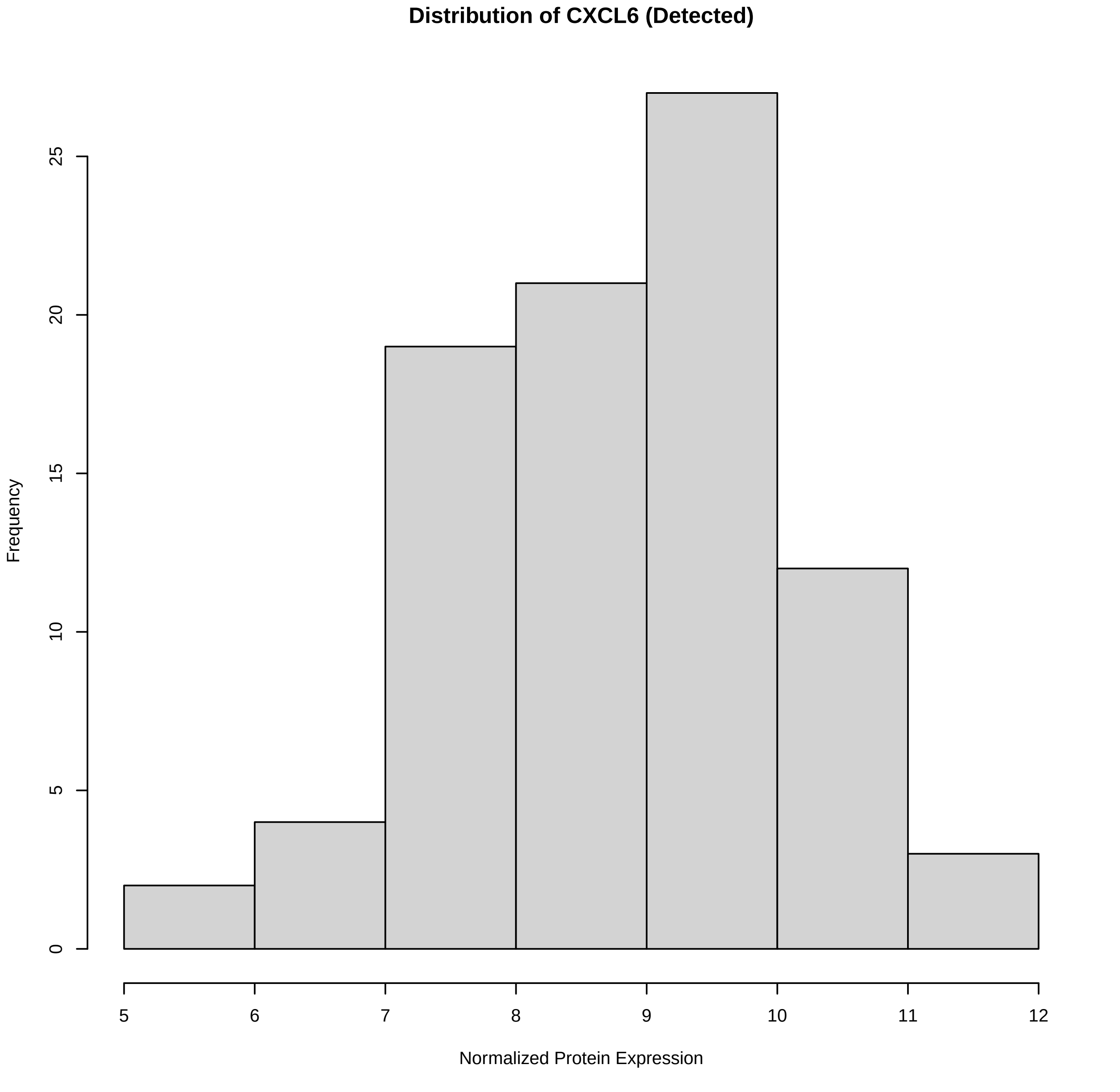

Distribution of CXCL6 (Detected)
Frequency
0
5
10
15
20
25
5
6
7
8
9
10
11
12
Normalized Protein Expression

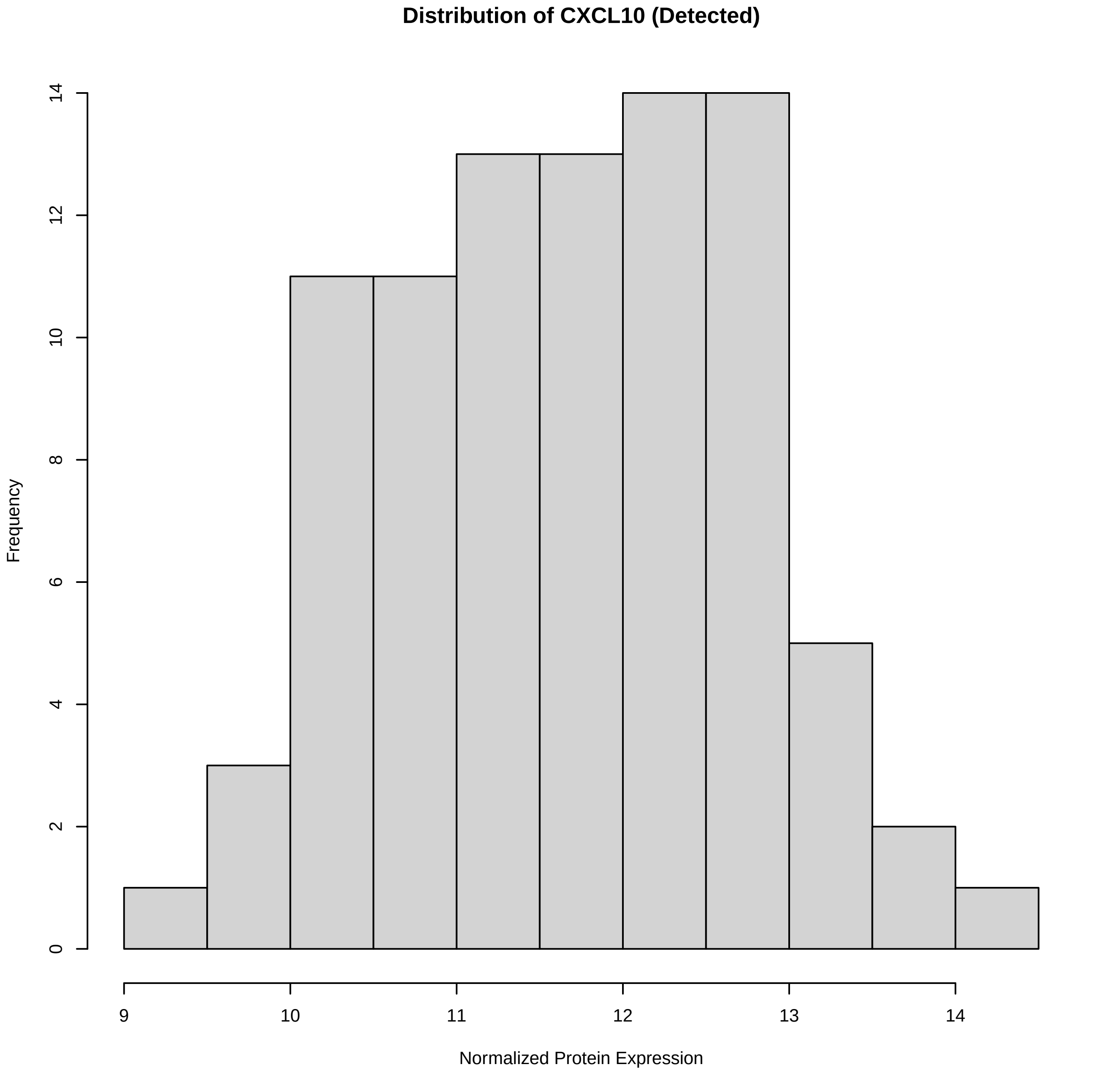
Distribution of CXCL10 (Detected)
Frequency
0
2
4
6
8
10
12
14
9
10
11
12
13
14
Normalized Protein Expression

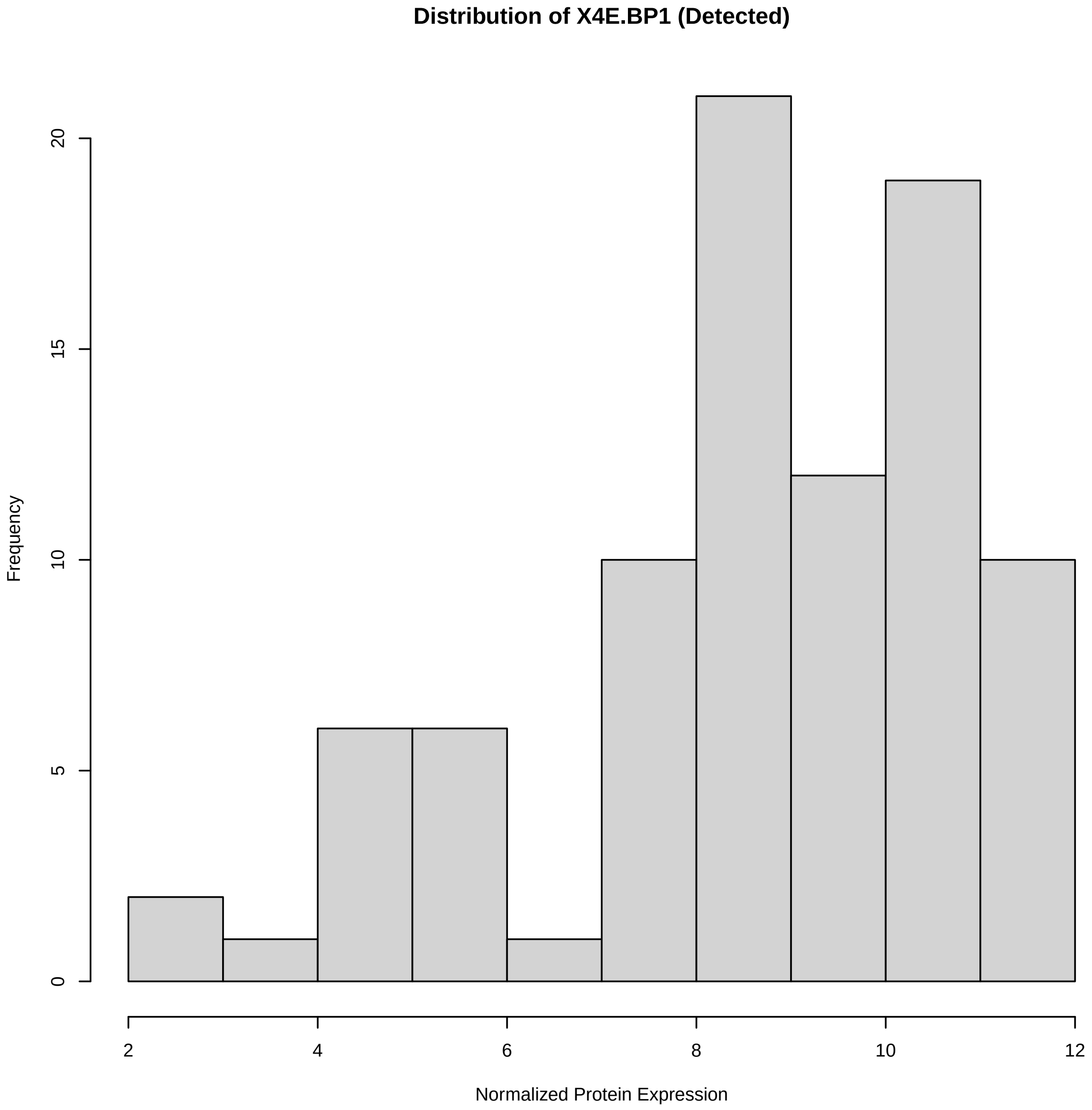
Distribution of X4E.BP1 (Detected)
Frequency
0
5
10
15
20
2
4
6
8
10
12
Normalized Protein Expression

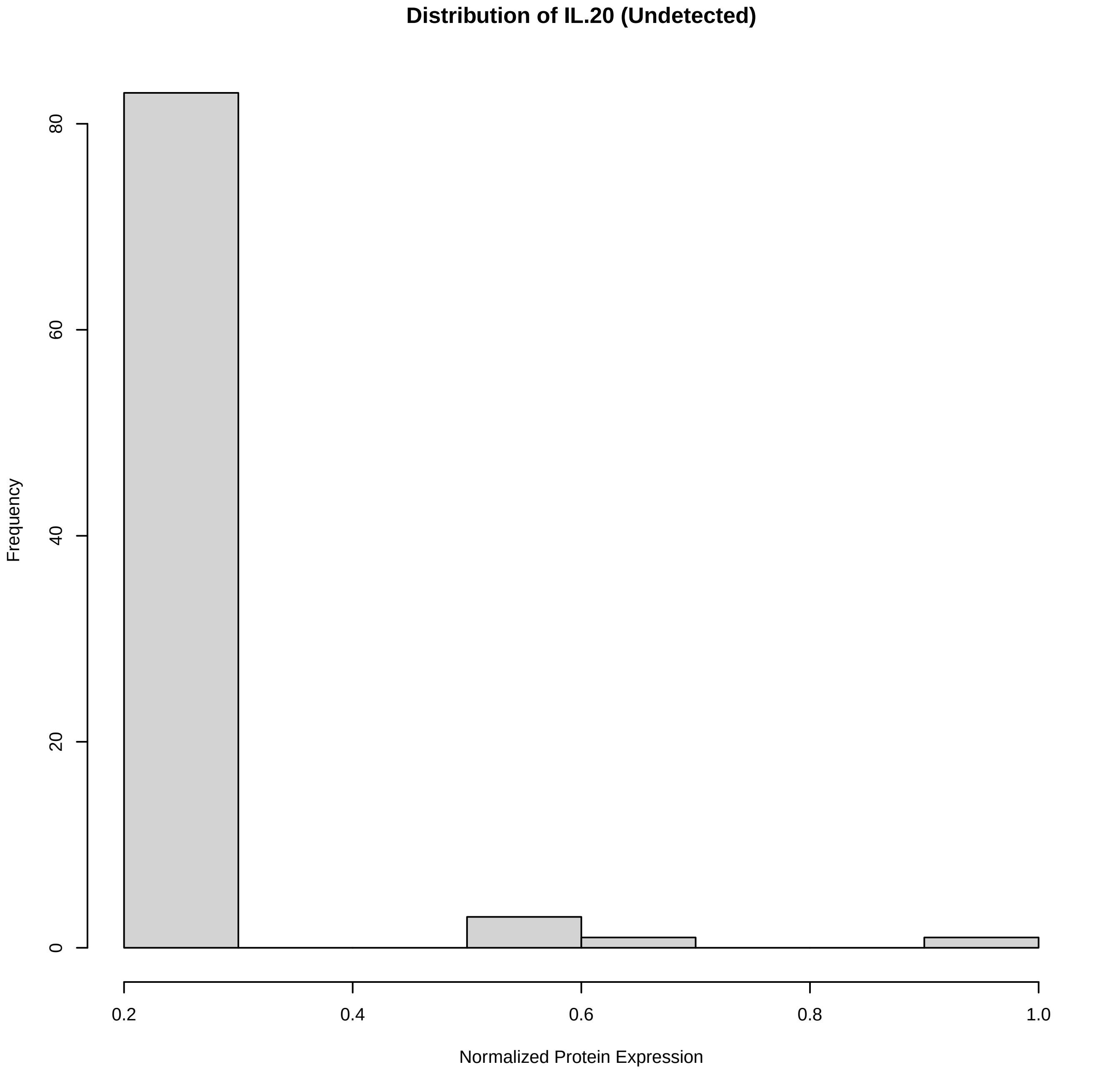
Distribution of IL.20 (Undetected)
Frequency
0
20
40
60
80
0.2
0.4
0.6
0.8
1.0
Normalized Protein Expression

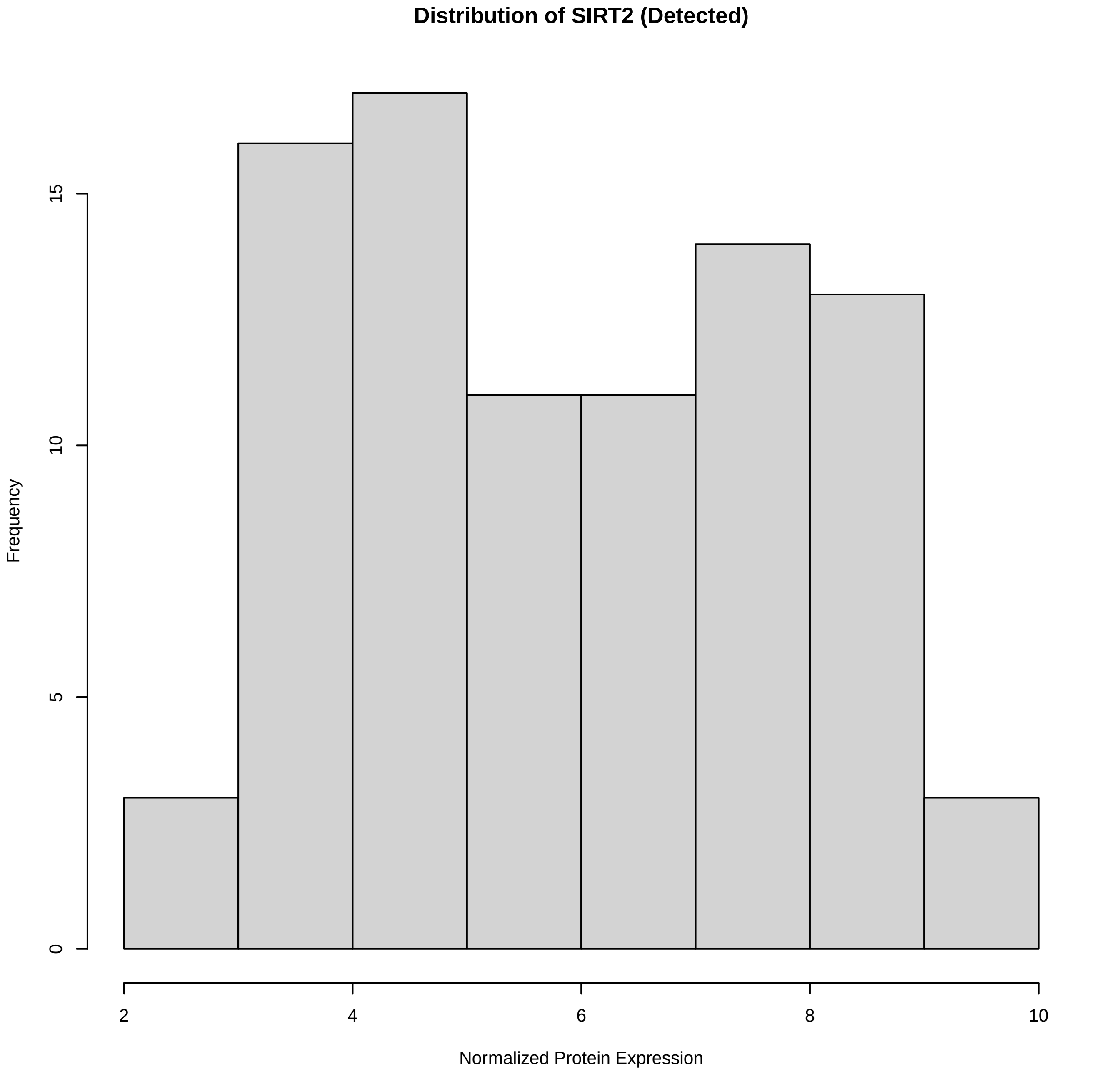

Distribution of SIRT2 (Detected)
Frequency
0
5
10
15
2
4
6
8
10
Normalized Protein Expression

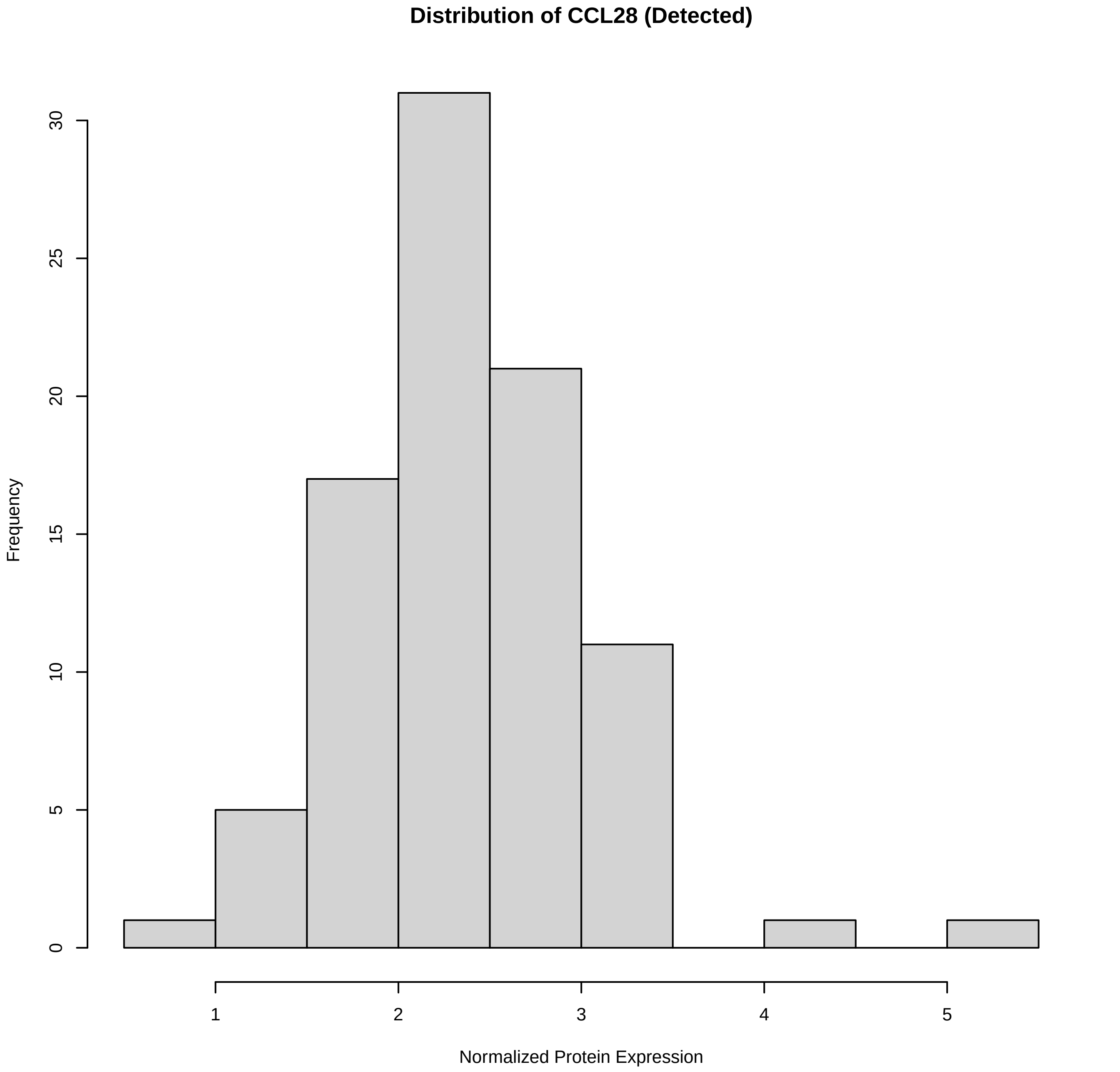
Distribution of CCL28 (Detected)
Frequency
0
5
10
15
20
25
30
1
2
3
4
5
Normalized Protein Expression

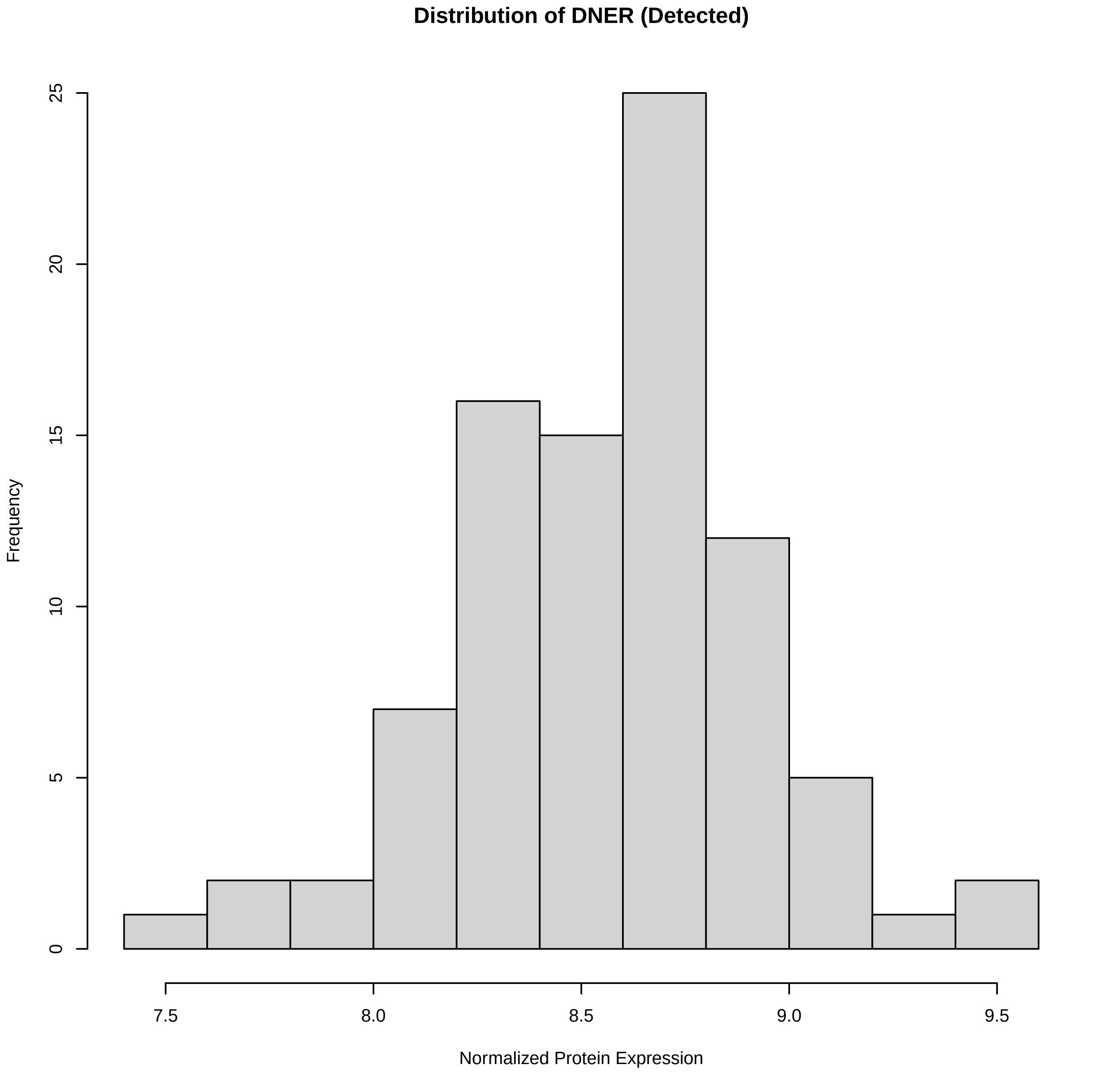
Distribution of DNER (Detected)
Frequency
0
5
10
15
20
25
7.5
8.0
8.5
9.0
9.5
Normalized Protein Expression

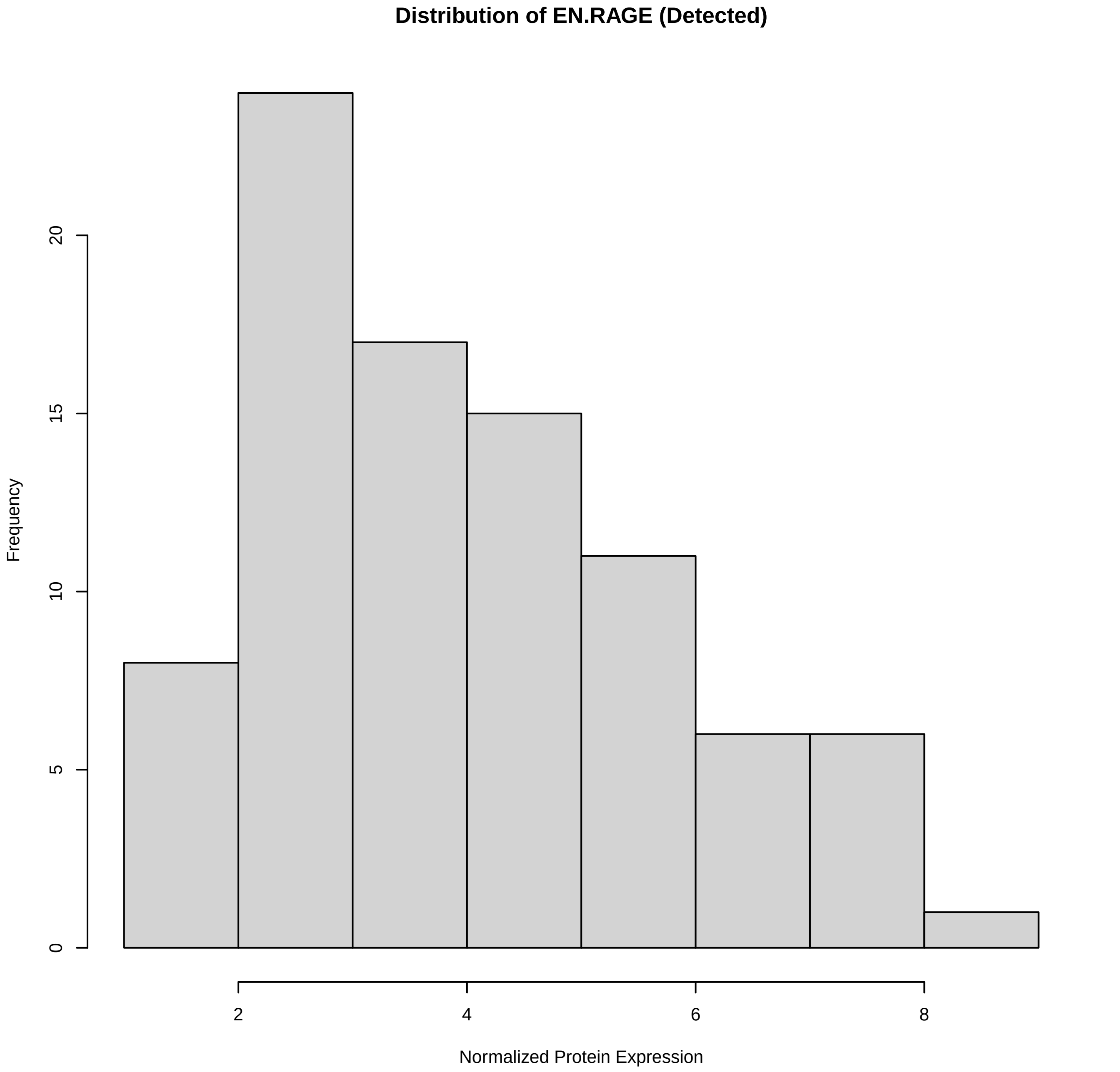

Distribution of EN.RAGE (Detected)
Frequency
0
5
10
15
20
2
4
6
8
Normalized Protein Expression

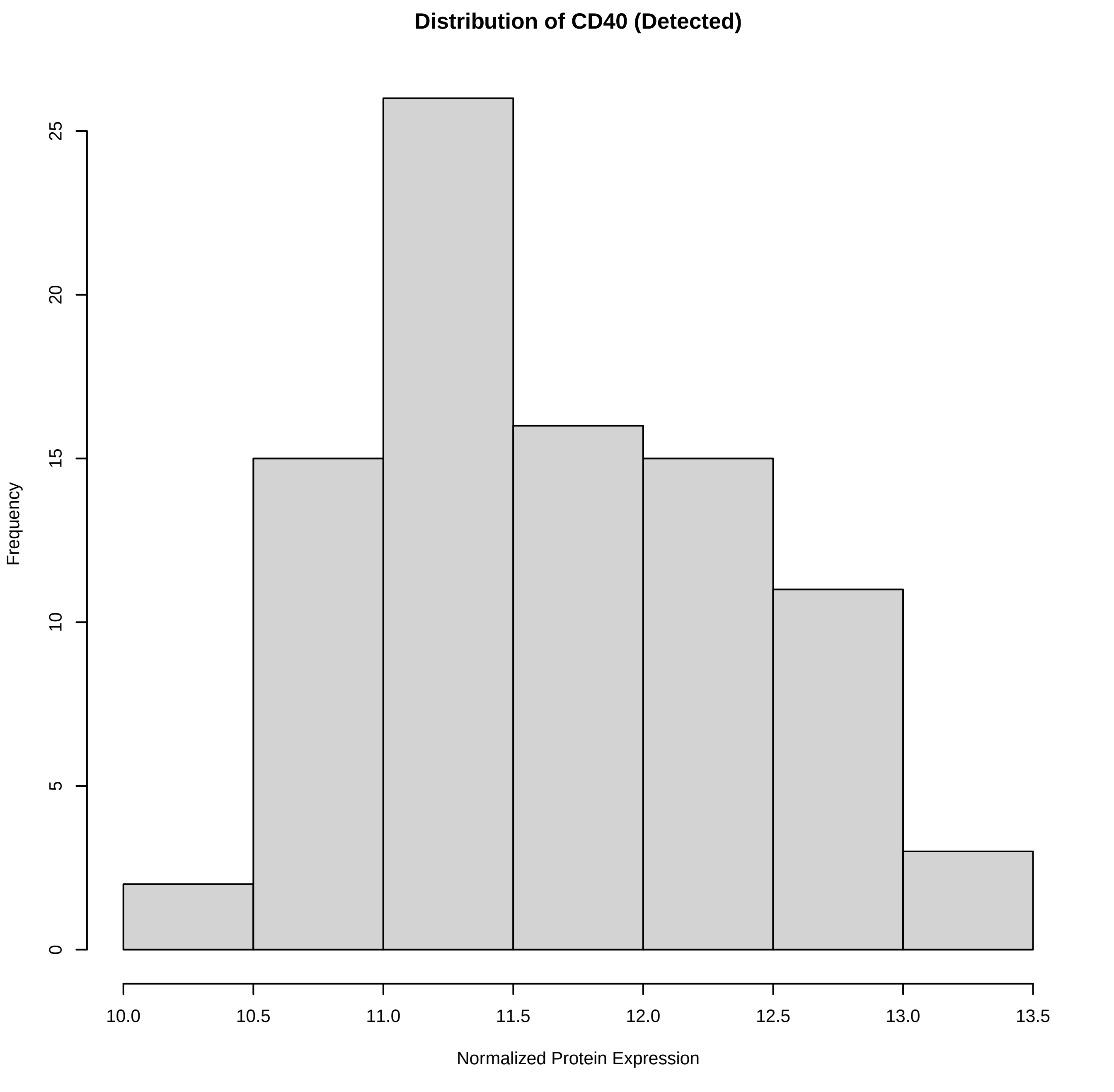
Distribution of CD40 (Detected)
Frequency
0
5
10
15
20
25
10.0
10.5
11.0
11.5
12.0
12.5
13.0
13.5
Normalized Protein Expression

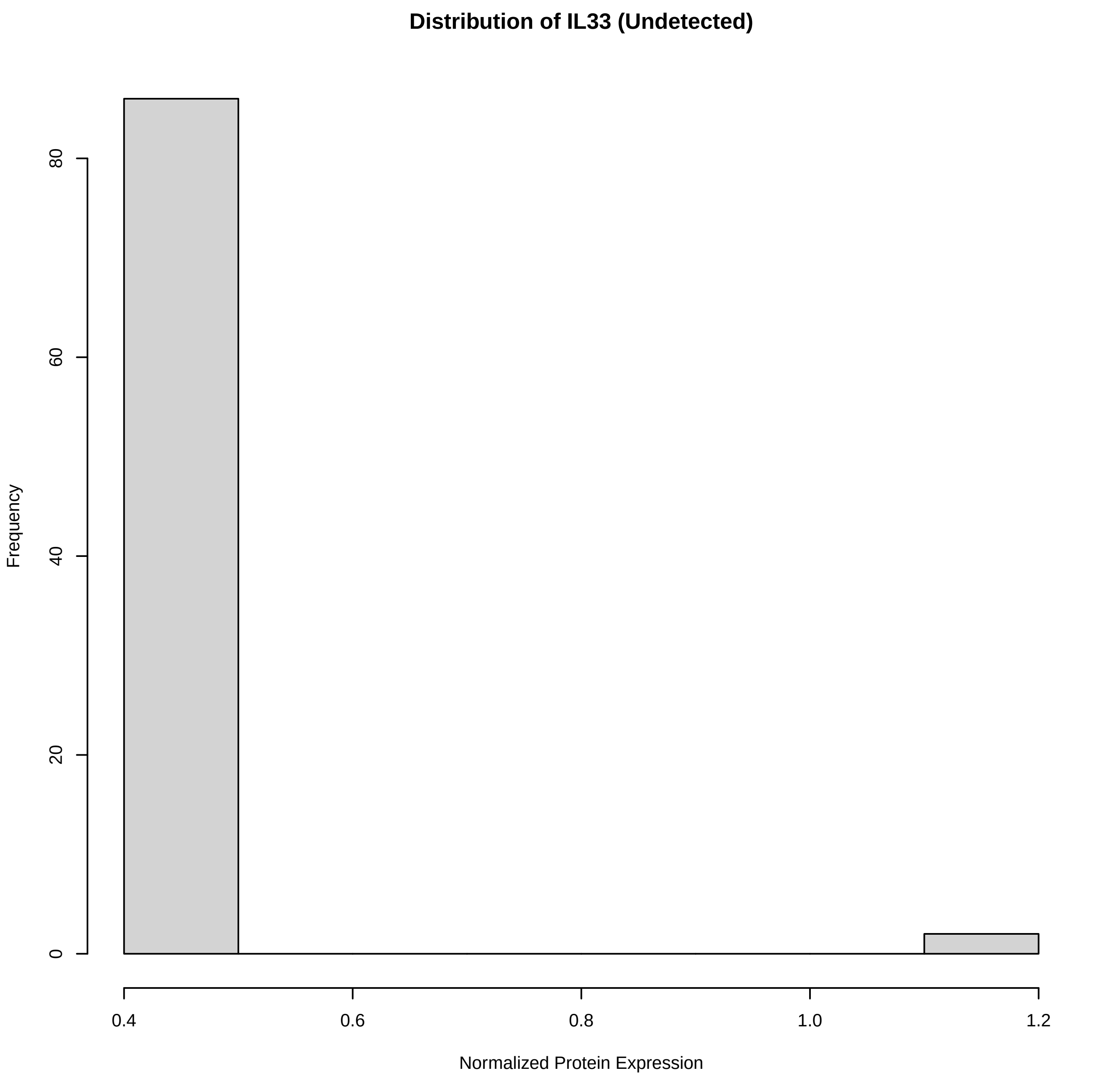
Distribution of IL33 (Undetected)
Frequency
0
20
40
60
80
0.4
0.6
0.8
1.0
1.2
Normalized Protein Expression

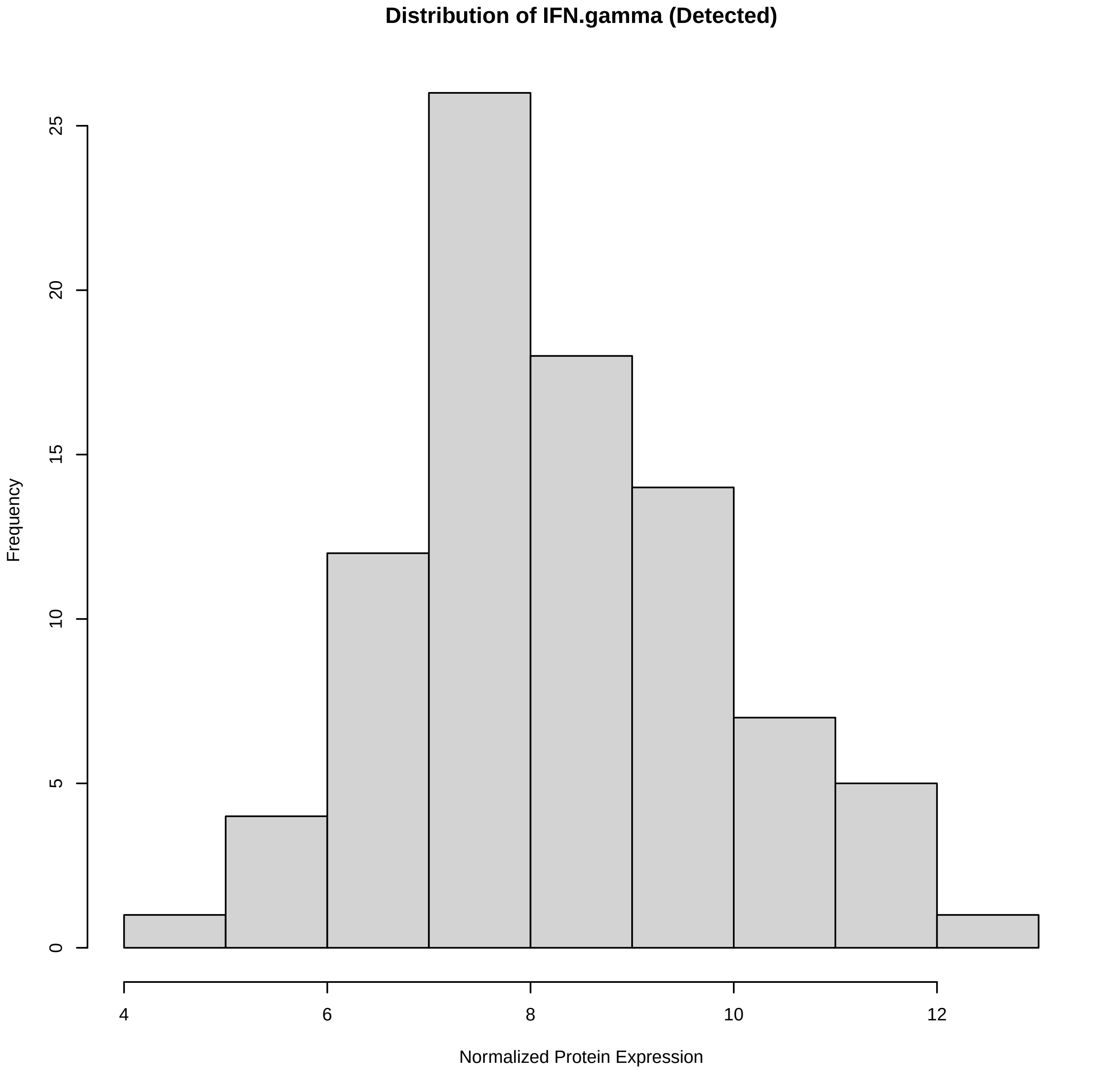

Distribution of IFN.gamma (Detected)
Frequency
0
5
10
15
20
25
4
6
8
10
12
Normalized Protein Expression

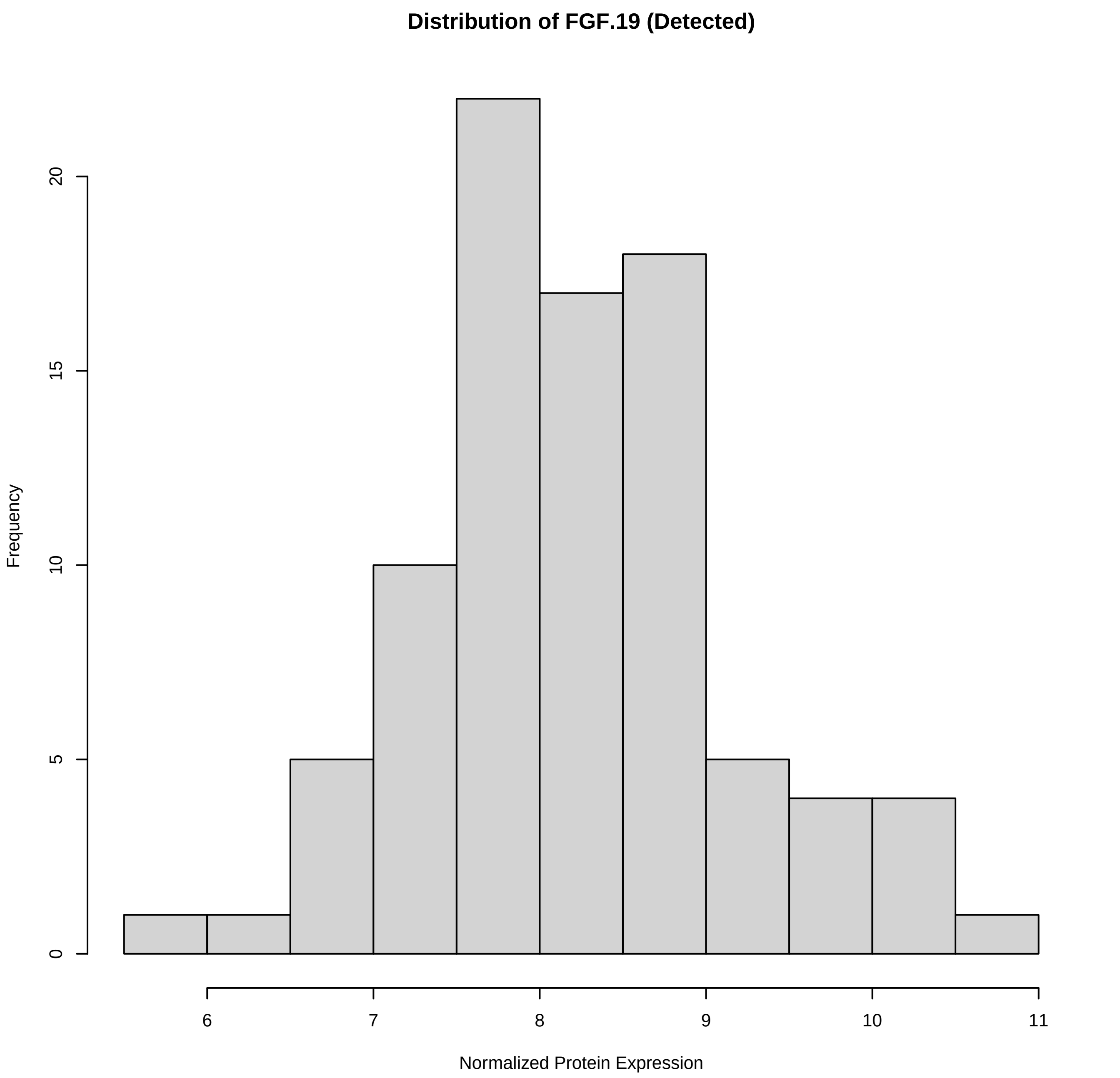

Distribution of FGF.19 (Detected)
Frequency
0
5
10
15
20
6
7
8
9
10
11
Normalized Protein Expression

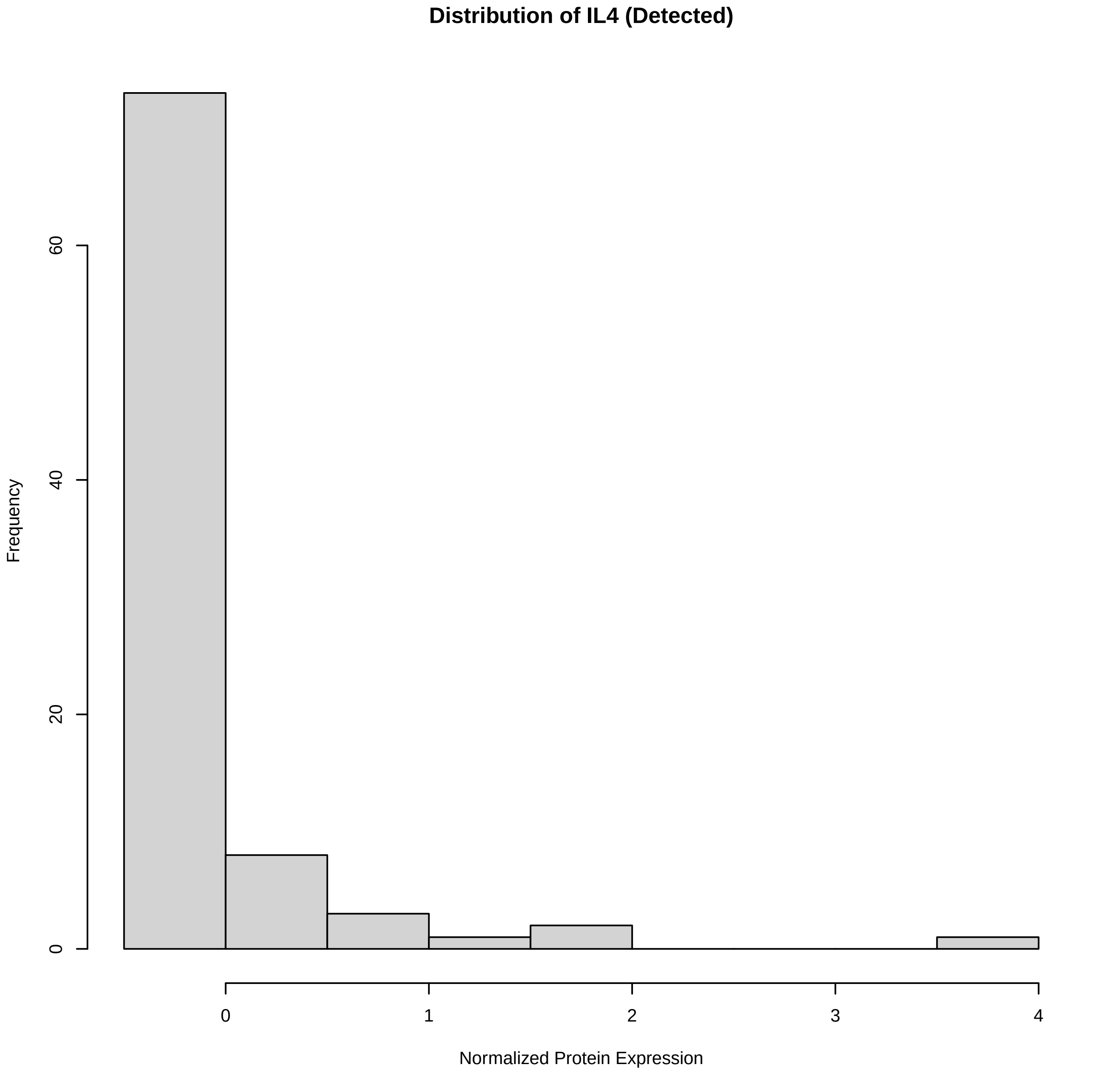
Distribution of IL4 (Detected)
Frequency
0
20
40
60
0
1
2
3
4
Normalized Protein Expression

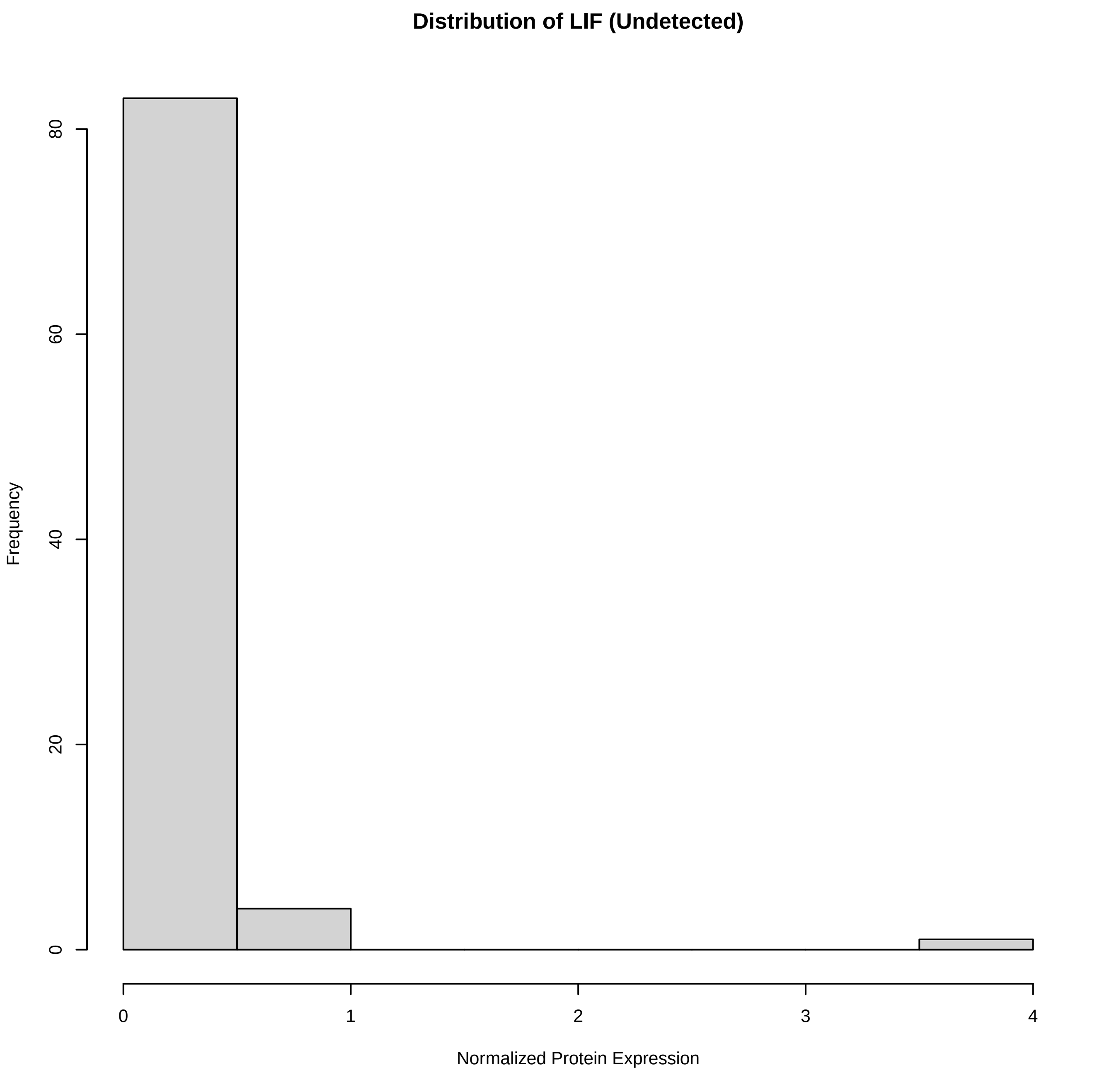
Distribution of LIF (Undetected)
Frequency
0
20
40
60
80
0
1
2
3
4
Normalized Protein Expression

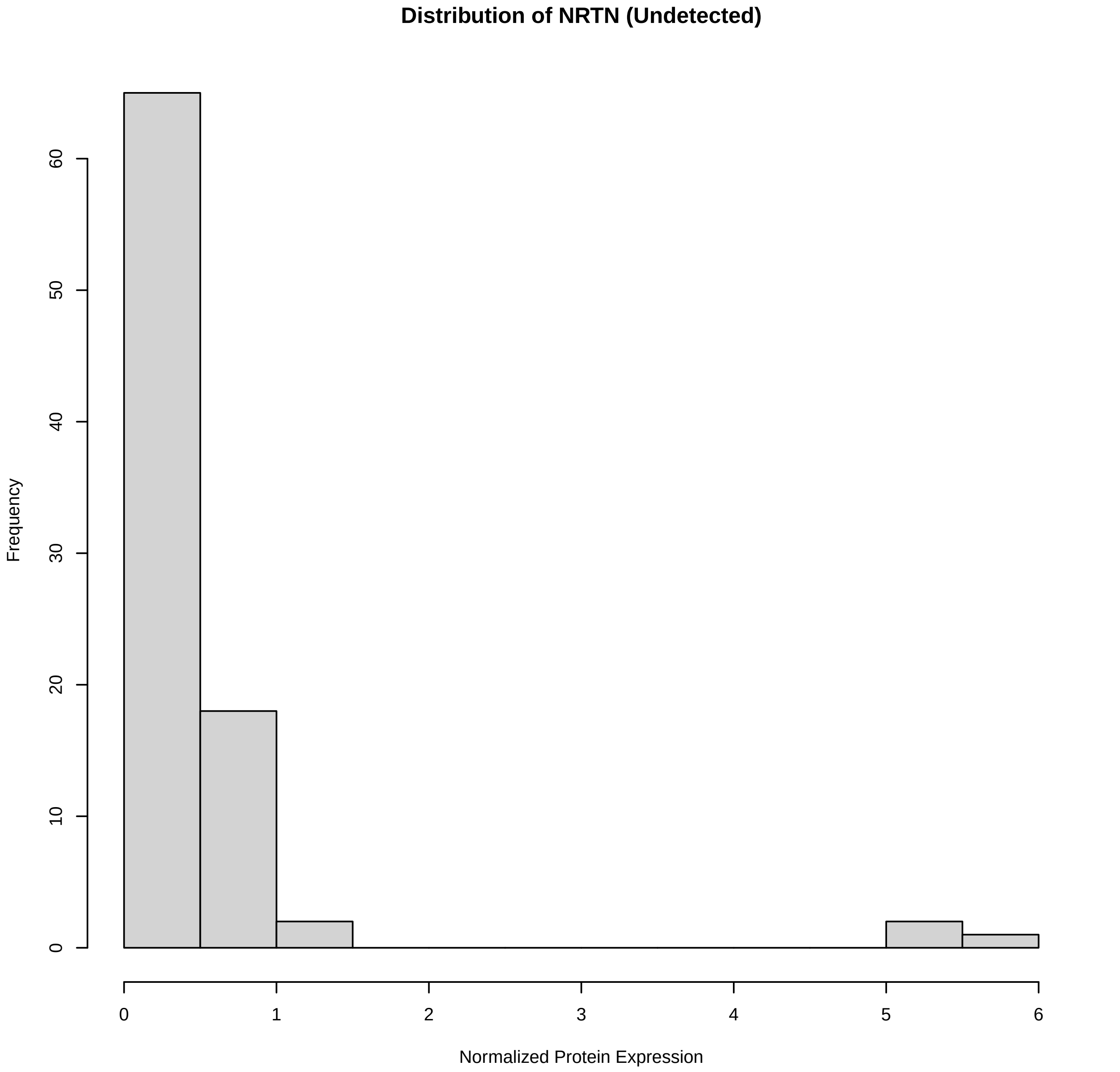
Distribution of NRTN (Undetected)
Frequency
0
10
20
30
40
50
60
0
1
2
3
4
5
6
Normalized Protein Expression

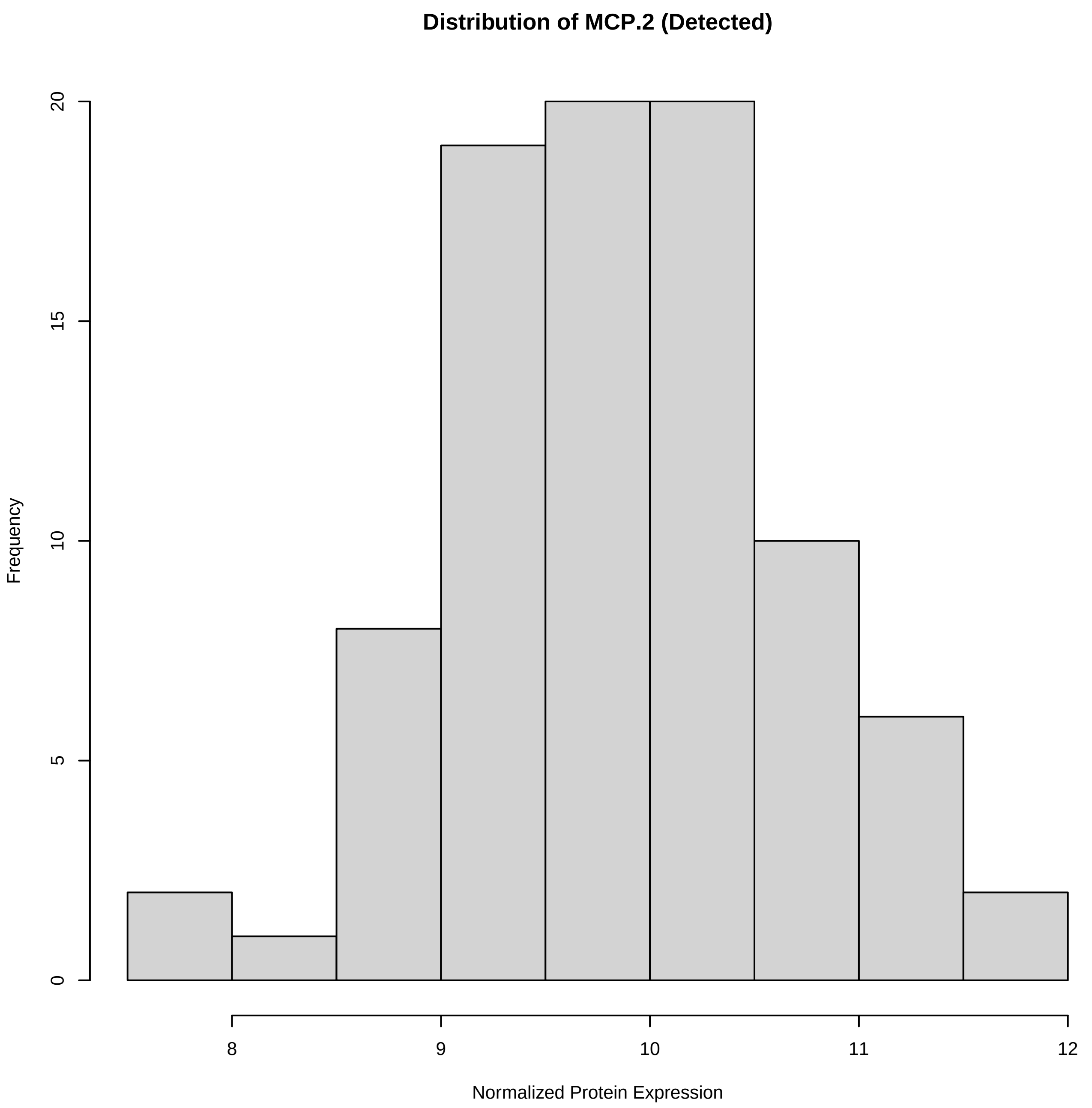

Distribution of MCP.2 (Detected)
Frequency
0
5
10
15
20
8
9
10
11
12
Normalized Protein Expression

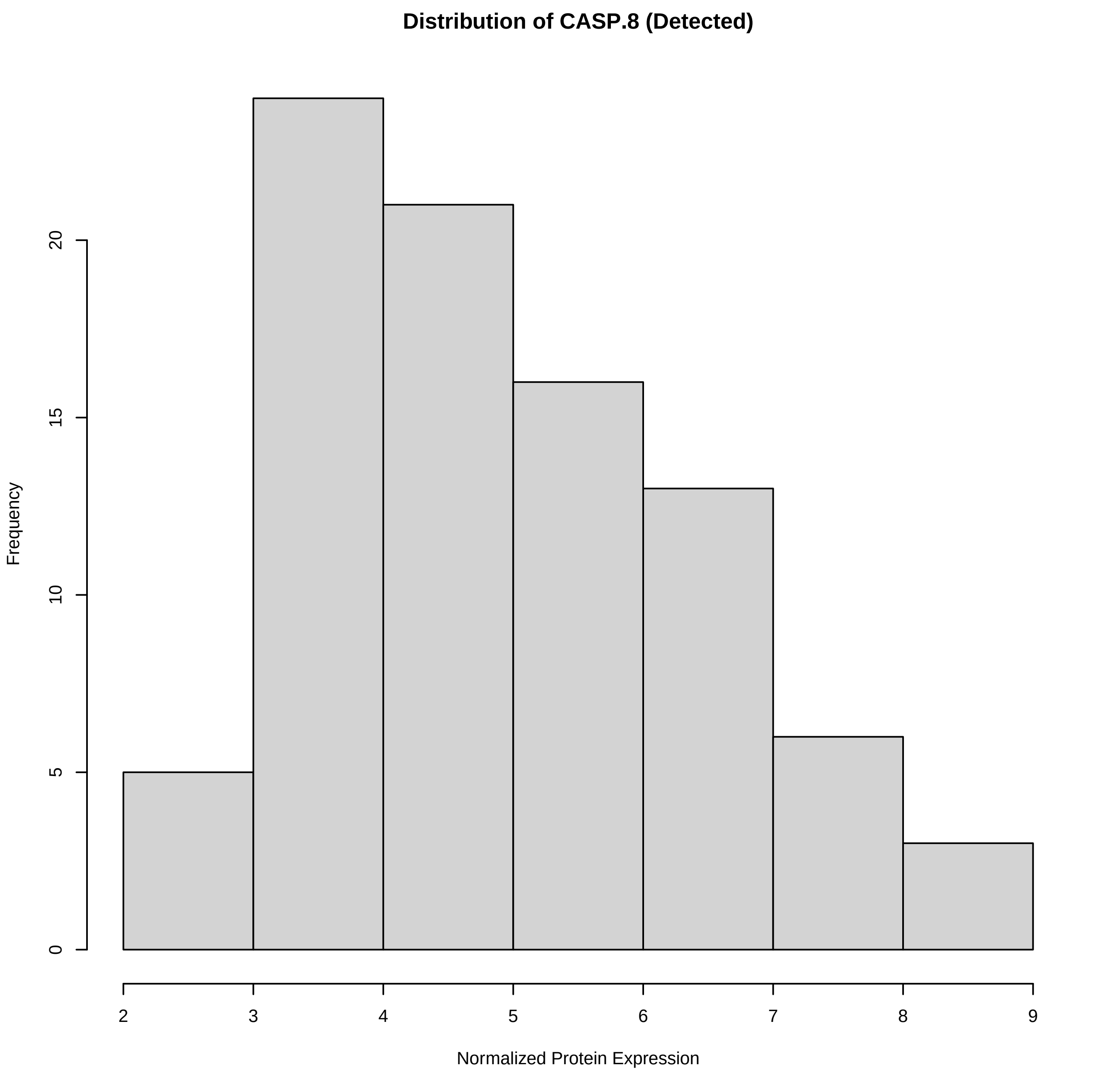
Distribution of CASP.8 (Detected)
Frequency
0
5
10
15
20
2
3
4
5
6
7
8
9
Normalized Protein Expression

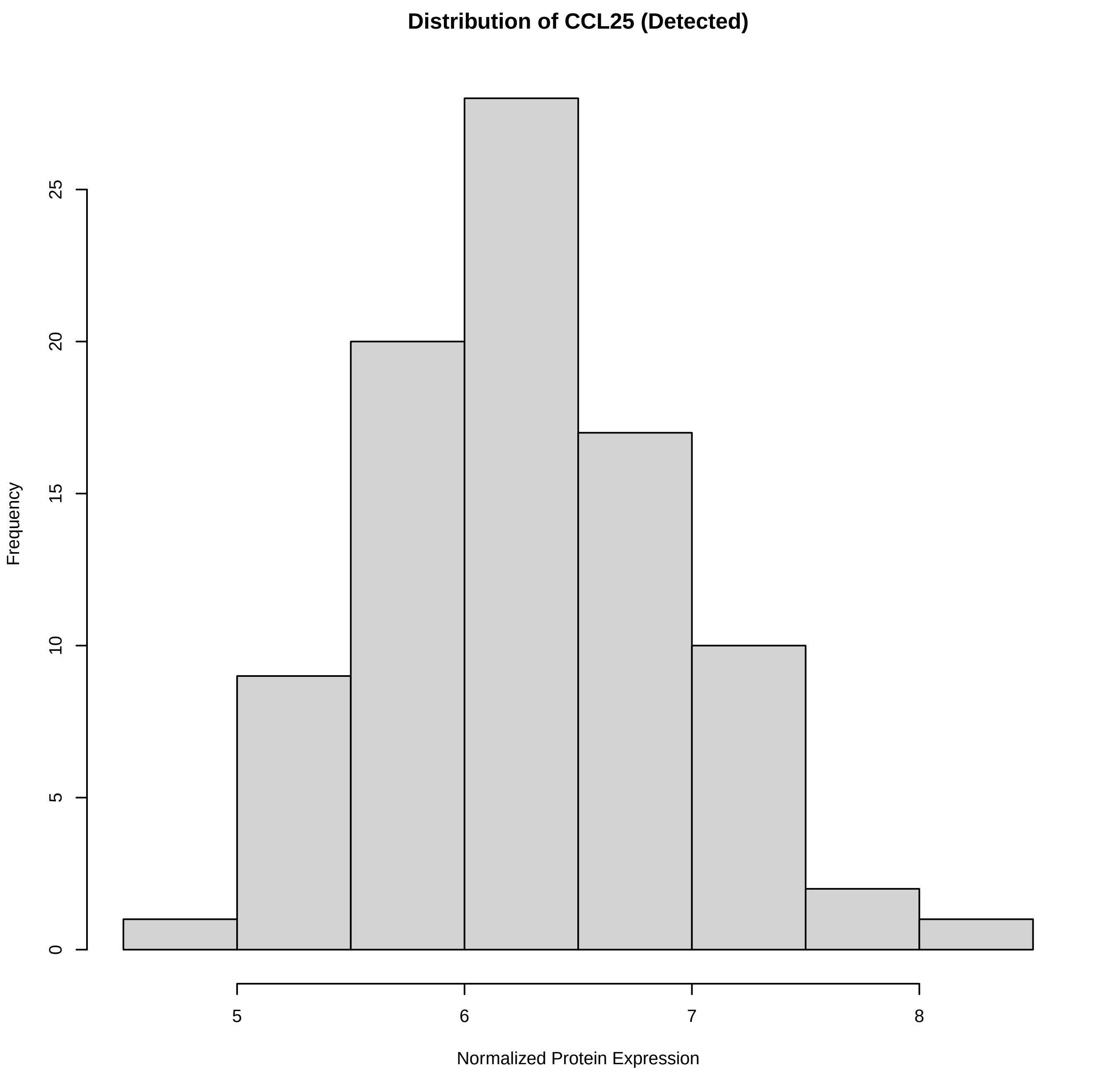
Distribution of CCL25 (Detected)
Frequency
0
5
10
15
20
25
5
6
7
8
Normalized Protein Expression

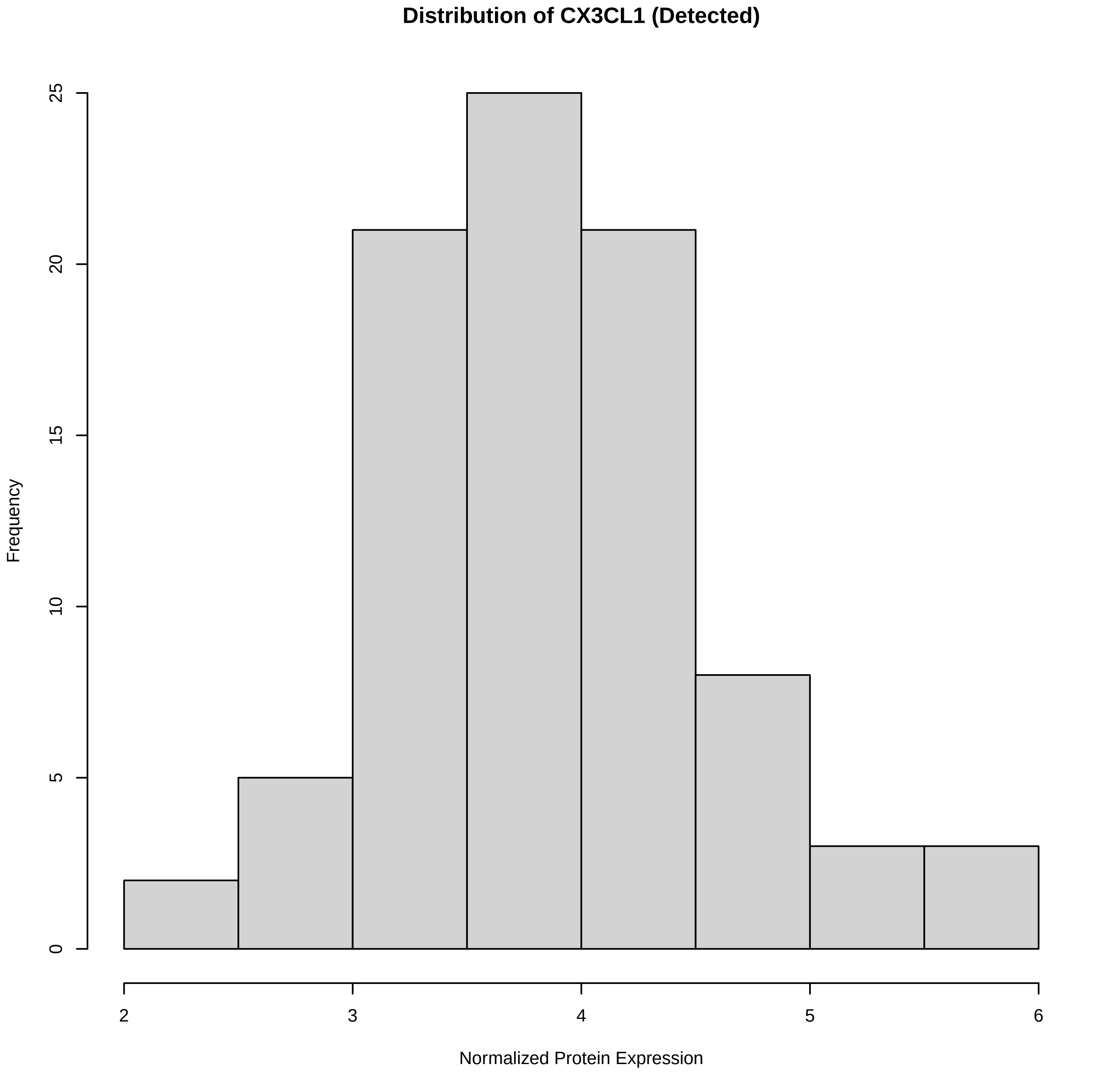
Distribution of CX3CL1 (Detected)
Frequency
0
5
10
15
20
25
2
3
4
5
6
Normalized Protein Expression

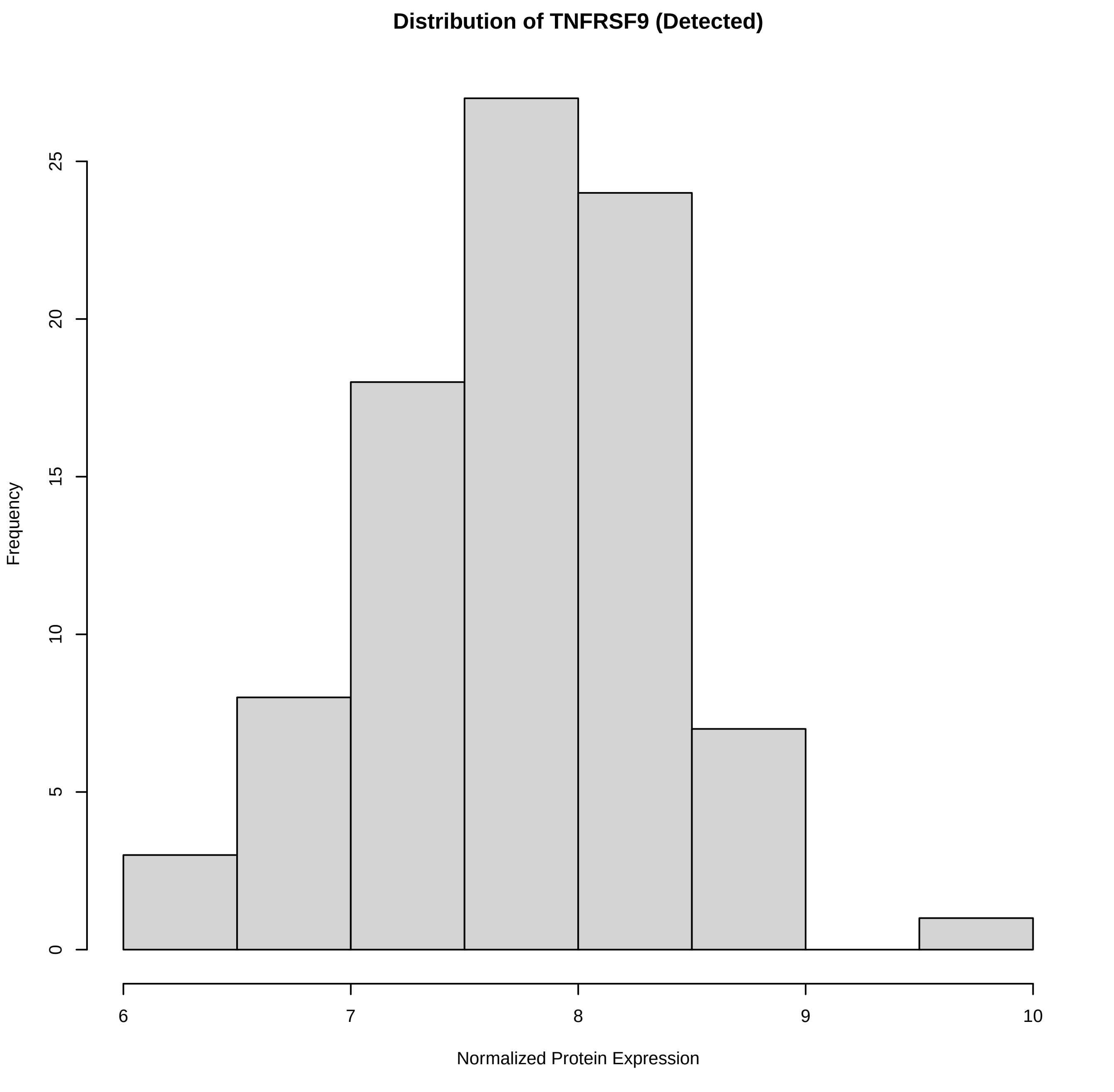
Distribution of TNFRSF9 (Detected)
Frequency
0
5
10
15
20
25
6
7
8
9
10
Normalized Protein Expression

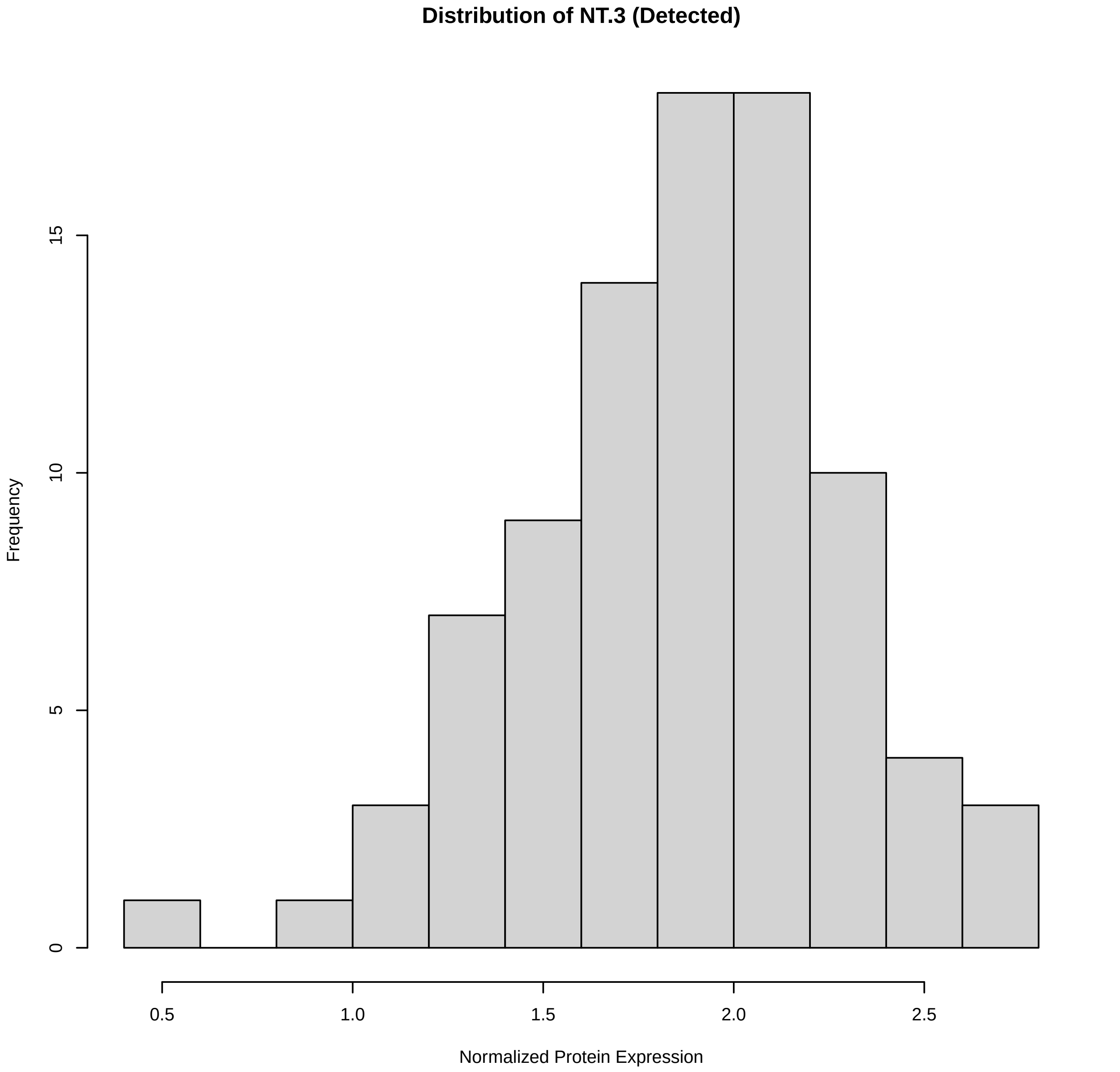
Distribution of NT.3 (Detected)
Frequency
0
5
10
15
0.5
1.0
1.5
2.0
2.5
Normalized Protein Expression

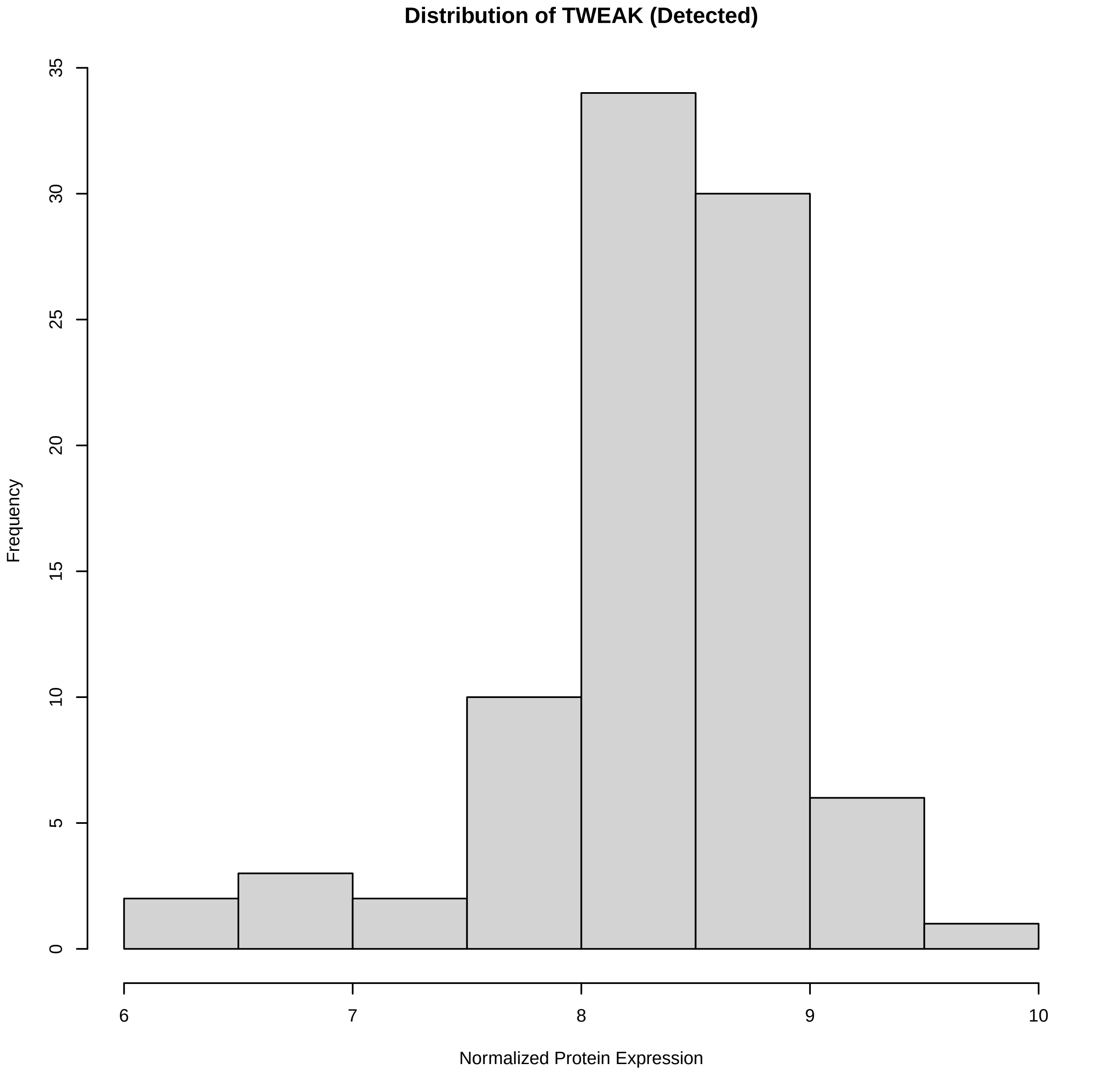
Distribution of TWEAK (Detected)
Frequency
0
5
10
15
20
25
30
35
6
7
8
9
10
Normalized Protein Expression

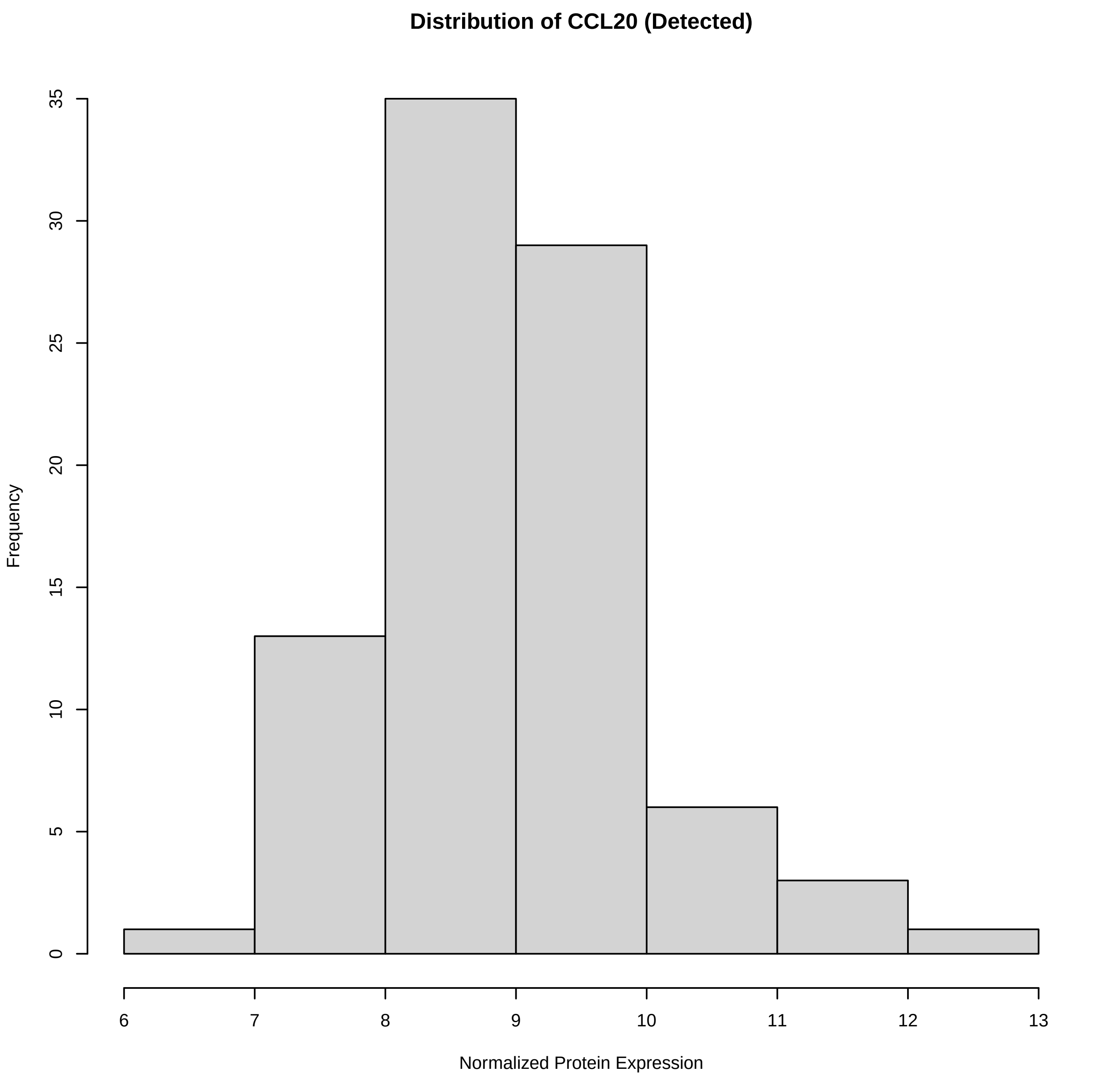

Distribution of CCL20 (Detected)
Frequency
0
5
10
15
20
25
30
35
6
7
8
9
10
11
12
13
Normalized Protein Expression

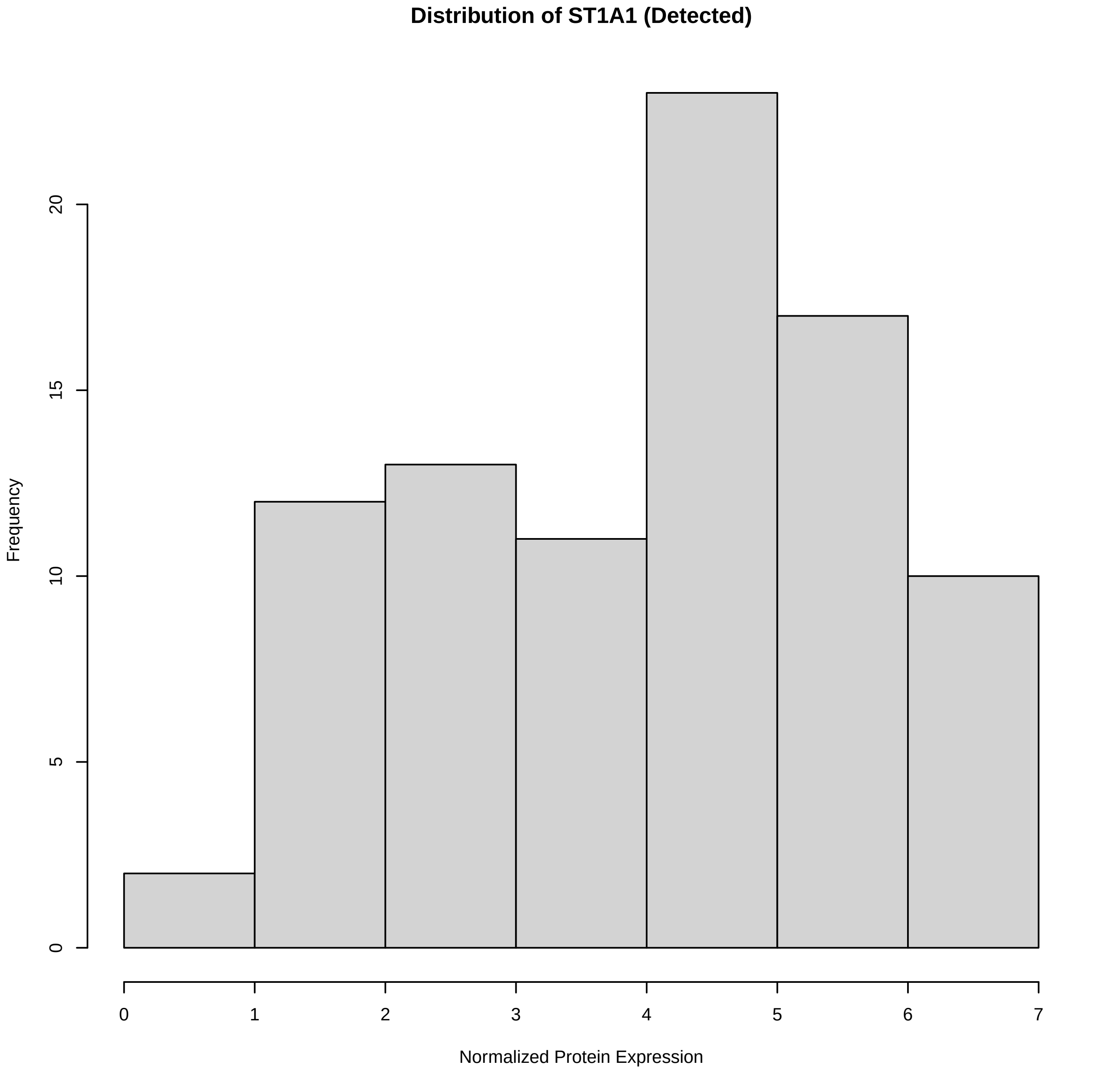
Distribution of ST1A1 (Detected)
Frequency
0
5
10
15
20
0
1
2
3
4
5
6
7
Normalized Protein Expression

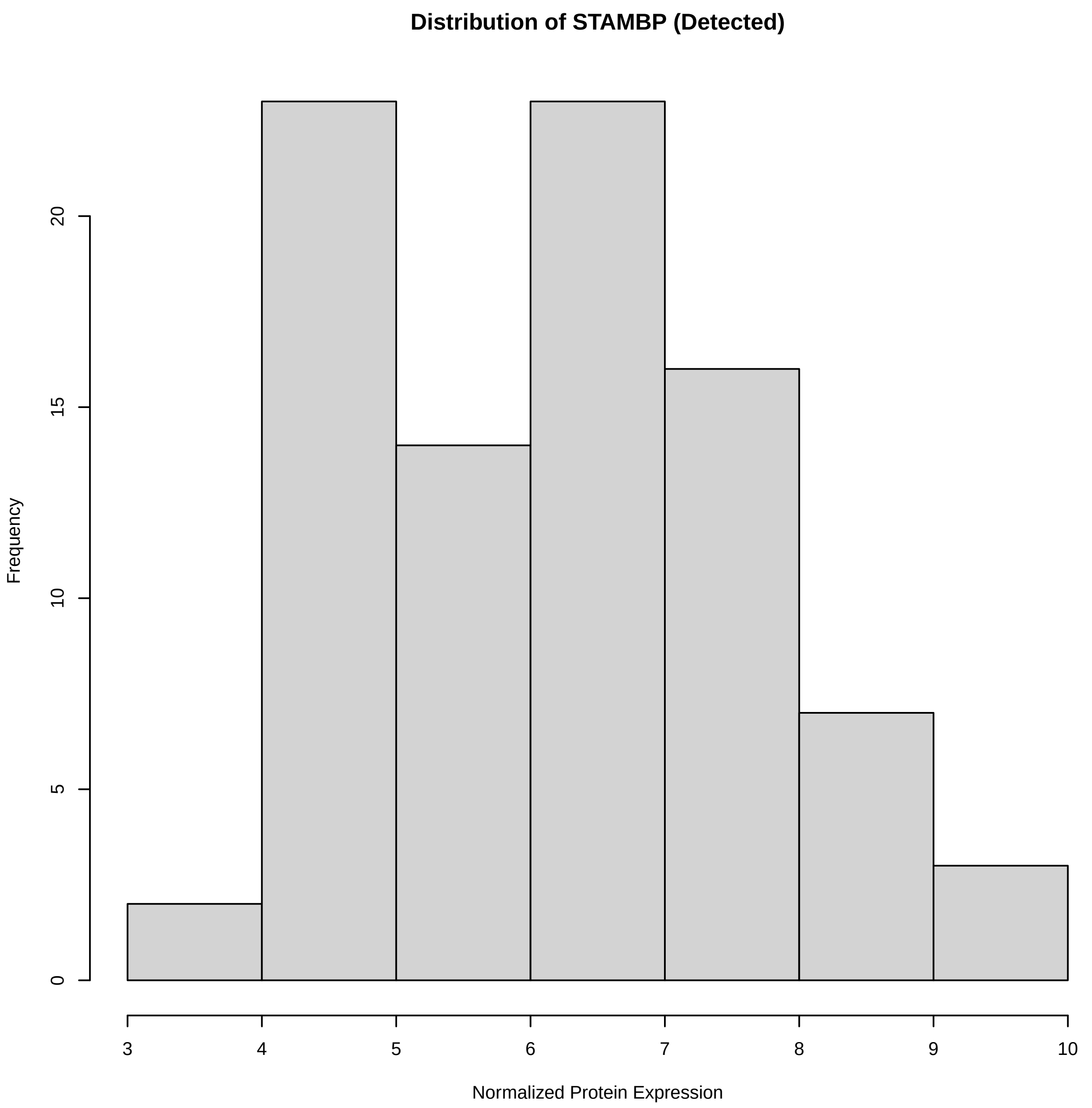
Distribution of STAMBP (Detected)
Frequency
0
5
10
15
20
3
4
5
6
7
8
9
10
Normalized Protein Expression

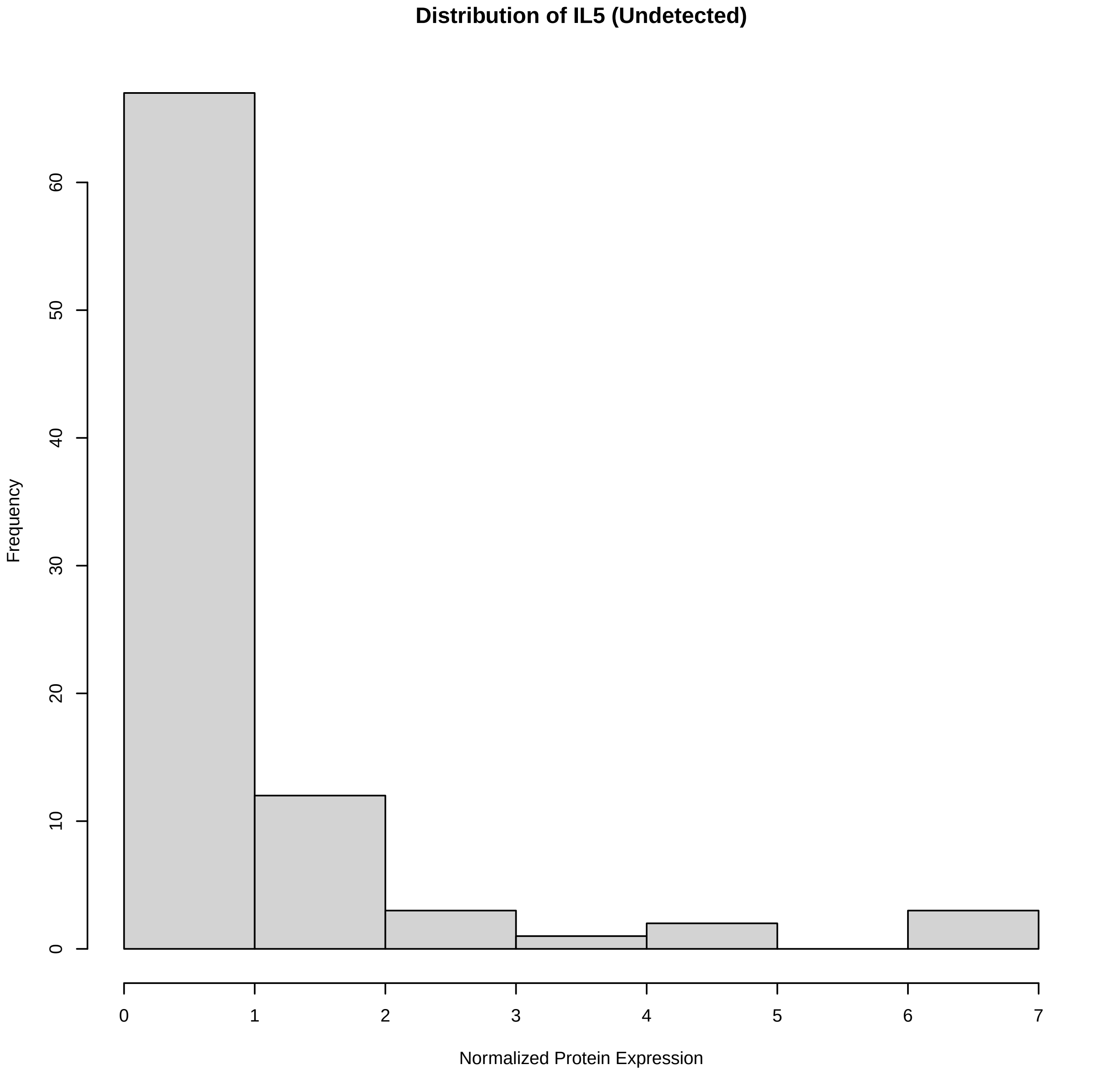
Distribution of IL5 (Undetected)
Frequency
0
10
20
30
40
50
60
0
1
2
3
4
5
6
7
Normalized Protein Expression

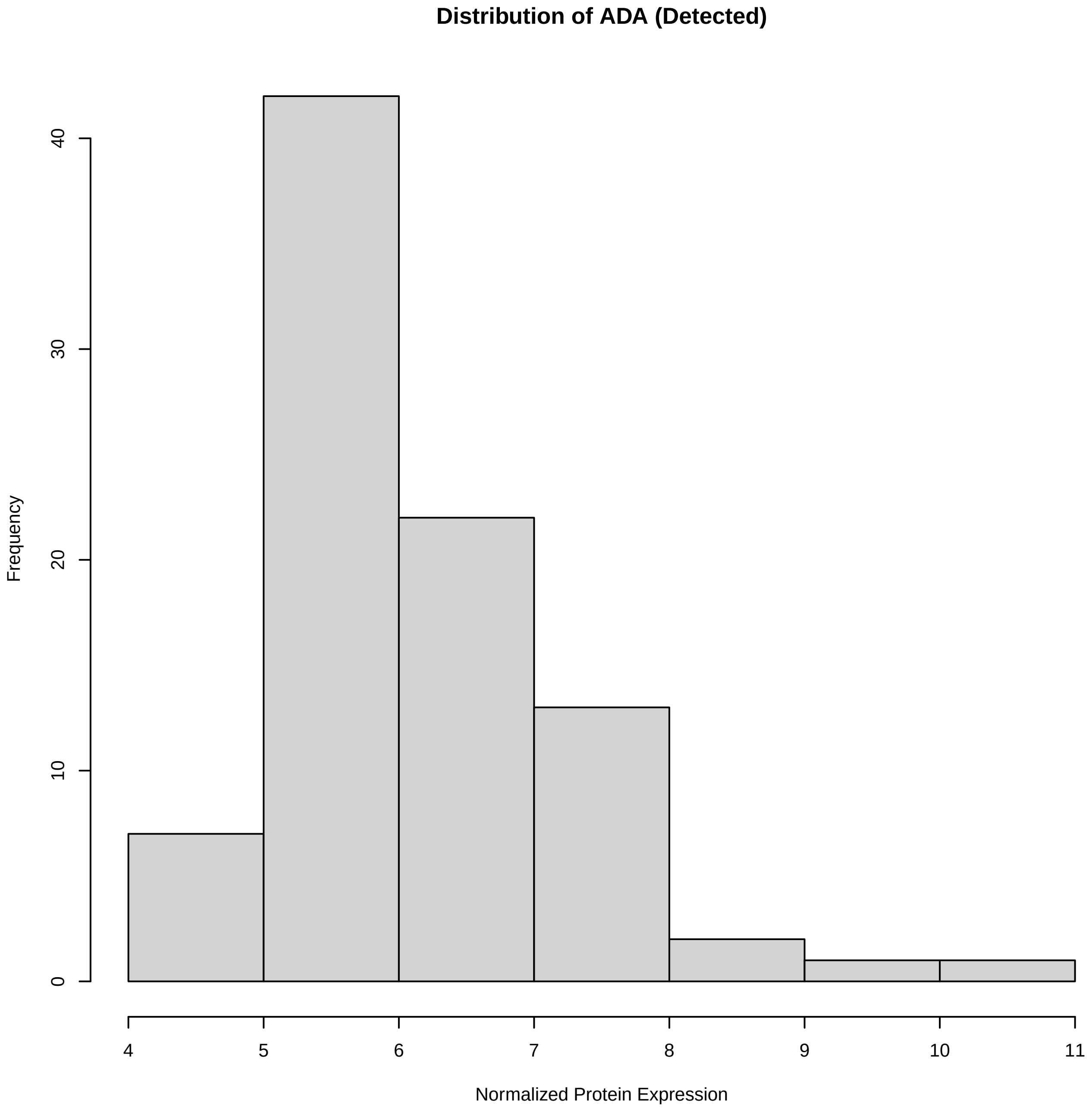

Distribution of ADA (Detected)
Frequency
0
10
20
30
40
4
5
6
7
8
9
10
11
Normalized Protein Expression

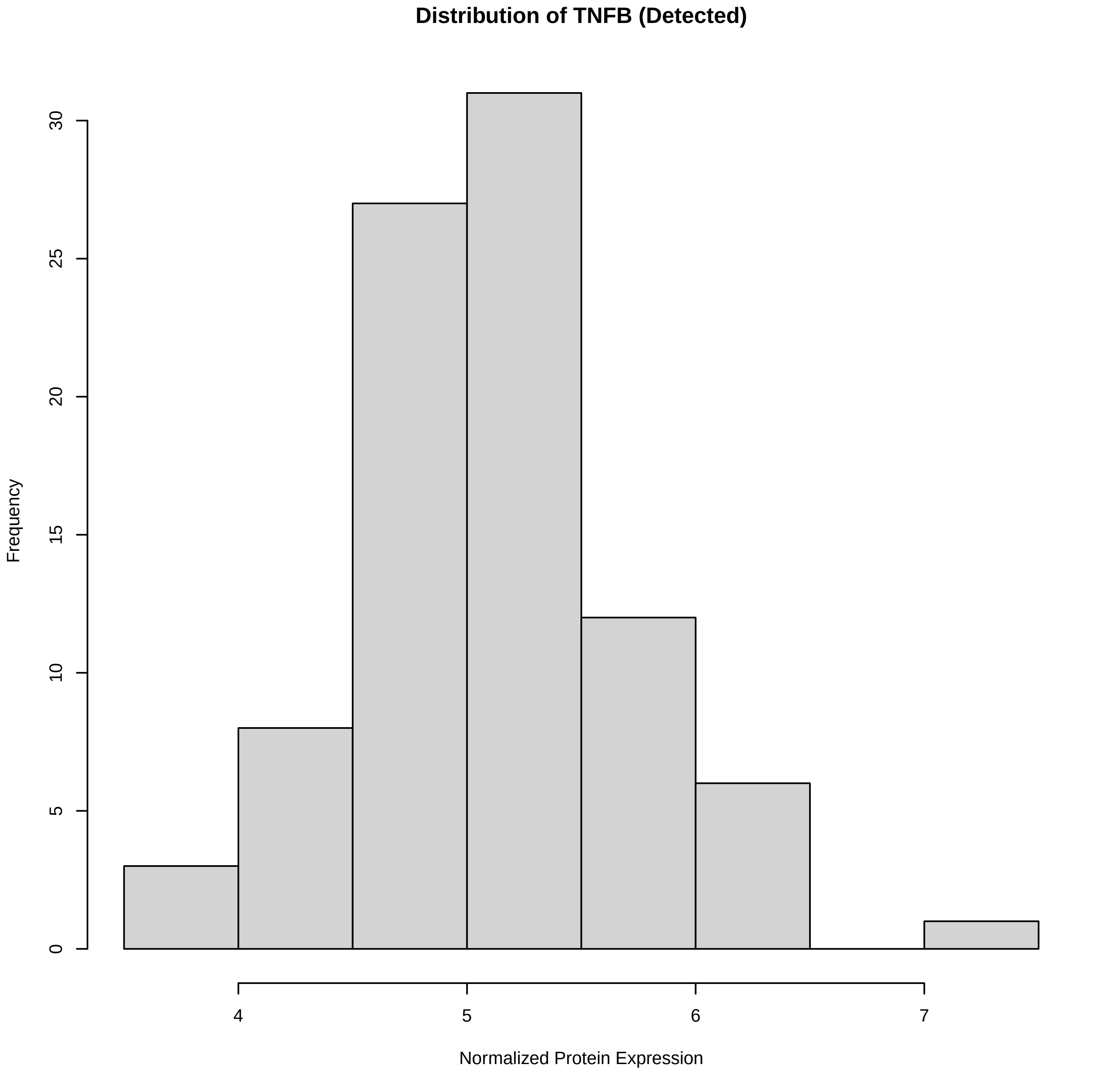

Distribution of TNFB (Detected)
Frequency
0
5
10
15
20
25
30
4
5
6
7
Normalized Protein Expression

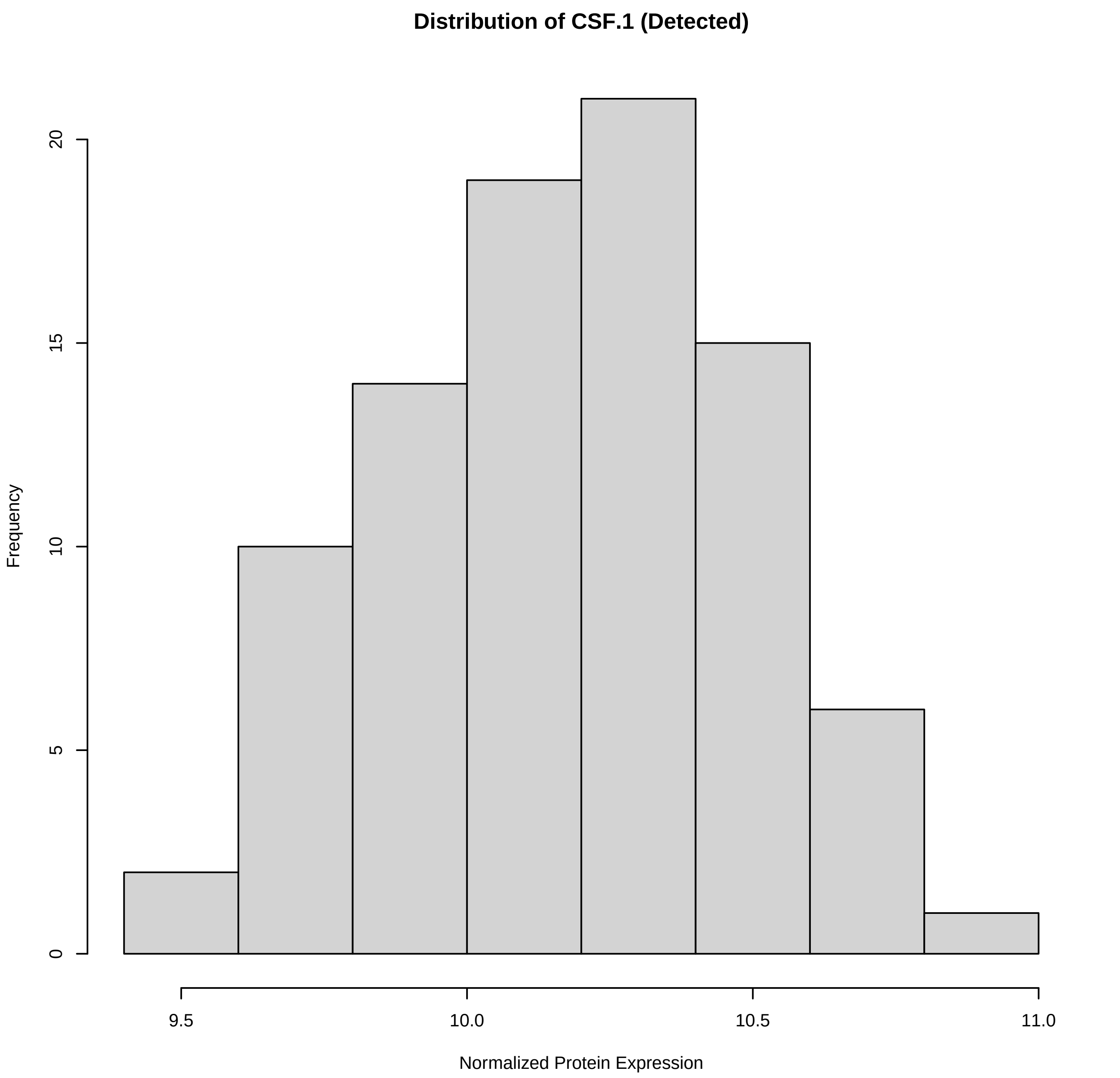
Distribution of CSF.1 (Detected)
Frequency
0
5
10
15
20
9.5
10.0
10.5
11.0
Normalized Protein Expression
